# Light-driven transition-metal-free direct decarbonylation of unstrained diaryl ketones via a dual C–C bond cleavage

**DOI:** 10.1038/s41467-022-29327-z

**Published:** 2022-04-04

**Authors:** Dawei Cao, Mohamad Ataya, Zhangpei Chen, Huiying Zeng, Yong Peng, Rustam Z. Khaliullin, Chao-Jun Li

**Affiliations:** 1 Department of Chemistry, and FRQNT Centre for Green Chemistry and Catalysis, McGill University, 801 Sherbrooke St. West, Montreal, QC H3A 0B8 Canada; 2 Key Laboratory of Magnetism and Magnetic Materials of the Ministry of Education, School of Physical Science and Technology and Electron Microscopy Centre, Lanzhou University, 730000 Lanzhou, P. R. China; 3 The State Key Laboratory of Applied Organic Chemistry, Lanzhou University, 730000 Lanzhou, P. R. China

**Keywords:** Synthetic chemistry methodology, Photocatalysis

## Abstract

The cleavage and formation of carbon−carbon bonds have emerged as powerful tools for structural modifications in organic synthesis. Although transition−metal−catalyzed decarbonylation of unstrained diaryl ketones provides a viable protocol to construct biaryl structures, the use of expensive catalyst and high temperature (>140 ^o^C) have greatly limited their universal applicability. Moreover, the direct activation of two inert C − C bonds in diaryl ketones without the assistance of metal catalyst has been a great challenge due to the inherent stability of C − C bonds (nonpolar, thermo-dynamically stable, and kinetically inert). Here we report an efficient light-driven transition-metal-free strategy for decarbonylation of unstrained diaryl ketones to construct biaryl compounds through dual inert C − C bonds cleavage. This reaction featured mild reaction conditions, easy-to-handle reactants and reagents, and excellent functional groups tolerance. The mechanistic investigation and DFT calculation suggest that this strategy proceeds through the formation of dioxy radical intermediate via a single-electron-transfer (SET) process between photo-excited diaryl ketone and DBU mediated by DMSO, followed by removal of CO_2_ to construct biaryl compounds.

## Abstract (web summary)

The cleavage and formation of carbon−carbon bonds is an important strategy for structural modifications in organic syntheses. Herein, the authors present a photoinduced method to construct biaryl compounds through dual inert C−C bond cleavage.

## Introduction

**Fig. 1:**
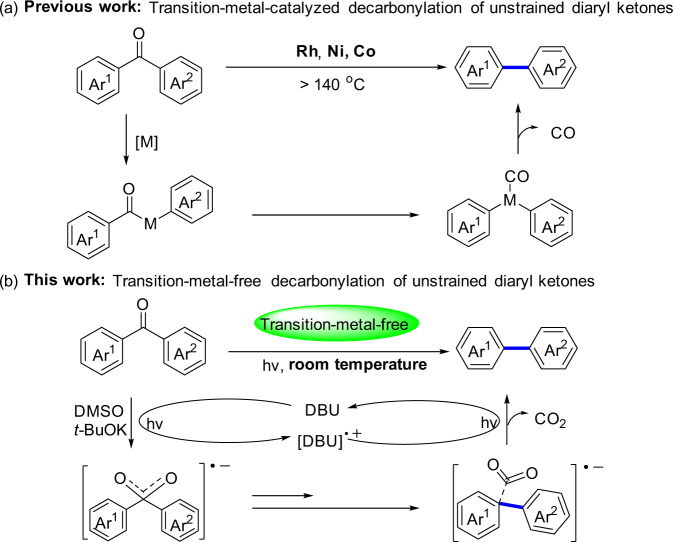
Strategies for decarbonylation of unstrained aryl ketones. **a** Transition-metal-catalyzed decarbonylation of unstrained diaryl ketones. **b** Transition-metal-free decarbonylation of unstrained diaryl ketones.

The cleavage and formation of carbon−carbon bonds has emerged as an important strategy for structural modifications in organic syntheses, biomass conversions, and medicinal chemistry^1–4^. Therefore, many research efforts have been devoted to developing novel and efficient methodologies in this field^5^. Among such strategies, a decarbonylative process via C–C bond cleavage of carbonyl compounds, such as aldehydes as well as aroyl chlorides, thioesters, esters, and anhydrides, has recently emerged as a particularly efficient and powerful approach for C–C bond formation^6,7^. In particular, the highly selective functionalization of diaryl ketones, through a decarbonylation process to synthesize biaryls, has attracted widespread attention because of the broad application of biphenyls in a wide range of fields such as pharmaceuticals, agrochemicals, pigments, natural products and polymers^8,9^. However, there still remains one of the most difficult challenges for the direct decarbonylation of unstrained diaryl ketones through dual inert C–C bonds cleavage due to the inherent stability of C–C bonds (nonpolar, thermo-dynamically stable, and kinetically inert)^10,11^. In recent years, the rhodium-, nickel- or cobalt- catalyzed decarbonylation of diaryl ketones to construct biaryl structures has been established with excellent step and atom economy thus far, which involves two steps of oxidative addition, followed by reductive elimination and removal of CO, realizing the cleavage of the dual carbon-carbon bonds of the diaryl ketone (Fig. 1a)^12–17^. However, these methods suffered from several limitations toward decarbonylation of unstrained diaryl ketones such as the requirement of noble metals, high temperature (>140 °C) and extra-directing groups. Therefore, the development of a direct, efficient and transition-metal-free approach for the decarbonylation of unstrained diaryl ketones to form biaryl compounds is still highly desirable.

Over the last decade, photochemical reactions have attracted much attention due to their novel transformation pathways, high efficiency, environmental friendliness and mild reaction conditions^18–26^. Among them, the selective photocatalytic cleavage of strained C–C bonds is an efficient and valuable tool for organic syntheses^27,28^. Compared with strained C–C bond, C–C bond cleavage of noncyclic compounds, especially unstrained diaryl ketones, has been largely neglected.

Here, we show a simple and potent light-driven transition-metal-free direct decarbonylation strategy of unstrained diaryl ketones. Notably, different from the established photodecarbonylation of cyclic ketones^29,30^ and solid-state photodecarbonylation of noncyclic ketones^31,32^, this reaction pathway involves a light-enabled single-electron transfer (SET) between ketone and DBU mediated by DMSO and *t*-BuOK to form a radical anion intermediate, which gives diaryl compounds upon the removal of CO_2_ (Fig. 1b).

## Results

### Reaction optimization

At the outset, the decarbonylation of bis(4-fluorophenyl)methanone (**1u**) in the presence of DBU (1,8-diazabicyclo[5.4.0]undec-7-ene) and *t*-BuOK was carried out under hv (254 nm) irradiation with DMSO as solvent under Ar at room temperature. To our delight, after 24 h of irradiation, this transformation could furnish the desired product 4,4′-difluoro-1,1′-biphenyl (**2u**) in 32% yield, together with side products including 4,4′-difluorodiphenylmethane, 4,4′-difluorobenzhydrol and (2, 2- bis(4- fluorophenyl) vinyl) (methyl) sulfane detected by GC-MS (Table 1, entry 1). However, other light sources such as blue light emitting diode (LED) and compact fluorescent lamp (CFL) showed poor reactivity in this transformation (Table 1, entries 2–3). Notably, exclusion of any components of this reaction, including light source, DBU or *t*-BuOK, did not afford the desired product (Table 1, entries 4–6). Subsequently, the effect of base was investigated and the results demonstrated that, when using other bases (NaOH, KOH or K_2_CO_3_) instead of t-BuOK, the reaction preceded with lower efficiency (Table 1, entries 7–9). Further investigation results showed that the solvent played a vital role in the decarbonylation reaction (Table 1, entries 10–13). The yields of **2u** dramatically decreased when DMSO was replaced by other solvents such as MeCN, H_2_O, 1,4-dioxane and CHCl_3_. Interestingly, a small amount of water (80 μL, equivalent to 4.44 mmol%) was found to be beneficial to this reaction (Table 1, entry 16), whereas a higher or lower amount of water resulted in lower yields (Table 1, entries 14 and 15). Notably, no improvement in yield was observed when using TMEDA (N,N,N′,N′-tetramethylethylenediamine), Et_3_N or DABCO (triethylenediamine) instead of DBU (Table 1, entries 17–19). Decreasing the amount of DBU to 1 equivalent lowered the yield of **2u** to 38% (Table 1, entry 20). When the amount of DBU was increased to three equivalents, there was no obvious improvement on the reaction (Table 1, entry 21). The amount of *t*-BuOK was also important for the reaction: a lower yield (8%) was achieved when 0.5 equiv *t*-BuOK was used (Table 1, entry 22), while increasing *t*-BuOK to 1.25 equiv gave **2u** in 64% yield (Table 1, entry 23) and further increasing the amount of *t*-BuOK to 1.5 equiv decreased the yield slightly (Table 1, entry 24). Furthermore, increasing the reaction time to 36 h offered further improvement, providing **2u** in 70% isolated yield (Table 1, entry 25). However, the reaction gave a lower yield when operated under an air atmosphere (Table 1, entry 26).

**Table 1:** Optimization of the reaction conditions^a^.


Entry	hv	DBU (equiv)	Base	Solvent	2u yield^[b]^/%
1	uv (254 nm)	2	*t-*BuOK	DMSO	32
2	blue LED	2	*t-*BuOK	DMSO	n.p.
3	CFL	2	*t-*BuOK	DMSO	n.p.
4	dark	2	*t-*BuOK	DMSO	n.p.
5	uv (254 nm)	–	*t-*BuOK	DMSO	n.p.
6	uv (254 nm)	2	–	DMSO	n.p.
7	uv (254 nm)	2	NaOH	DMSO	19
8	uv (254 nm)	2	KOH	DMSO	18
9	uv (254 nm)	2	K_2_CO_3_	DMSO	n.p.
10	uv (254 nm)	2	*t-*BuOK	MeCN	n.p.
11	uv (254 nm)	2	*t-*BuOK	H_2_O	n.p.
12	uv (254 nm)	2	*t-*BuOK	dioxane	n.p.
13	uv (254 nm)	2	t*-*BuOK	CHCl_3_	n.p.
14^[c]^	uv (254 nm)	2	*t-*BuOK	DMSO	38
15^[d]^	uv (254 nm)	2	*t-*BuOK	DMSO	42
16^[e]^	uv (254 nm)	2	*t-*BuOK	DMSO	50
17^[e]^	uv (254 nm)	TMEDA	*t-*BuOK	DMSO	18
18^[e]^	uv (254 nm)	Et_3_N	*t-*BuOK	DMSO	trace
19^[e]^	uv (254 nm)	DABCO	*t-*BuOK	DMSO	5
20^[e]^	uv (254 nm)	1	*t-*BuOK	DMSO	38
21^[e]^	uv (254 nm)	3	*t-*BuOK	DMSO	59
22^[e], [f]^	uv (254 nm)	2	*t-*BuOK	DMSO	8
23^[e], [g]^	uv (254 nm)	2	*t-*BuOK	DMSO	64
24^[e], [h]^	uv (254 nm)	2	*t-*BuOK	DMSO	65
25^[e], [g], [i]^	uv (254 nm)	2	*t-*BuOK	DMSO	72 (70)
26^[e], [g], [i], [j]^	uv (254 nm)	2	*t-*BuOK	DMSO	20

^a^General conditions: **1u** (0.1 mmol), DBU (x equiv), base (1 equiv) and solvent (1.5 mL) at rt for 24 h under Ar.

^b^Yields were determined by ^19^F NMR with benzotrifluoride as internal standard; isolated yields in brackets.

^c^H_2_O (40 μL).

^d^H_2_O (100 μL).

^e^H_2_O (80 μL).

^f^*t*-BuOK (0.5 equiv).

^g^*t*-BuOK (1.25 equiv).

^h^*t*-BuOK (1.5 equiv).

^i^36 h.

^j^Under air.

### Investigation of the substrate scope

**Fig. 2:**
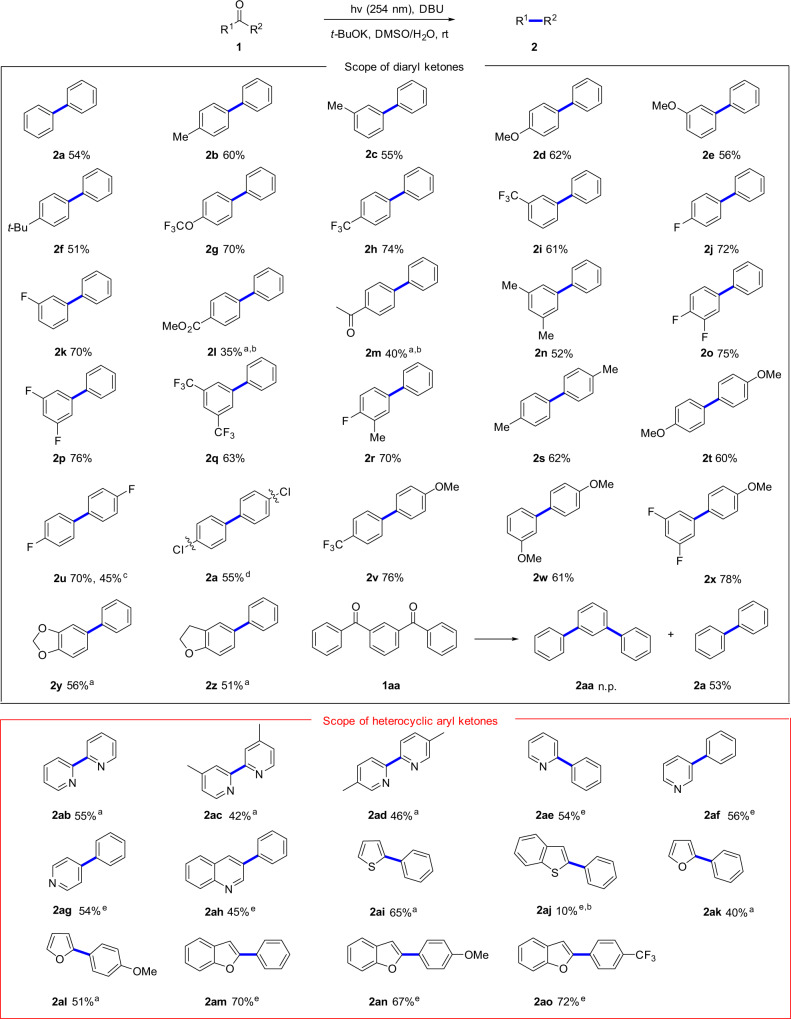
Substrate scope of the decarbonylation reaction. General conditions: **1** (0.1 mmol), DBU (2 equiv.), *t*-BuOK (1.25 equiv.) in DMSO (1.5 mL), H_2_O (80 μL) at rt for 36 h under Ar; ^a^Without H_2_O. ^b^48 h. ^c^The reaction was carried out on a 6.0 mmol scale under modified conditions (see Supplemental Information). ^d^Dechlorination product **2a** was obtained. ^e^NaOH (1.25 equiv) instead of *t*-BuOK, without H_2_O.

Having optimized the reaction conditions, the substrate scope of unstrained diaryl ketones was investigated (Fig. 2). Initially, benzophenone substrate was tested under the standard conditions to deliver the decarbonylation product **2a** in 54% yield. Moreover, this reaction was applicable to various (mono)- substituted unsymmetrical benzophenones with electron-donating groups (Me, OMe, *t*-Bu) and electron-withdrawing groups (OCF_3_, CF_3_, F, CO_2_Me, Ac) at different (m, p) positions of phenyl groups to give the corresponding decarbonylation products (**2b**–**2m**) in good yields. Multi-substituted phenyl groups could be incorporated on the biaryl core regio-selectively, affording the desired products (**2n**–**2r**) in 52–76% yields. Disubstituted symmetrical and unsymmetrical benzophenones could be well tolerated in the reaction regardless of the electronic nature of the substituents (**2s**–**2w**). Notably, when the chloro-containing substrate was introduced, the product biphenyl (**2a**) could be obtained through the cleavage of C–Cl bonds, and this result was consistent with our previous work^33^. In addition, the unsymmetrical diaryl ketone containing 3,4-difluoro and 4′-OMe groups was also viable to undergo this decarbonylation reaction to afford the corresponding product **2x** in 78% yield. Polycyclic diaryl ketones also acted as suitable substrates for this transformation (**2y**–**2z**). However, for the aryl ketone bearing two benzoyl groups (**1aa**), no corresponding product **2aa** was detected; instead, it gave both debenzoylated and decarbonylated product (**2a**) in 53% yield.

After exploring the reaction scope with different diaryl ketones, we turned our attention to apply this strategy to various heterocyclic aryl ketones (Fig. 2). Delightfully, the corresponding bipyridines (**2ab**–**2ad**) were obtained in moderate yields when different substituted di(2-pyridinyl)methanones were subjected to the reaction in the absence of water, which can provide a potential method for the synthesis of bipyridyl ligands. Pyridines bearing phenyl substituent at different positions reacted smoothly and the corresponding products (**2ae**–**2ag**) were generated with NaOH as base instead of *t*-BuOK and in the absence of water. In addition, phenyl(quinolin-3-yl)methanone was also compatible with this transformation (**2ah**). Sulfur-containing heterocyclic aryl ketones were also tolerated in this reaction and successfully transformed into the desired products under the modified conditions (**2ai**–**2aj**). Furan substrates with phenyl groups, including furanylphenylmethanone and 2-(4-methoxybenzoyl) furan were also investigated, delivering the corresponding products (**2ak**–**2al**) with moderate yields. Furthermore, 2-phenylfuranyl ketones bearing either electron-rich or electron-deficient substituents at the para-position of the aryl ring proceeded well and gave the desired products **2am**–**2ao** in good yields.

### Applications

**Fig. 3:**
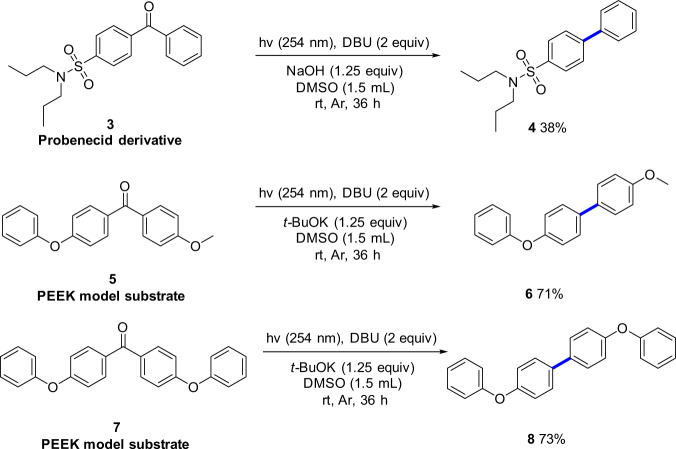
Applications. Conversion of probenecid derivative and polyetheretherketone (PEEK) model compounds.

To further demonstrate the applicability of this decarbonylative system in late stage derivatization of pharmaceuticals, the probenecid derivative **3** (Fig. 3) was tested with NaOH instead of *t*-BuOK as the base in the absence of water. The corresponding biaryl product **4** was obtained in 38% yield, highlighting the advantage of the direct decarbonylation strategy of unstrained diaryl ketones. Polyetheretherketone (PEEK) is widely used as an important engineering plastic but its disposal leads to increasing accumulation in the environment as waste^34^. Therefore, converting disused PEEK into sustainable aromatic compounds has attracted widespread attention for social, economic, and resource sustainability. Thus, the PEEK model compounds **5** and **7** (Fig. 3) were tested with this strategy and, gratifyingly, the desired biaryl products **6** and **8** were obtained in good yields.

### Mechanistic studies

**Fig. 4:**
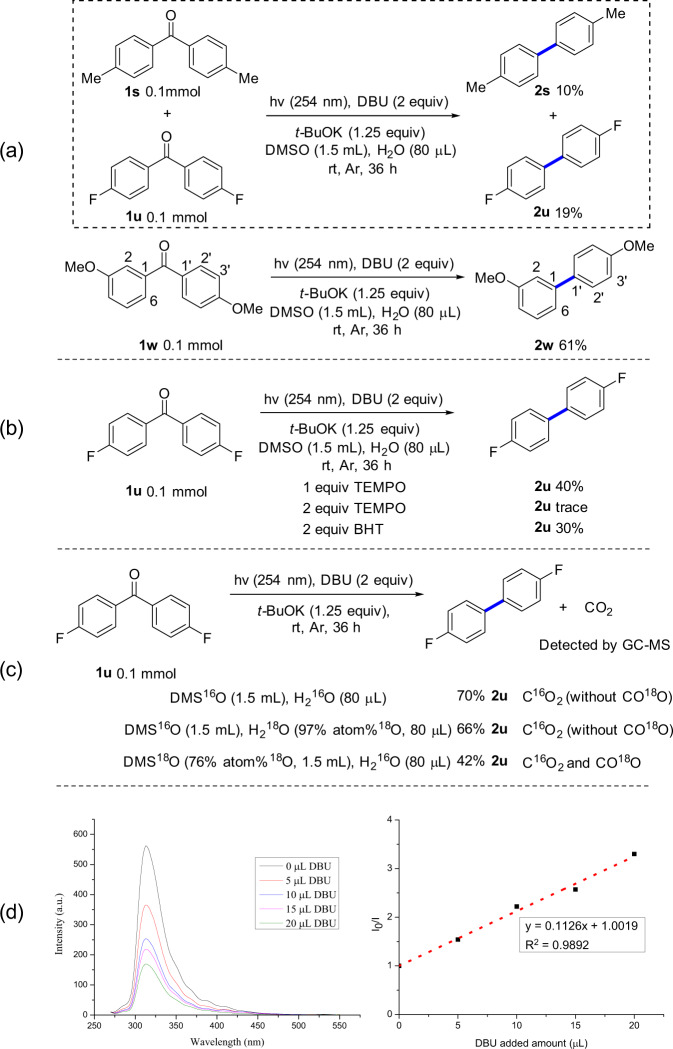
Mechanistic studies. **a** Intermolecular and Intramolecular competition experiments. **b** Radical trapping experiments. **c** [^18^O]-Labelling experiments. **d** Fluorescence quenching experiments.

Next, several preliminary experiments were performed to obtain some additional insights into the mechanistic aspects of this reaction (Fig. 4). Firstly, intermolecular competition experiment using the mixture of **1s** and **1u** was performed under standard conditions. Only the desired products **2s** and **2u** were obtained and no cross-coupling decarbonylation product was detected. Together with the result using **1w** as the substrate in which only product **2w** was obtained without detecting coupling product at other positions (e.g., 2, 6, 2′, 3′) (Fig. 4a), it suggests that the photo-induced decarbonylation occurred through an intramolecular process. Secondly, radical trap experiments were conducted: the yield of **2u** was significantly reduced when 1 equiv of radical inhibitor TEMPO (2,2,6,6-tetramethyl-1-piperidinyloxy) was added into the reaction system under standard reaction conditions; when 2 equiv of TEMPO or BHT (2,6-di-tert-butyl-4-methylphenol) were used, a trace amount and 30% yield of **2u** were obtained, respectively (Fig. 4b), demonstrating the involvement of a radical process of the reaction. Thirdly, isotope labelling experiments were carried out. A small amount of C^16^O_2_ was detected when **1u** was used as a substrate to perform the decarbonylation reaction under standard conditions. Then we verified the source of oxygen in carbon dioxide with H_2_^18^O and DMS^18^O, and the results showed that CO^18^O was detected in the presence of DMS^18^O. These ^18^O-labelling experiments suggested that DMSO may be involved in the decarbonylation process, so that the carbonyl group of the ketone is finally removed in the form of carbon dioxide (Fig. 4c). Finally, from the fluorescence quenching experiments, the fluorescence of benzophenone could be efficiently quenched by DBU, indicating that there is a strong interaction between the excited benzophenone and DBU (Fig. 4d, see Supplemental Information for details).

**Fig. 5:**
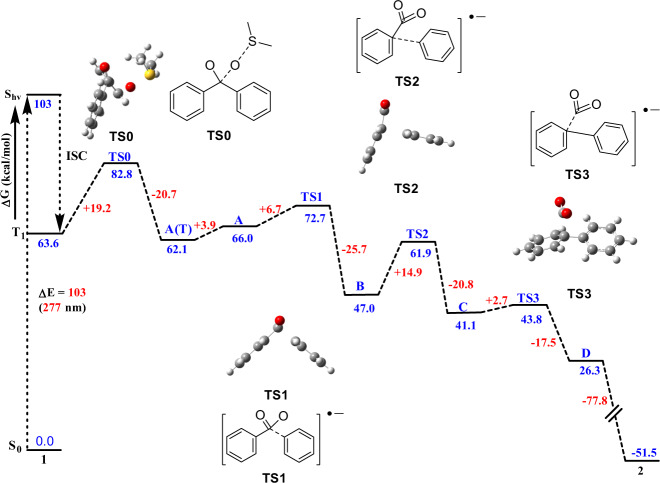
DFT calculation. B3LYP/6-31 + +G(d,p) free energy profile of the reaction.

**Fig. 6:**
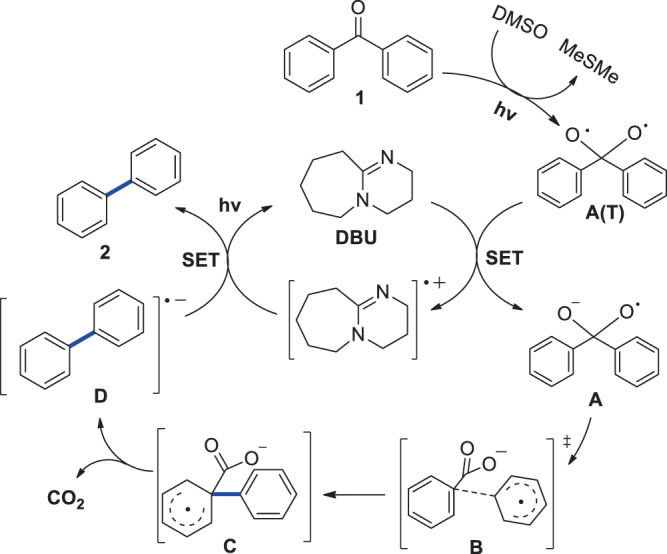
Proposed mechanism. Possible reaction mechanism of decarbonylation of diaryl ketones.

Density functional theory (DFT) calculations were carried out to gain insights into the mechanism of the transformation. The unsubstituted benzophenone **1** was chosen as a model for the transformation. Computational methods are described in Supplemental Information. It was assumed that the reaction starts with the excitation of benzophenone and the subsequent intersystem crossing into the lowest lying triplet state. After considering and rejecting several direct decarbonylation pathways (see Supplemental Information), a pathway involving an oxygen transfer from a DMSO molecule to the triplet state of benzophenone (**T**_**1**_) was investigated and found to be energetically plausible (Fig. 5)^35^. The transfer of the oxygen atom from a nearby DMSO molecule to the carbonyl carbon of **T**_**1**_ is nearly thermoneutral and yields intermediate **A(T)** that is separated from reactant by a moderate 19.2 kcal/mol barrier (see Supplemental Information). In the next step, a SET from DBU to the triplet state **A(T)** occurs with the formation of radical anion **A** that lies only 3.9 kcal/mol higher in energy than A(T) and is expected to be present at sufficiently high concentration for the reaction to proceed to the stabilizing C‒C bond dissociation step. The C‒CO bond in **A** breaks with a barrier of only 6.7 kcal/mol, yielding intermediate **B** composed of two weakly bonded fragments. The second transition state (**TS2**) representing the formation of the C‒C bond between the two phenyl rings was located 14.9 kcal/mol above **B** on the pathway to form intermediate **C**. The decarboxylation step associated with the release of the weakly bound CO_2_ molecule proceeds with a small barrier relative to **C** (**TS3**, 2.7 kcal/mol) giving the radical anion product **D**, which transfers an electron back to DBU + spontaneously to produce the biphenyl product **2**. The structures of all stable intermediate and transition states are shown in Fig. 6 and Fig. 5 together with their free energies.

To summarize, DFT modeling suggests that a light-driven transformation of benzophenone to biphenyl proceeds as a low-barrier stepwise process mediated by DMSO. The energy acquired by the system after the excitation is sufficient to overcome the low-lying transition states associated with the cleavage and formation the C–C bonds.

## Discussion

In summary, we have described a light-driven transition-metal-free strategy for the decarbonylation of unstrained diaryl ketones to construct biaryl structures, via the cleavage of two C–C bonds. The reaction proceeds at room temperature, making the reaction conditions mild and easy to operate. Our current protocol can tolerate various functional-groups and can be applied to unstrained diaryl ketones and heterocyclic aryl ketones. More importantly, this one-pot protocol can even be applied to the deoxygenation of pharmaceuticals and PEEK model substrates. Further studies on the mechanism and synthetic applications of this protocol are currently underway in our laboratory.

## Methods

### General procedure for reactions in Table 1

In 15 mL quartz tube charged with a magnetic stir-bar, were added sequentially ketones (0.1 mmol, 1 equiv) and base. The tube was then evacuated and backfilled with argon three times. Additive (DBU, TMEDA, Et_3_N, DABCO), solvent (1.5 mL) and H_2_O were added by microsyringe and syringe. Then the tube was placed in a UV reactor at room temperature and the mixture was stirred for 24 h or 36 h. After completion of the reaction, the reaction mixture was cooled to room temperature, and then benzotrifluoride (6 µL, 0.05 mmol) was added into the mixture as standard. The crude mixture was diluted by CDCl_3_ to run the ^19^F NMR test to determine the ^19^F NMR yield.

### General procedure for reactions in Figs. 2, 3 and 4

In 15 mL quartz tube charged with a magnetic stir-bar, were added sequentially ketones (0.1 mmol, 1 equiv) and *t*-BuOK (0.125 mmol, 1.25 equiv). The tube was then evacuated and backfilled with argon three times. DBU (0.2 mmol, 2 equiv), DMSO (1.5 mL) and H_2_O (80 μL) were added by microsyringe and syringe. Then the tube was placed in a UV reactor at room temperature and the mixture was stirred for 36 h. 10 mL water was added to quench reaction, and the mixture was extracted with EtOAc (5 mL × 4). The combined organic solvent was washed with brine, dried with Na_2_SO_4_, and then concentrated under reduced pressure. The residues were purified by preparative TLC on silica gel eluting with hexane: EtOAc (300:1-20:1) to afford the product.

## Supplementary information

Supplementary Information

## Data Availability

The authors declare that the data supporting the findings of this study are available within the article and Supplementary Information file, or from the corresponding author upon request. Source data are provided with this paper.

## Source data

Source Data
