# Supplementary material for: Light-driven transition-metal-free direct decarbonylation of unstrained diaryl ketones via a dual C–C bond cleavage
Source: Nat Commun. 2022 Apr 4;13:1805. doi: 10.1038/s41467-022-29327-z (PMC8979990; doi:10.1038/s41467-022-29327-z)
Supplement: Supplementary file 1 — Supplementary Information [file 41467_2022_29327_MOESM1_ESM.pdf]

Supplementary Information

Light-Driven Transition-Metal-Free Direct Decarbonylation of Unstrained Diaryl Ketones via a Dual C–C Bond Cleavage

Dawei Cao, [1,2,3,4] Mohamad Ataya, [1,4] Zhangpei Chen,[1] Huiying Zeng,[3] Yong Peng,[2] Rustam Z. Khaliullin[1,*] and Chao-Jun Li[1,*]

[1] Department of Chemistry, and FQRNT Centre for Green Chemistry and Catalysis, McGill University, 801 Sherbrooke St. West, Montreal, QC H3A 0B8, Canada.

[2] Key Laboratory of Magnetism and Magnetic Materials of the Ministry of Education, Lanzhou University, Lanzhou 730000, P. R. China.

[3] The State Key Laboratory of Applied Organic Chemistry, Lanzhou University, Lanzhou 730000, P. R. China.

[4]These authors contributed equally: Dawei Cao, Mohamad Ataya.

[*]Corresponding Authors: rustam.khaliullin@mcgill.ca; cj.li@mcgill.ca

Table of Contents

Ⅰ. **Supplementary Note** **S2**

Ⅱ. **Supplementary Methods** **S2**

Ⅲ. **Supplementary Discussion** **S2**

i. Trace transition metal analysis/exclusion **S2**

ii. Procedure for gram scale reaction **S3**

iii. ^{18}O-labelling experiments **S4**

iv. CO_2, DMS and CO detection **S6**

v. UV-vis and fluorescence quenching **S8**

vi. Cyclic Voltammetry **S9**

vii. Density functional theory (DFT) calculation **S10**

viii. Characterization of products **S14**

Ⅳ. **Supplementary Figures** **S25**

Ⅴ. **Supplementary References** **S68**

Ⅰ. Supplementary Note

General Information: All reagents and solvents were purchased from commercial sources (Alfa, Acros, Aldrich, TCI and Combi-Blocks) and used without further purification unless otherwise stated. ^1H and ^{13}C NMR spectra were taken on Agilent 600, Bruker 400 or 500 MHz spectrometer. Chemical shifts of ^1H NMR spectra were reported using either residual solvent signal of $CDCl_3$ (δ = 7.26 ppm) or TMS (δ = 0.00 ppm) as internal standard. Chemical shifts of ^{13}C NMR spectra were reported using residual solvent signal of $CDCl_3$ (δ = 77.16 ppm) as internal standard. The peak patterns are indicated as follows: s, singlet; d, doublet; dd, doublet of doublet; t, triplet; q, quartet; m, multiplet. The coupling constants, *J*, are reported in Hertz (Hz). All reactions were monitored by thin-layer chromatography (TLC). Column chromatography was performed on silica gel (200-300 mesh) and visualized with ultraviolet light. EI-MS was obtained from the Agilent GC-MS system. All solvents were purified and dried by standard techniques.

Ⅱ. Supplementary Methods

General experimental procedure for decarbonylation of unstrained diaryl ketones

In a 15 mL quartz tube charged with a magnetic stir-bar, were added sequentially ketones (0.1 mmol, 1 equiv) and *t*-BuOK (14 mg, 0.125 mmol, 1.25 equiv). The tube was then evacuated and backfilled with argon three times. DBU (30 μL, 0.2 mmol, 2 equiv), DMSO (1.5 mL) and H_2O (80 μL) were added by microsyringe and syringe. Then the tube was placed in a UV reactor[1] at room temperature and the mixture was stirred for 36 h. 10 mL water was added to quench the reaction, and the mixture was extracted with EtOAc (5 mL × 4). The combined organic solvent was washed with brine, dried with Na_2SO_4, and then concentrated under reduced pressure. The residues were purified by preparative TLC on silica gel eluting with hexane : EtOAc (300:1-20:1) to afford the product.

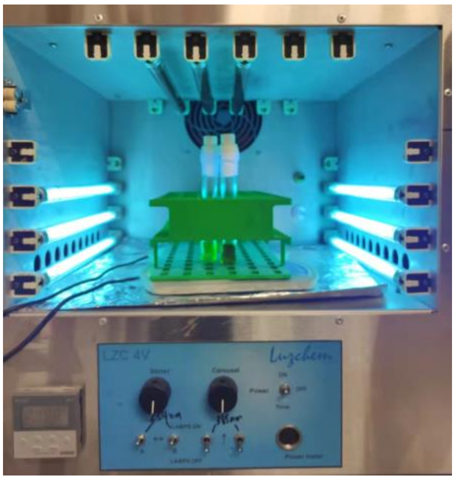

Supplementary Figure 1. UV reactor

Ⅲ. Supplementary Discussion

i. Trace transition metal analysis/exclusion

In order to rule out the possibility of a trace amount of transition metal in the reaction mixture, three different strategies have been performed:

a) 99.99% purity of *t*-BuOK and >99.0% purity of DBU were used in the standard conditions, and the reaction results remain basically unchanged.

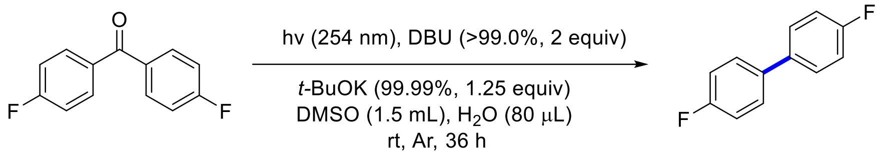

1u 0.1 mmol **2u** 72%

b) ICP-MS was used to analyze trace metal in DBU and *t*-BuOK, and the results are listed below: *t*-BuOK (84.2 mg, 0.8 mmol) and DBU (119.5 μL, 0.8 mmol) include 9Be (0.002 μg), 24Mg (0.1 ug), 27Al (0.07 μg), 40Ca (1.1 μg), 51V (0.06 μg), 55Mn (0.03 μg), 108Ag (0.9 μg), 208Pb (0.06 μg).

c) Metal influence experiments

In a 15 mL quartz tube charged with a magnetic stir-bar, were added sequentially bis(4-fluorophenyl)methanone (21.8 mg, 0.1 mmol, 1 equiv), metal (5 mol%) and *t*-BuOK (14 mg, 0.125 mmol, 1.25 equiv). The tube was then evacuated and backfilled with argon three times. DBU (30 μL, 0.2 mmol, 2 equiv), DMSO (1.5 mL) and H_2O (80 μL) were added by microsyringe and syringe. Then the tube was placed in a UV reactor at room temperature and the mixture was stirred for 36 h. The results showed that all reactions with metal additives were not as efficient as those without metal.

Supplementary Table 1. Metal influence experiments[a]

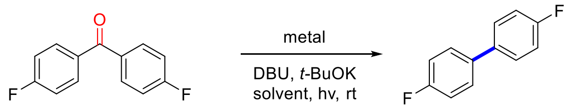

Entry	Metal	Yield[b](%)
1	$Pd(OAc)_2$	12
2	$Ni(cod)_2$	27
3	CuCl	31
4	$FeCl_2$	38
5	$CoCl_2$	16
6	$(CO)_2Rh(acac)$	28
7	$Ru(bpy)_3Cl_2•6H_2O$	44
8	$Ir(ppy)_2(bpy)PF_6$	39
9	without metal	73

[a]General conditions: **1u** (0.1 mmol), metal (5 mol%), DBU (2 equiv), *t*-BuOK (1.25 equiv) in DMSO (1.5 mL), H_2O (80 μL) at rt for 36 h under Ar. [b]Yields were determined by ^{19}F NMR with benzotrifluoride as internal standard.

The above three experimental results show that this is a transition metal-free strategy and *t*-BuOK /DBU combination plays a very important role in the reaction process.

ii. Procedure for gram scale reaction

In a 100 mL quartz round bottom flask charged with a magnetic stir-bar, were added sequentially bis(4-fluorophenyl)methanone (1.31 g, 6 mmol, 1 equiv) and *t*-BuOK (842 mg, 7.5 mmol, 1.25 equiv). The tube was then evacuated and backfilled with argon three times. DBU (1.8 mL, 12 mmol, 2 equiv), DMSO (60 mL) and H_2O (2 mL) were added by syringe. Then the flask was placed in a UV reactor at

room temperature and the mixture was stirred for 72 h. 20 mL water was added to quench the reaction, and the mixture was extracted with EtOAc (15 mL × 4). The combined organic solvent was washed with brine, dried with Na_2SO_4, and then concentrated under reduced pressure. The residues were purified by column chromatography on silica gel (eluent: hexanes : EtOAc = 50:1) to afford the 4,4'-difluoro-1,1'-biphenyl product (0.51 g, 45% yield).

iii. ^{18}O-Labelling experiments

a) Preparation of DMS^{18}O [2]

1) Preparation of bromodimethylsulfonium bromide (BDMS):

In a round-bottom flask charged with a magnetic stir-bar, bromine (6.7 mL, 130 mmol) was added dropwise to an ice-cooled solution of dimethyl sulfide (9.5 mL, 130 mmol) in 120 mL carbon tetrachloride and the mixture was continuously stirred for 2 h. The yellowish orange crystals were formed which were filtered out and washed with cold carbon tetrachloride. The solid (27 g) was recrystallized from carbon tetrachloride, and BDMS was obtained as yellow solid.

2) Preparation of DMS^{18}O:

In a round-bottom flask charged with a magnetic stir-bar, were added dry triethylamine (25 mL, 180 mmol), ^{18}O-labelled water (97 atom % ^{18}O) (0.8 mL, 44 mmol) and dry tetrahydrofuran (60 mL) at 0 °C, and then solid BDMS (20.0 g, 90 mmol) was added portion-wise over 30 min. The resulting precipitate was filtered and washed with diethyl ether. The yellow filtrate was concentrated under reduced pressure. The DMS^{18}O (2.3 g, about 76 atom% ^{18}O) was obtained as brownish liquid.

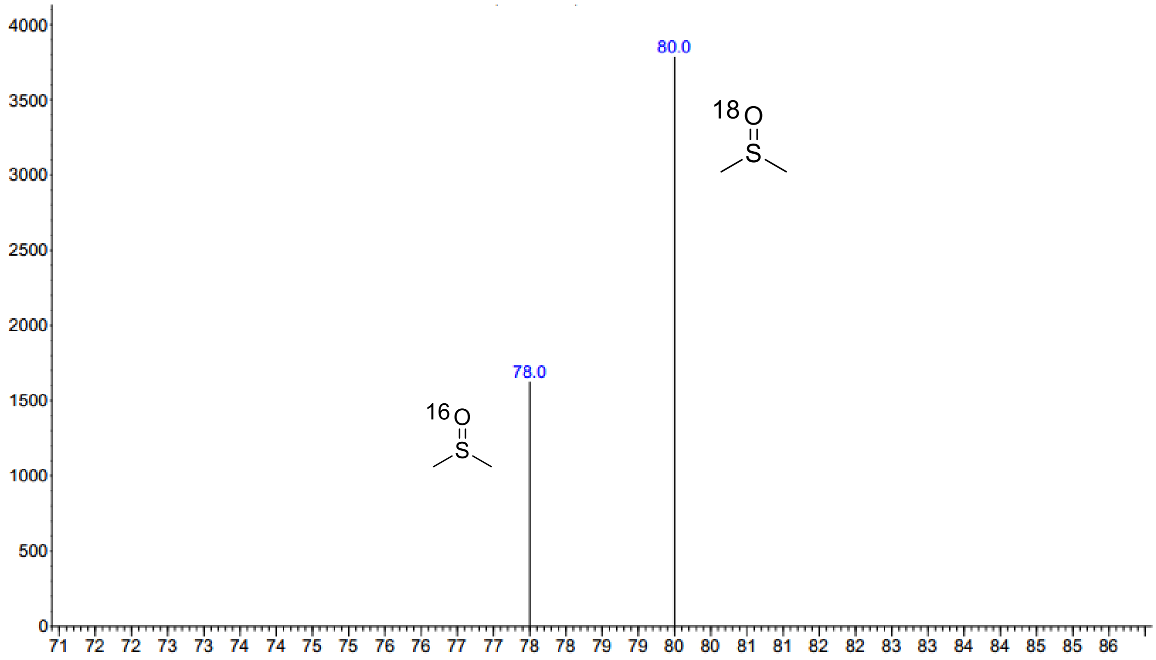

Supplementary Figure 2 GC-MS spectrum of DMS^{18}O

b) [^{18}O]–Labelling experiments with DMS^{18}O and $H_2{}^{18}O$.

1) In 15 mL quartz tube charged with a magnetic stir-bar, were added sequentially bis(4-fluorophenyl)methanone (21.8 mg, 0.1 mmol, 1 equiv) and *t*-BuOK (14 mg, 0.125 mmol, 1.25 equiv). The tube was then evacuated and backfilled with argon three times. DBU (30 µL, 0.2 mmol, 2 equiv), DMS^{16}O (1.5 mL) and $H_2{}^{16}O$ (80 µL) were added by microsyringe and syringe. Then the tube was placed in a UV reactor at room temperature and the mixture was stirred for 36 h. The gas phase inside the flange was analyzed using a valve syringe and gas chromatography mass spectrometer

(GC-MS).

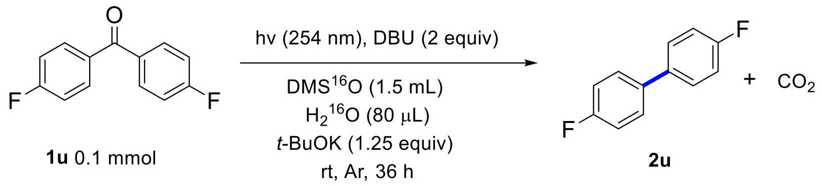

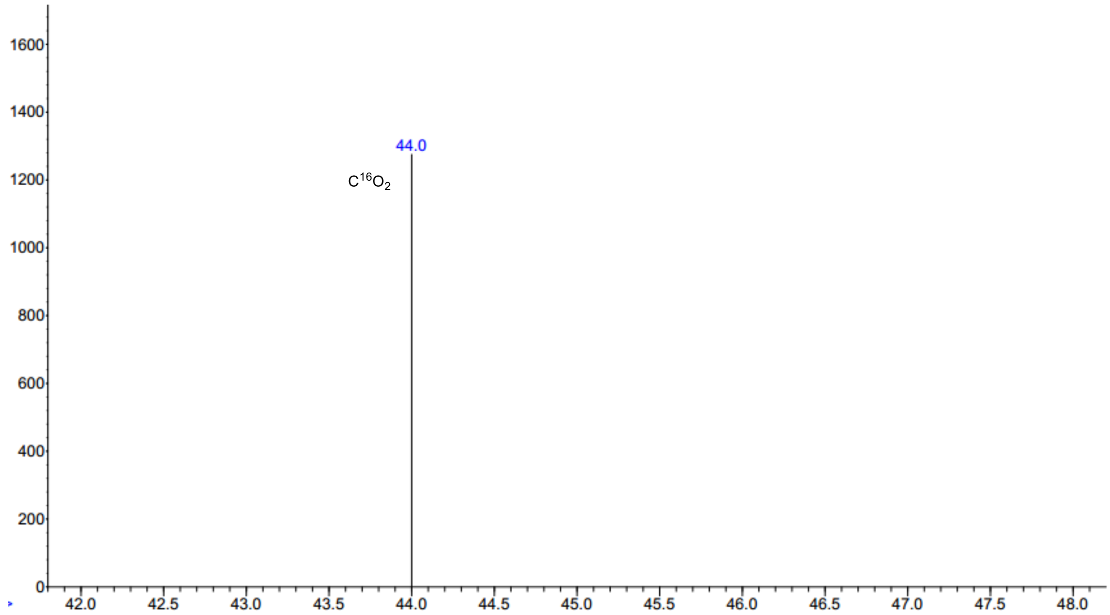

Supplementary Figure 3 MS spectrum of $C^{16}O_2$ (using $DMS^{16}O$ and $H_2{}^{16}O$).

2) In a 15 mL quartz tube charged with a magnetic stir-bar, were added sequentially bis(4-fluorophenyl)methanone (21.8 mg, 0.1 mmol, 1 equiv) and *t*-BuOK (14 mg, 0.125 mmol, 1.25 equiv). The tube was then evacuated and backfilled with argon three times. DBU (30 μL, 0.2 mmol, 2 equiv), $DMS^{16}O$ (1.5 mL) and $H_2{}^{18}O$ (80 μL) were added by microsyringe and syringe. Then the tube was placed in a UV reactor at room temperature and the mixture was stirred for 36 h. The gas phase inside the flange was analyzed using a valve syringe and gas chromatography mass spectrometer (GC-MS).

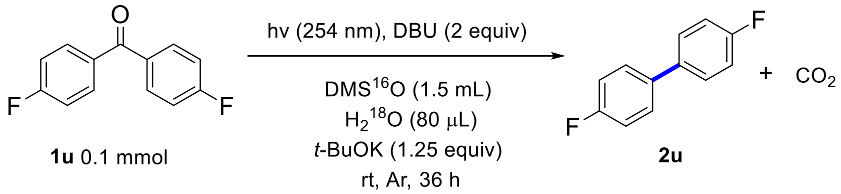

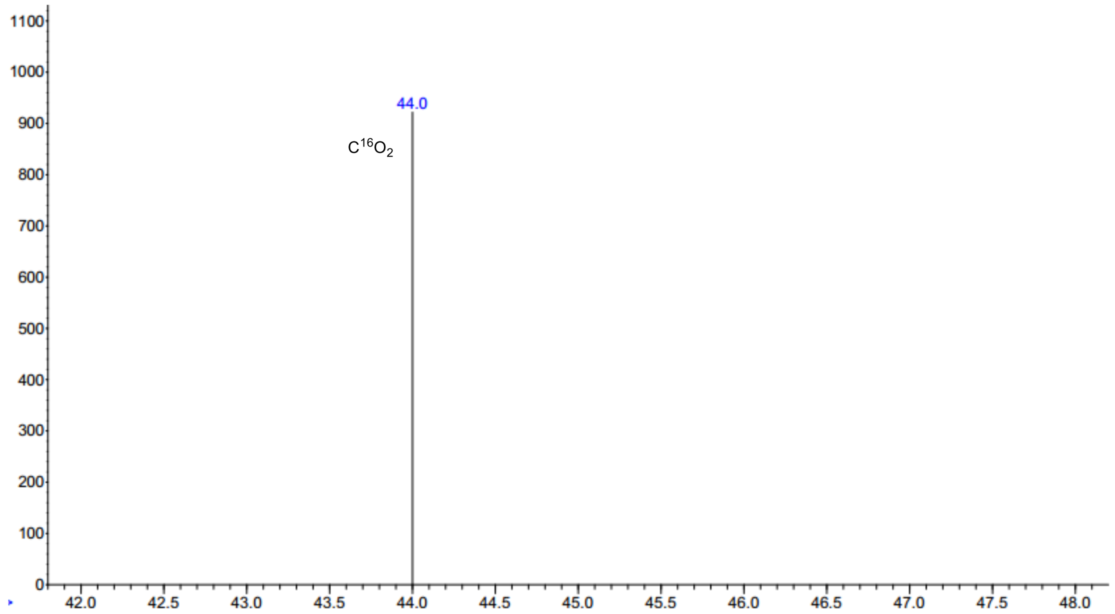

Supplementary Figure 4 MS spectrum of $C^{16}O_2$ (using $DMS^{16}O$ and $H_2{}^{18}O$).

3) In a 15 mL quartz tube charged with a magnetic stir-bar, were added sequentially bis(4-fluorophenyl)methanone (21.8 mg, 0.1 mmol, 1 equiv) and *t*-BuOK (14 mg, 0.125 mmol, 1.25 equiv). The tube was then evacuated and backfilled with argon three times. DBU (30 μL, 0.2 mmol, 2 equiv), $DMS^{18}O$ (1.5 mL) and $H_2{}^{16}O$ (80 μL) were added by microsyringe and syringe. Then the tube was placed in a UV reactor at room temperature and the mixture was stirred for 36 h. The gas phase inside the flange was analyzed using a valve syringe and gas chromatography mass spectrometer (GC-MS).

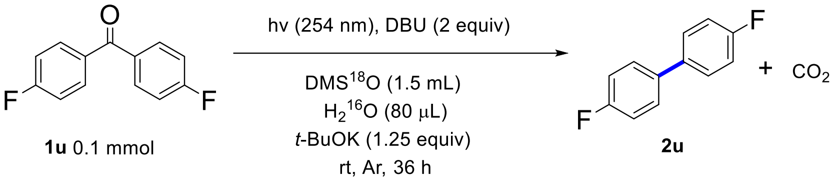

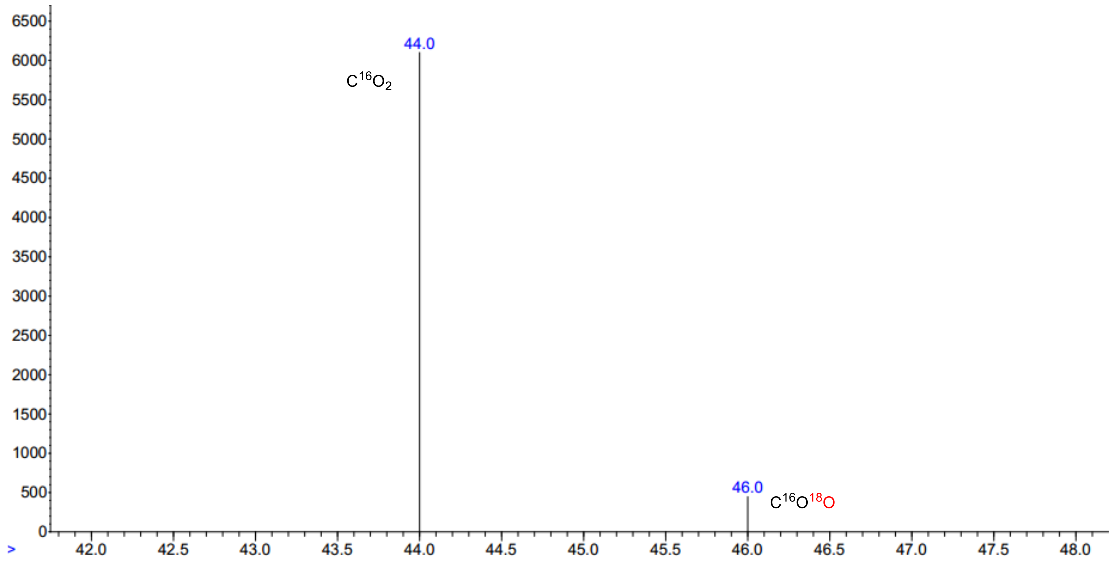

Supplementary Figure 5 MS spectrum of $C^{16}O^{18}O$ and $C^{16}O_2$ (using $DMS^{18}O$ and $H_2{}^{16}O$).

iv. CO_2, DMS and CO detection

CO_2 and DMS detection: In a 15 mL quartz tube charged with a magnetic stir-bar, were added sequentially bis(4-fluorophenyl)methanone (21.8 mg, 0.1 mmol, 1 equiv) and *t*-BuOK (14 mg, 0.125 mmol, 1.25 equiv). The tube was then evacuated and backfilled with argon three times. DBU (30 μL, 0.2 mmol, 2 equiv), DMSO (1.5 mL) and H_2O (80 μL) were added by microsyringe and syringe. Then the tube was placed in a UV reactor at room temperature and the mixture was stirred for 36 h. The gas phase inside the flange was analyzed using a valve syringe and gas chromatography mass spectrometer (GC-MS).

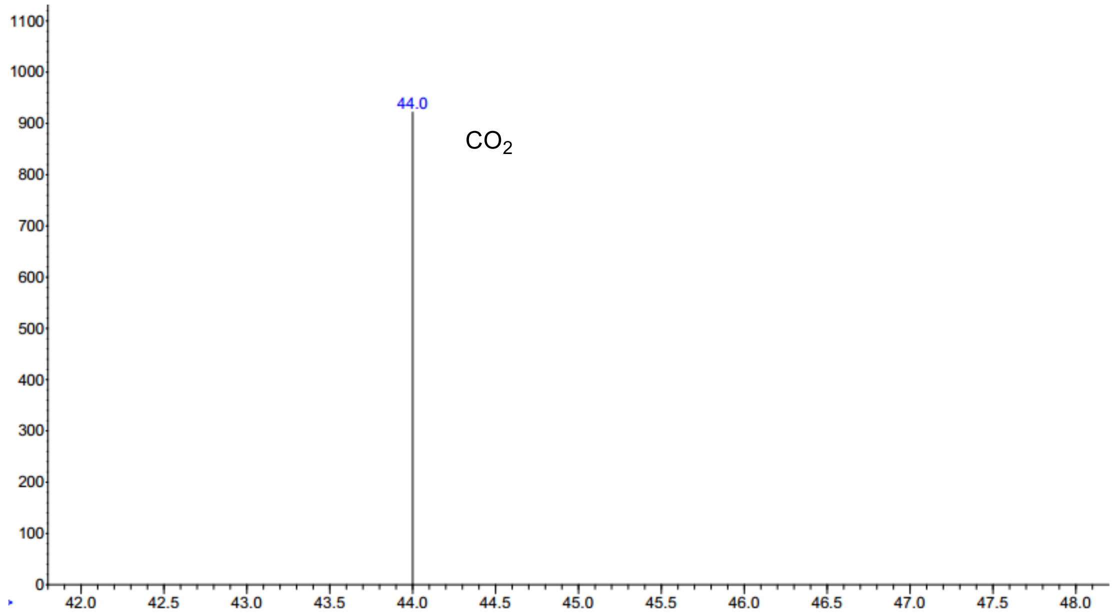

Supplementary Figure 6 MS spectrum of CO_2

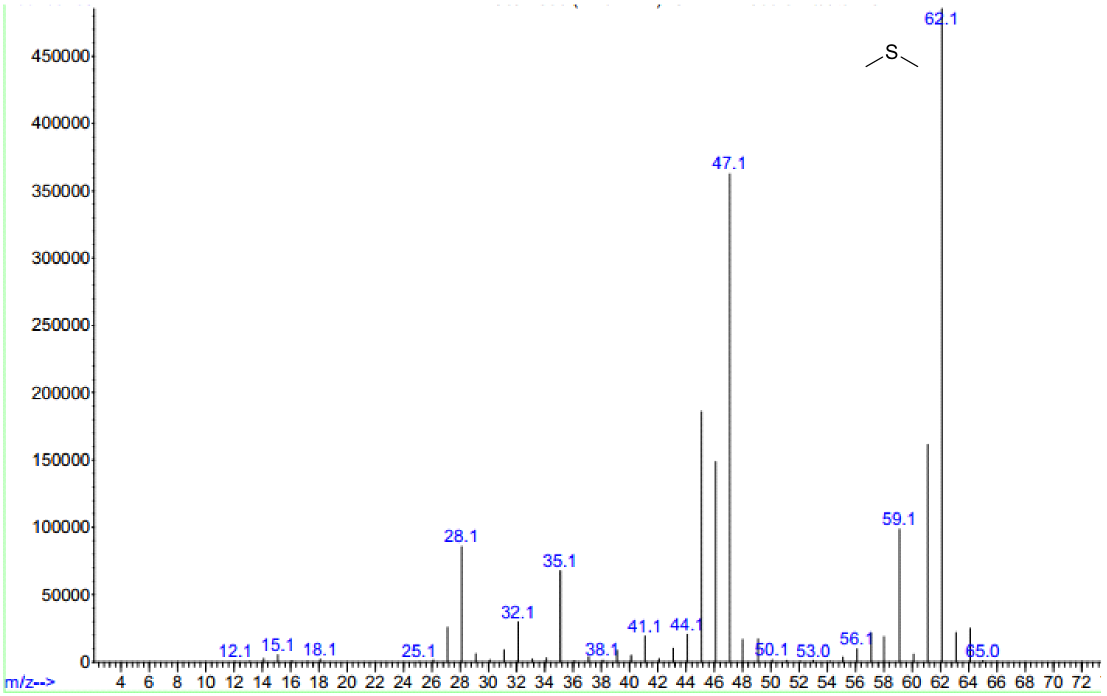

Supplementary Figure 7 MS spectrum of DMS

CO detection: In a 15 mL quartz tube charged with a magnetic stir-bar, were added sequentially bis(4-fluorophenyl)methanone (21.8 mg, 0.1 mmol, 1 equiv) and *t*-BuOK (14 mg, 0.125 mmol, 1.25 equiv). The tube was then evacuated and backfilled with argon three times. DBU (30 μL, 0.2 mmol, 2

equiv), DMSO (1.5 mL) and H_2O (80 μL) were added by microsyringe and syringe. Then the tube was placed in a UV reactor at room temperature and the mixture was stirred for 36 h. The gas phase inside the flange was analyzed using a valve syringe and gas chromatography mass spectrometer (GC-MS). The results show that there is no CO in the gas phase. We further used the EL-USB-CO detector to detect the trace amount of CO (2:00-7:00 pm continuous testing for 5 h), and also no CO was detected.

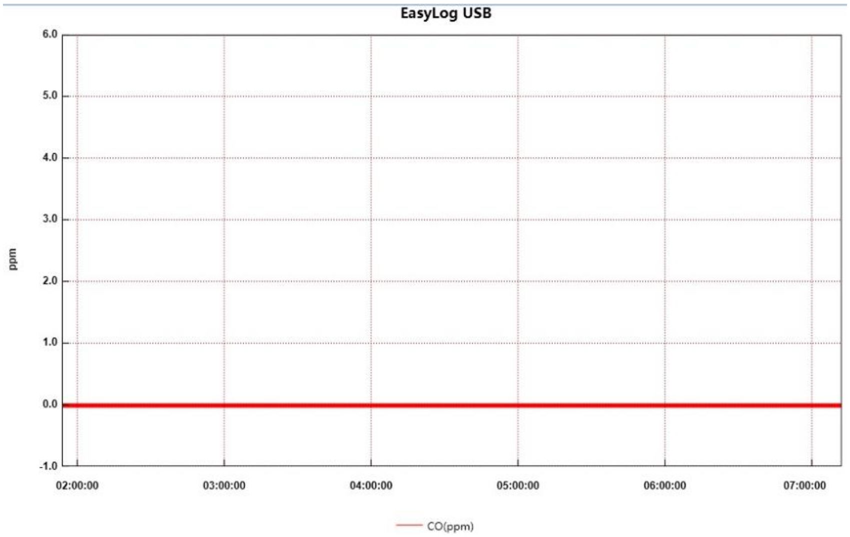

Supplementary Figure 8 The amount of CO (ppm)

v. UV-vis and fluorescence quenching

UV-vis spectrometric experiments. A stock solution of benzophenone (10 mM in HPLC grade DMSO) was prepared in a volumetric flask and diluted by HPLC grade DMSO to 0.10 mM for the UV-Vis experiment. A quartz cuvette (1 cm x 1 cm x 3 cm) was filled with the above-mentioned 0.10 mM benzophenone solution and its spectrum was recorded from 220 nm to 600 nm in the spectrometer and UV-Vis spectrum.

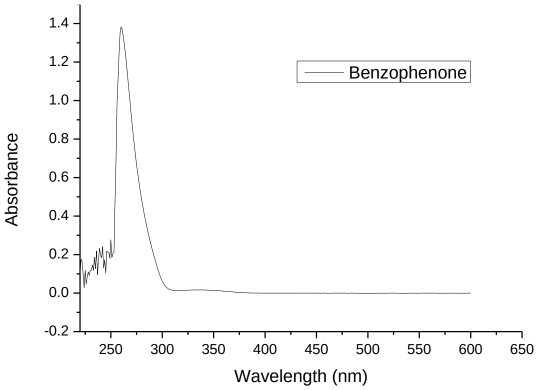

Supplementary Figure 9 UV-Vis spectrum of 0.1 mM benzophenone in DMSO.

Fluorescence quenching experiments of benzophenone. A stock solution of benzophenone (0.1 mM, in HPLC grade DMSO) was prepared in a volumetric flask and diluted by HPLC grade DMSO to 1 μM for the quenching experiment. A quartz cuvette (1 cm × 1 cm × 3 cm) was filled with the abovementioned 1 μM benzophenone solution, which was then irradiated at 254 nm. Quenching experiments were performed under duplicate conditions with the injection of 5 μL, 10 μL, 15 μL and 20 μL DBU, respectively by auto-pipette. The resulting fluorescence emission spectra are shown in Supplementary Figure 10.

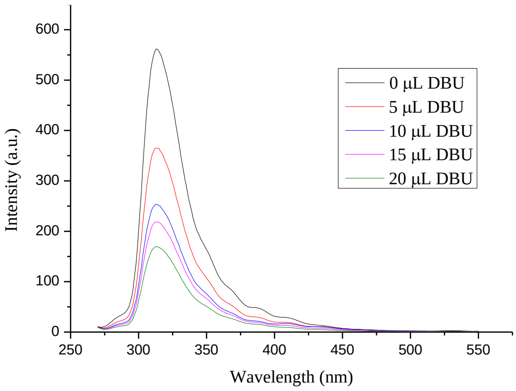

Supplementary Figure 10 Fluorescence of benzophenone quenched by DBU.

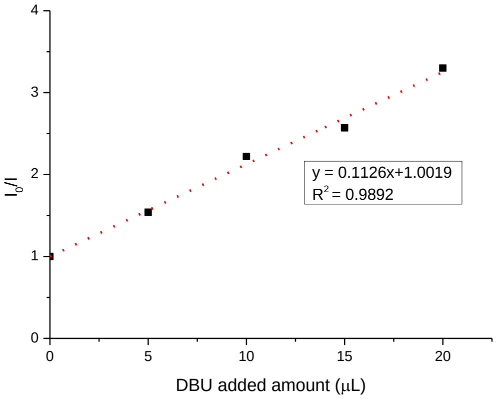

Supplementary Figure 11 Stern-Volmer plot of fluorescence of benzophenone quenched DBU.

vi. Cyclic Voltammetry

The cyclic voltammetry (CV) measurements were conducted with CHI660E electrochemical workstation, with glassy carbon (3 mm diameter) as working electrode, platinum foil (1 x 1 cm^2) as counter electrode and $Ag/AgNO_3$ as quasi-reference electrode. The measurements were done in acetonitrile with 0.1 M $^{n}Bu_4NPF_6$ as supporting electrolyte and the potentials were referenced against ferrocenium/ferrocene (Fc^+/Fc). The concentration of analyte was 0.1 M and the scan rate was at 100 mV/s. Before each test, the solution was degassed with Ar for 15 min.

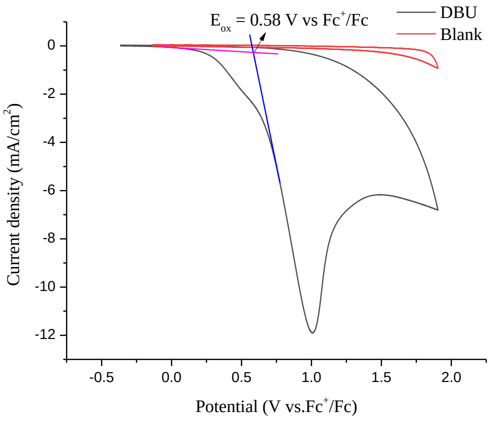

Supplementary Figure 12. Cyclic voltammetry of 0.1 M DBU in acetonitrile. 0.1 M $^{n}Bu_4NPF_6$ as supporting electrolyte and scan rate at 100 mV/s.

To account for the excited-state energy of the photocatalyst, the Gibbs free energy (ΔG_{eT}) can be

calculated using the Rehm−Weller equation: where E^{ox} (D) is the oxidation potential of the donor molecule, E^{red} (A) is the reduction potential of the acceptor molecule, E^{*} (D or A) is the singlet or triplet excited-state energy of the excited donor or acceptor molecule and $\Delta E_{Coulombic}$ (our calculation process can be ignored) is a measure of the interaction between charged ions in the dielectric constant of the solvent in which the reaction is performed.

$\Delta G_{eT} = E^{ox}$ (D) – E^{red} (A) – E^{*}(D or A) + $\Delta E_{coulombic}$

= E^{ox} (DBU) – [E^{red} (benzophenone) + E^{*}(benzophenone)]

= E^{ox} (DBU) – E^{red} (benzophenone*/ benzophenone$^{\bullet -}$) [3]

= 0.58 V vs. Fc^{+}/Fc [4] – 1.55 V vs. SCE

= 0.96 V vs. SCE – 1.55 V vs. SCE

= -0.59 V

vii. Density functional theory (DFT) calculation

All calculations were performed using the Gaussian software package (version 16, RevisionB.01). The B3LYP approximation[5] was used as the exchange-correlation functional. The 6-31++G(d,p) basis set was employed to represent spin-polarized molecular orbitals. Excited state calculations were performed using time-dependent DFT (TD-DFT). Dimethyl-sulfoxide solvent was represented implicitly in all calculations using the polarizable continuum model in the integral equation formalism.[6] Structures of stable intermediates and transition states were optimized, frequency calculations were carried out and thermodynamic functions were calculated for the standard state temperature 298.15 K and concentration 1 mol/L for all species in the ground electronic states. IRC calculations were performed to ensure that the transition states connect correct reaction intermediates.

It was assumed that the reaction starts with the excitation of benzophenone and the subsequent intersystem crossing into the lowest lying triplet state. The UV-vis absorption peak for benzophenone is calculated to lie at 277 nm (4.48 eV), in agreement with the experimentally observed transition.[7] The lowest triple state of benzophenone is calculated to lie 64 kcal/mol above the singlet ground state and is known to become populated with nearly 100% quantum yield as an excited benzophenone molecule undergoes vibrationally assisted intersystem crossing.[8]

Although there is no direct experimental evidence on the further transformation steps of the triplet state of benzophenone, the list of required reagents suggests that single electron transfer (SET) is one of the key steps in the process. The list of other possible steps includes (but is not limited to) the cleavage of the two phenyl-carbonyl bonds, formation of the carbon-carbon bond, SET from one of the intermediates back to DBU+, and possible (but not required) intersystem crossing back into the singlet state. All reasonable sequences of the elementary steps starting from the triplet state of benzophenone were considered.

Initially, the focus was on the mechanism of the decarbonylation, which results in the release of CO, not CO_2 gas. Two main hypothetical decarbonylation mechanisms were considered. In the hypothetical mechanism called **P1** (Supplementary Figure 13 and Supplementary Figure 14), the excitation is followed by SET, then by the rearrangement of carbon bonds and finally by the reversed SET. The calculations show that an initial single electron transfer (SET) from DBU to the triplet state of **1** occurs spontaneously, producing the radical anion **P1.A.** A thorough scan of the potential energy along the three coordinates representing the decarbonylation of **P1.A** – two phenyl-carbonyl and phenyl-phenyl carbon bonds – was able to locate only a single transition state lying 73 kcal/mol above the reactant state. The height of the barrier makes the transformation along the **P1** pathway prohibitively slow. In the second hypothetical mechanism called **P2** (Supplementary Figure 14 and Supplementary Figure 15),

it is the triplet state of the benzophenone that is assumed to undergo the rearrangement of carbon bonds with subsequent intersystem crossing to the singlet state (without the SET). While the barrier of the rate-determining step along **P2** (33 kcal/mol) is much lower than that along **P1** it is still too high to explain the experimentally observed rate. In addition to the high barriers along **P1** and **P2** pathways, the key biphenyl intermediates **D** and **P2.D** appear to lie higher in energy than the initial triplet state of benzophenone, rendering both paths problematic from the thermodynamics point of view. It should be noted that inserting the SET step into **P2** starting at **P2.C** leads again to the high lying **D** state. This comprehensive survey of hypothetical mechanisms suggests that there might be no energetically feasible pathways for the direct decarbonylation of **P1.A.** This conclusion gained further support when experimental measurements did not detect any appreciable amount of CO released from the reaction mixture.

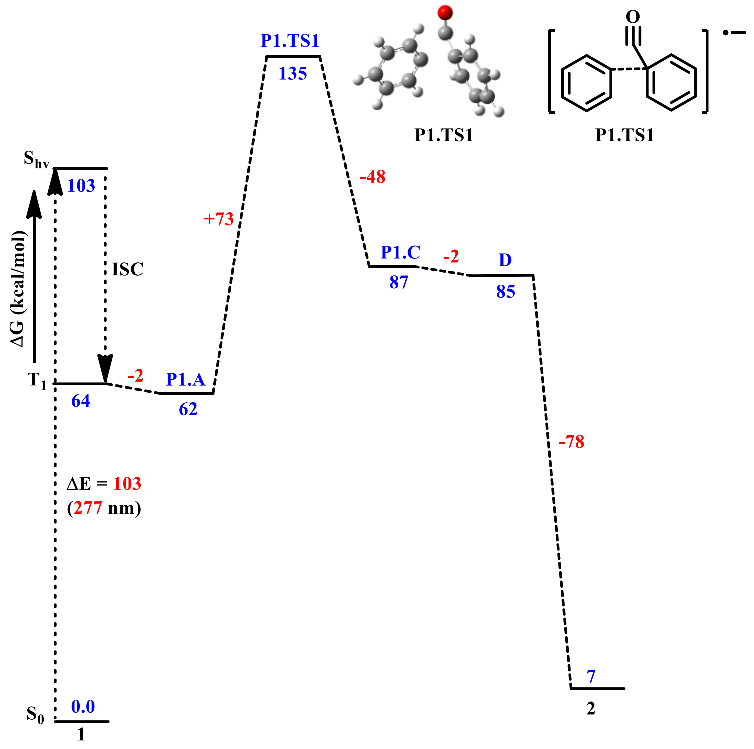

Supplementary Figure 13. B3LYP/6-31++G(d,p) free energy profile of the hypothetical **P1** pathway.

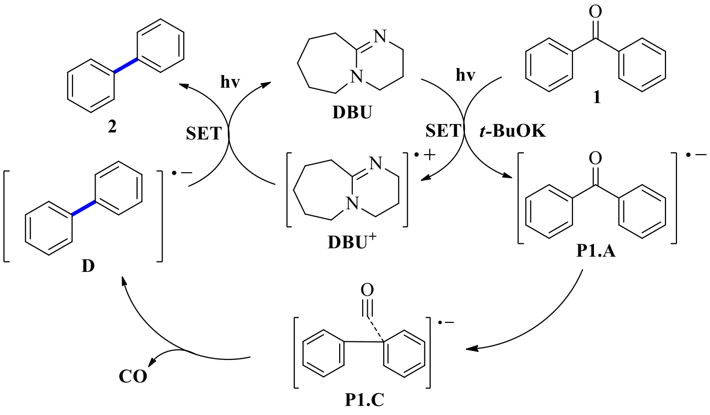

Supplementary Figure 14. Hypothetical **P1** pathway.

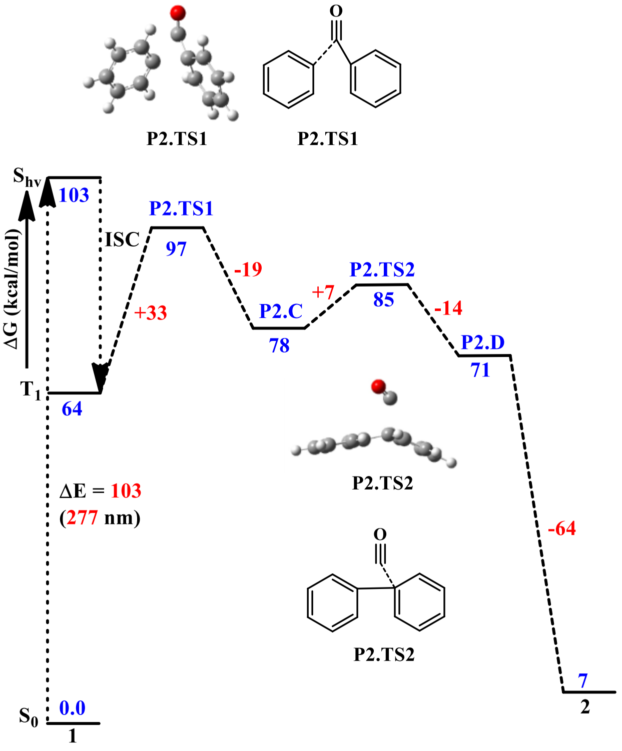

Supplementary Figure 15. B3LYP/6-31++G(d,p) free energy profile of the hypothetical **P2** pathway.

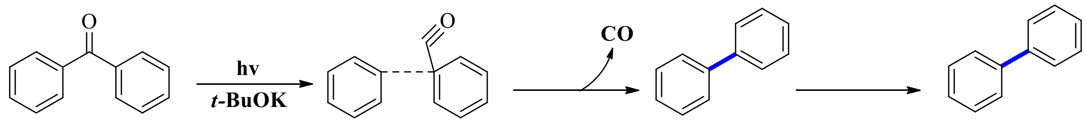

Supplementary Figure 16. Hypothetical **P2** pathway.

The possible mechanism of CO_2 release is shown in Figure 4 and Figure 5 in the manuscript. The stable species are shown in Supplementary Table 2 together with their electronic energy and free energies. Conformational search was conducted for intermediates that have conformational flexibility. Some structures along the pathway, however, are not sufficiently flexible to have several stable conformations. For example, while structure **1** has two single C–C bonds rotation around them results in the loss of the conjugation of the two aromatic π-systems or unfavorable interactions between the two phenyl rings. For intermediates **B** and **C**, the torsional angle around the broken and reformed C–C bond is an important coordinate of the reaction pathway and, cannot be used in conformational search. The conformers of products **2** and intermediates **A** and **D** have the same energy because of their symmetry. Several conformers for intermediate **A(T)** were found (Supplementary Figure 17), the lowest of which is connected to reactant **T1** by IRC going through transition state **TS0** (Figure 5).

Archive file “Cartesian-Coordinates.zip” contains the Cartesian coordinates for all computed species. The archived Gaussian output for IRC of the oxygen transfer step “RS0-IRC.zip” is also attached as Supplementary Information.

Supplementary Table 2 Stable types and related energies

Species	Electronic Energy	Electronic Energy + Thermal Free Energy a.u.
1	-576.685205146	-576.532131
S_{hv}	-576.521020235	-
T_1	-576.580232380	-576.430702
TS0	-1129.78870000	-1129.5716000
A(T)	-651.77448	-651.62331
DBU	-462.139410000	-461.929550
DBU^+	-461.946741713	-461.737903
DMSO	-553.222517872	-553.171468
DMS	-478.030071374	-477.981271
A	-651.960443163	-651.808751
TS1	-651.946710000	-651.798066
B	-651.978637500	-651.839012
TS2	-651.963541626	-651.815341
C	-651.998666421	-651.848415
TS3	-651.993989592	-651.844189
D	-463.40996000	-463.270337
CO_2	-188.592461050	-188.601724
2	-463.349372145	-463.202722
P1.A	-576.77436	-576.62471
P1.TS1	-576.65207	-576.50808
P1.C	-576.72645	-576.586
P2.TS1	-576.52377	-576.378
P2.C	-576.55546	-576.408
P2.TS2	-576.53944	-576.396
P2.D	-576.55419	-576.419

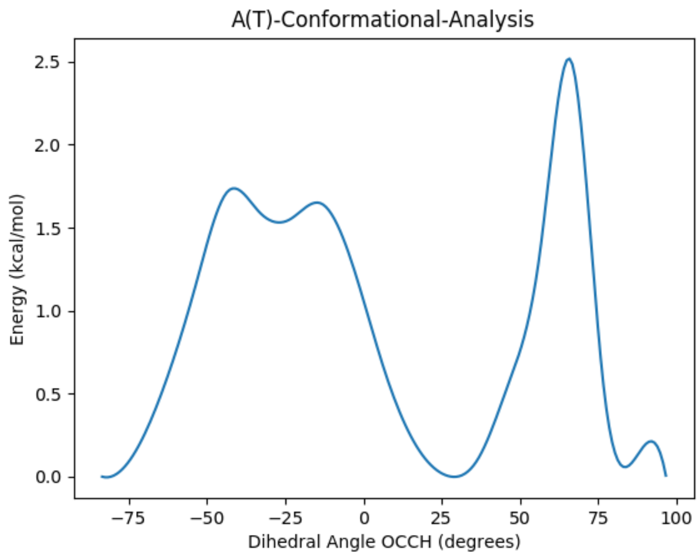

Supplementary Figure 17. Conformational analysis for intermediate **A(T)**.

viii. Characterization of products

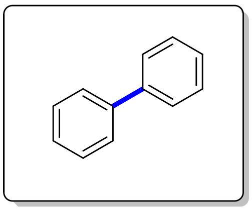

1,1'-Biphenyl (CAS: 92-52-4)[9]

^{1}H NMR ($CDCl_3$, 400 MHz) δ: 7.62 (d, *J* = 8 Hz, 4H), 7.46 (d, *J* = 8 Hz, 4H), 7.38 (t, *J* = 8 Hz, 2H).

^{13}C NMR ($CDCl_3$, 101 MHz) δ: 141.3, 128.7, 127.2 (2C).

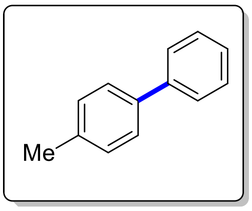

4-Methyl-1,1'-biphenyl (CAS: 644-08-6)[10]

^{1}H NMR ($CDCl_3$, 600 MHz) δ: 7.61 (d, *J* = 7.8 Hz, 2H), 7.53 (d, *J* = 5.5 Hz, 2H), 7.45 (t, *J* = 6.6 Hz, 2H), 7.37 – 7.33 (m, 1H), 7.28 (d, *J* = 6.2 Hz, 2H), 2.43 (s, 3H).

^{13}C NMR ($CDCl_3$, 151 MHz) δ: 141.2, 138.4, 137.0, 129.5, 128.7, 127.0 (2C), 126.9, 21.1.

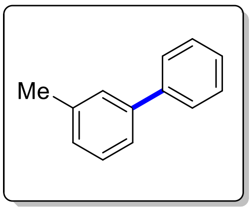

3-Methyl-1,1'-biphenyl (CAS: 643-93-6)[10]

^{1}H NMR ($CDCl_3$, 600 MHz) δ: 7.63 – 7.60 (m, 2H), 7.47 – 7.41 (m, 4H), 7.36 (dd, *J* = 12.2, 4.4 Hz, 2H), 7.19 (d, *J* = 7.5 Hz, 1H), 2.45 (s, 3H).

^{13}C NMR ($CDCl_3$, 151 MHz) δ: 141.4, 141.3, 138.3, 128.7, 128.6, 128.0 (2C), 127.2, 127.1, 124.3, 21.5.

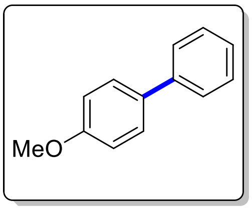

4-Methoxy-1,1'-biphenyl (CAS: 613-37-6)[10]

^{1}H NMR ($CDCl_3$, 600 MHz) δ: 7.56 (ddd, *J* = 9.5, 8.1, 0.9 Hz, 4H), 7.43 (dd, *J* = 11.5, 4.0 Hz, 2H), 7.32 (td, *J* = 7.6, 1.1 Hz, 1H), 7.02 – 6.98 (m, 2H), 3.86 (s, 3H)

^{13}C NMR ($CDCl_3$, 151 MHz) δ: 159.1, 140.8, 133.8, 128.7, 128.1, 126.7, 126.6, 114.2, 55.3.

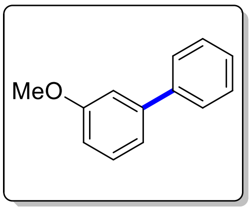

3-Methoxy-1,1'-biphenyl (CAS: 2113-56-6)[10]

^{1}H NMR ($CDCl_3$, 400 MHz) δ: 7.63 (d, *J* = 7.3 Hz, 2H), 7.48 (t, *J* = 7.5 Hz, 2H), 7.40 (tt, *J* = 7.2, 3.5 Hz, 2H), 7.23 (d, *J* = 7.6 Hz, 1H), 7.17 (s, 1H), 6.94 (dd, *J* = 8.2, 1.7 Hz, 1H), 3.91 (s, 3H).

^{13}C NMR ($CDCl_3$, 101 MHz) δ: 159.9, 142.9, 141.1, 129.7, 128.7, 127.4, 127.2, 119.7, 112.9, 112.7, 55.3.

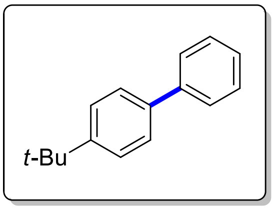

4-(Tert-butyl)-1,1'-biphenyl (CAS: 1625-92-9)[10]

^{1}H NMR ($CDCl_3$, 600 MHz) δ: 7.66 – 7.62 (m, 2H), 7.61 – 7.57 (m, 2H), 7.54 – 7.49 (m, 2H), 7.47 (td, *J* = 7.7, 1.8 Hz, 2H), 7.37 (td, *J* = 7.1, 1.2 Hz, 1H), 1.43 – 1.41 (m, 9H)

^{13}C NMR ($CDCl_3$, 151 MHz) δ: 150.2, 141.1, 138.3, 128.7, 127.0 (2C), 126.8, 125.7, 34.5, 31.4.

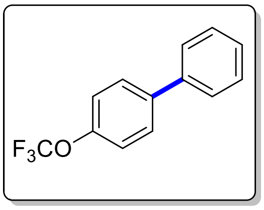

4-(Trifluoromethoxy)-1,1'-biphenyl (CAS: 71274-84-5)[11]

^{1}H NMR ($CDCl_3$, 600 MHz) δ: 7.59 (dd, *J* = 22.2, 7.9 Hz, 4H), 7.46 (t, *J* = 7.4 Hz, 2H), 7.38 (t, *J* = 7.2 Hz, 1H), 7.30 (d, *J* = 8.1 Hz, 2H).

^{13}C NMR ($CDCl_3$, 151 MHz) δ: 148.7, 140.0, 139.9, 128.9, 128.5, 127.7, 127.1, 121.2, 120.6(q, J^F = 258.2 Hz).

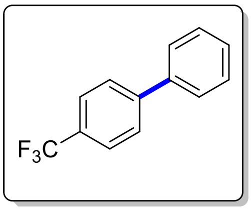

4-(Trifluoromethyl)-1,1'-biphenyl (CAS: 398-36-7)[10]

^{1}H NMR ($CDCl_3$, 600 MHz) δ: 7.71 (s, 4H), 7.64 – 7.59 (m, 2H), 7.49 (dd, *J* = 11.0, 3.8 Hz, 2H), 7.45 – 7.40 (m, 1H).

^{13}C NMR ($CDCl_3$, 151 MHz) δ: 144.8, 139.8, 129.5 (q, J^F = 33.2 Hz), 129.0, 128.2, 127.4, 127.3, 125.7 (q, J^F = 4.5 Hz), 124.3 (q, J^F = 271.8 Hz).

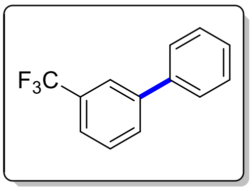

3-(Trifluoromethyl)-1,1'-biphenyl (CAS: 366-04-1)[10]

^{1}H NMR ($CDCl_3$, 400 MHz) δ: 7.91 (s, 1H), 7.82 (d, *J* = 7.6 Hz, 1H), 7.68 – 7.64 (m, 3H), 7.60 (t, *J* = 7.7 Hz, 1H), 7.54 (dd, *J* = 11.3, 4.2 Hz, 2H), 7.49 – 7.44 (m, 1H).

^{13}C NMR ($CDCl_3$, 101 MHz) δ: 142.0, 139.7, 131.1 (q, J^F = 33.3 Hz), 130.5, 129.2, 129.0, 128.0, 127.2, 124.6 (q, J^F = 271.2 Hz), 124.0 (q, J^F = 2.8 Hz), 123.9 (q, J^F = 2.6 Hz).

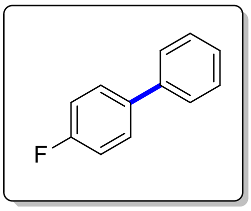

4-Fluoro-1,1'-biphenyl (CAS: 324-74-3)[10]

^{1}H NMR ($CDCl_3$, 600 MHz) δ: 7.58 – 7.53 (m, 4H), 7.47 – 7.42 (m, 2H), 7.38 – 7.34 (m, 1H), 7.16 – 7.10 (m, 2H).

^{13}C NMR ($CDCl_3$, 151 MHz) δ: 162.5 (d, J^F = 247.6 Hz), 140.3, 137.4, 128.8, 128.7 (d, J^F = 7.6 Hz), 127.2, 127.0, 115.6 (d, J^F = 21.1 Hz).

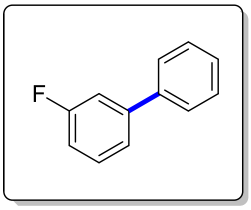

3-Fluoro-1,1'-biphenyl (CAS: 2367-22-8)[10]

^{1}H NMR ($CDCl_3$, 600 MHz) δ: 7.58 (d, *J* = 7.4 Hz, 2H), 7.45 (t, *J* = 7.6 Hz, 2H), 7.42 – 7.36 (m, 3H), 7.30 (d, *J* = 10.2 Hz, 1H), 7.04 (dd, *J* = 10.5, 5.0 Hz, 1H).

^{13}C NMR ($CDCl_3$, 151 MHz) δ: 163.2 (d, J^F = 246.1 Hz), 143.5 (d, J^F = 7.6 Hz), 140.0 (d, J^F = 3.0 Hz), 130.2 (d, J^F = 9.1 Hz), 129.0, 127.8, 127.1, 122.7 (d, J^F = 3.0 Hz), 114.1, 113.9.

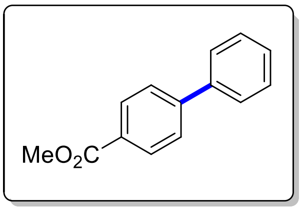

Methyl [1,1'-biphenyl]-4-carboxylate (CAS: 720-75-2)[10]

^{1}H NMR ($CDCl_3$, 600 MHz) δ: 8.13 – 8.09 (m, 2H), 7.67 – 7.65 (m, 2H), 7.64 – 7.61 (m, 2H), 7.47 (t, *J* = 7.7 Hz, 2H), 7.41 – 7.38 (m, 1H), 3.94 (s, 3H).

^{13}C NMR ($CDCl_3$, 151 MHz) δ: 167.0, 145.6, 140.0, 130.1, 128.9 (2C), 128.1, 127.2, 127.0, 52.1.

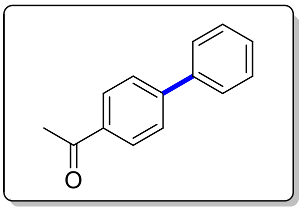

4-Acetylbiphenyl (CAS: 92-91-1)[10]

^{1}H NMR ($CDCl_3$, 400 MHz) δ: 8.09 – 8.03 (m, 2H), 7.74 – 7.69 (m, 2H), 7.68 – 7.63 (m, 2H), 7.53 – 7.47 (m, 2H), 7.46 – 7.40 (m, 1H), 2.67 (s, 3H).

^{13}C NMR ($CDCl_3$, 101 MHz) δ: 197.7, 145.7, 139.8, 135.8, 128.9, 128.9, 128.2, 127.2, 127.2, 26.6.

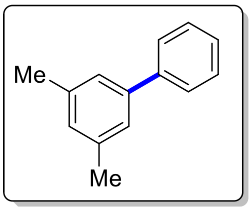

3,5-Dimethyl-1,1'-biphenyl (CAS: 17057-88-4)[12]

^{1}H NMR ($CDCl_3$, 600 MHz) δ: 7.62 – 7.59 (m, 2H), 7.45 (dd, J = 10.6, 4.8 Hz, 2H), 7.35 (dd, J = 10.6, 4.2 Hz, 1H), 7.25 (d, J = 5.4 Hz, 2H), 7.03 (s, 1H), 2.42 (s, 6H).

^{13}C NMR ($CDCl_3$, 151 MHz) δ: 141.5, 141.3, 138.2, 128.9, 128.6, 127.2, 127.0, 125.1, 21.4.

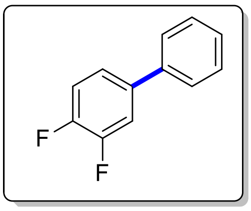

3,4-Difluoro-1,1'-biphenyl (CAS: 67277-33-2)[10]

^{1}H NMR ($CDCl_3$, 600 MHz) δ: 7.53 (d, J = 7.8 Hz, 2H), 7.45 (t, J = 7.6 Hz, 2H), 7.39 (dt, J = 14.7, 4.6 Hz, 2H), 7.32 – 7.28 (m, 1H), 7.26 – 7.20 (m, 1H).

^{13}C NMR ($CDCl_3$, 151 MHz) δ: 150.6 (dd, J^F = 247.6, 13.6 Hz), 150.0 (dd, J^F = 249.2, 12.1 Hz), 139.2, 138.4 (dd, J^F = 6.0, 4.5 Hz), 128.9, 127.8, 127.0, 123.0 (dd, J^F = 6.0, 3.0 Hz), 117.5 (d, J^F = 18.1 Hz), 116.0 (d, J^F = 18.1 Hz).

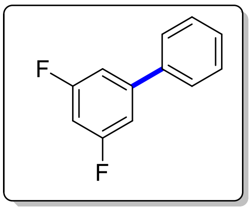

3,5-Difluoro-1,1'-biphenyl (CAS: 62351-48-8)[13]

^{1}H NMR ($CDCl_3$, 500 MHz) δ: 7.61 – 7.56 (m, 2H), 7.51 – 7.46 (m, 2H), 7.46 – 7.40 (m, 1H), 7.17 – 7.09 (m, 2H), 6.81 (tt, J = 8.9, 2.3 Hz, 1H).

^{13}C NMR ($CDCl_3$, 126 MHz) δ: 163.3 (d, J^F = 248.2 Hz), 163.2 (d, J^F = 248.2 Hz), 144.5 (t, J^F = 10.1 Hz), 138.9, 129.0, 128.4, 127.0, 109.9 (d, J^F = 25.2 Hz), 109.8 (d, J^F = 25.2 Hz), 102.5 (t, J^F = 25.2 Hz).

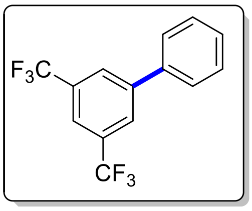

3,5-Bis(trifluoromethyl)-1,1'-biphenyl (CAS: 336621-50-2)[14]

^{1}H NMR ($CDCl_3$, 600 MHz) δ: 8.02 (s, 2H), 7.86 (s, 1H), 7.61 (dd, *J* = 7.2, 0.9 Hz, 2H), 7.51 (t, *J* = 7.8 Hz, 2H), 7.48 – 7.43 (m, 1H).

^{13}C NMR ($CDCl_3$, 151 MHz) δ: 143.4, 138.3, 132.3 (q, J^F = 33.3 Hz), 129.3, 128.9, 127.2, 127.3 – 127.1(m), 123.4 (q, J^F = 271.8.3 Hz), 120.9 (m).

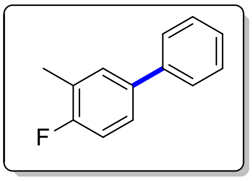

4-Fluoro-3-methyl-1,1'-biphenyl (CAS: 742086-19-7)[15]

^{1}H NMR ($CDCl_3$, 600 MHz) δ: 7.54 (d, *J* = 7.5 Hz, 2H), 7.45 – 7.39 (m, 3H), 7.36 (ddd, *J* = 19.5, 9.8, 4.9 Hz, 2H), 7.07 (t, *J* = 8.9 Hz, 1H), 2.35 (d, *J* = 1.4 Hz, 3H).

^{13}C NMR ($CDCl_3$, 151 MHz) δ: 161.1(d, J^F = 244.6 Hz), 140.5, 137.1, 130.2 (d, J^F = 4.5 Hz), 128.7, 127.1, 127.0, 125.9 (d, J^F = 7.6 Hz), 125.0 (d, J^F = 16.6 Hz), 115.2 (d, J^F = 22.7 Hz), 14.7.

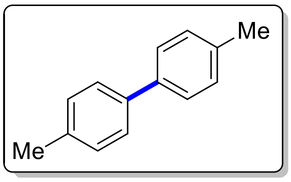

4,4'-Dimethyl-1,1'-biphenyl (CAS: 613-33-2)[16]

^{1}H NMR ($CDCl_3$, 400 MHz) δ: 7.51 (d, *J* = 8.1 Hz, 4H), 7.27 (d, *J* = 8.0 Hz, 4H), 2.42 (s, 6H).

^{13}C NMR ($CDCl_3$, 101 MHz) δ: 138.3, 136.7, 129.4, 126.8, 21.1.

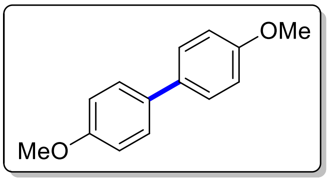

4,4'-Dimethoxy-1,1'-biphenyl (CAS: 2132-80-1)[17]

^{1}H NMR ($CDCl_3$, 400 MHz) δ: 7.53 – 7.47 (m, 4H), 7.01 – 6.95 (m, 4H), 3.87 (s, 6H).

^{13}C NMR ($CDCl_3$, 101 MHz) δ: 158.7, 133.5, 127.7, 114.2, 55.3.

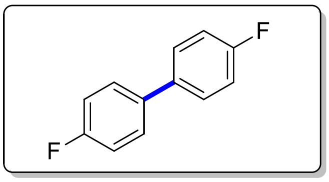

4,4'-Difluoro-1,1'-biphenyl (CAS: 398-23-2)[18]

^{1}H NMR ($CDCl_3$, 400 MHz) δ: 7.56 – 7.47 (m, 4H), 7.19 – 7.09 (m, 4H).

^{13}C NMR (CDCl$_3$, 101 MHz) δ: 162.4 (d, J^F = 247.5 Hz), 136.4 (d, J^F = 3.0 Hz), 128.6 (d, J^F = 8.1 Hz), 115.6 (d, J^F = 21.2 Hz).

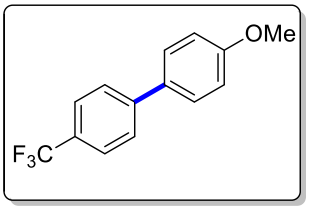

4-Methoxy-4'-(trifluoromethyl)-1,1'-biphenyl (CAS: 10355-12-1)[19]

^{1}H NMR (CDCl$_3$, 500 MHz) δ: 7.71 – 7.65 (m, 4H), 7.59 – 7.55 (m, 2H), 7.05 – 7.00 (m, 2H), 3.89 (s, 3H).

^{13}C NMR (CDCl$_3$, 126 MHz) δ: 159.8, 144.3, 132.2, 128.8 (q, J^F = 31.5 Hz), 128.4, 126.9, 125.7 (q, J^F = 3.8 Hz), 124.4 (q, J^F = 272.2 Hz), 114.4, 55.4.

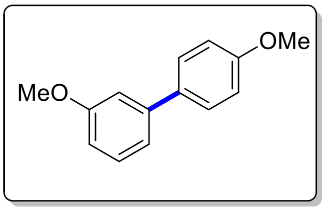

3,4'-Dimethoxy-1,1'-biphenyl (CAS: 84591-12-8)[10]

^{1}H NMR (CDCl$_3$, 500 MHz) δ: 7.61 – 7.55 (m, 2H), 7.38 (t, J = 7.9 Hz, 1H), 7.20 (d, J = 7.7 Hz, 1H), 7.16 – 7.12 (m, 1H), 7.02 (d, J = 8.6 Hz, 2H), 6.91 (dd, J = 8.2, 1.5 Hz, 1H), 3.90 (s, 3H), 3.89 (s, 3H).

^{13}C NMR (CDCl$_3$, 126 MHz) δ: 159.9, 159.2, 142.4, 133.6, 129.7, 128.2, 119.3, 114.2, 112.5, 112.0, 55.4, 55.3.

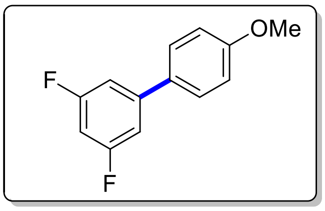

3,5-Difluoro-4'-methoxy-1,1'-biphenyl (CAS: 865856-73-1)[20]

^{1}H NMR (CDCl$_3$, 400 MHz) δ: 7.52 (d, J = 8.7 Hz, 2H), 7.11 – 7.06 (m, 2H), 7.01 (d, J = 8.7 Hz, 2H), 6.76 (ddd, J = 8.9, 5.6, 2.2 Hz, 1H), 3.89 (s, 3H).

^{13}C NMR (CDCl$_3$, 101 MHz) δ: 164.5 (d, J^F = 14.1 Hz), 162.1 (d, J^F = 13.1 Hz), 160.0, 144.1(d, J^F = 10.1 Hz), 131.3, 128.1, 114.4, 109.3 (d, J^F = 7.1 Hz), 109.2 (d, J^F = 7.1 Hz), 101.8 (t, J^F = 25.3 Hz), 55.4.

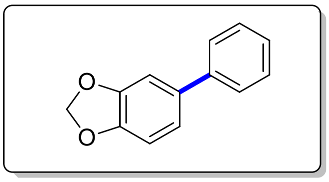

3,4-Methylenedioxybiphenyl (CAS: 24382-05-6)[10]

^{1}H NMR (CDCl$_3$, 400 MHz) δ: 7.58 (d, J = 7.5 Hz, 2H), 7.47 (t, J = 7.6 Hz, 2H), 7.37 (t, J = 7.3 Hz, 1H), 7.13 (dd, J = 10.0, 1.5 Hz, 2H), 6.94 (d, J = 7.9 Hz, 1H), 6.04 (s, 2H).

^{13}C NMR ($CDCl_3$, 101 MHz) δ: 148.1, 147.0, 140.9, 135.6, 128.7, 126.9, 126.8, 120.6, 108.5, 107.6, 101.1, 29.7.

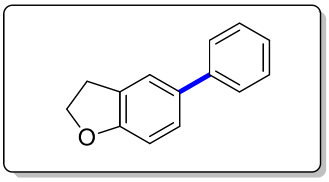

5-Phenyl-2,3-dihydrobenzofuran (CAS: 121767-87-1)[21]

^{1}H NMR ($CDCl_3$, 400 MHz) δ: 7.59 – 7.53 (m, 2H), 7.47 – 7.40 (m, 3H), 7.38 – 7.35 (m, 1H), 7.33 – 7.30 (m, 1H), 6.88 (d, *J* = 8.2 Hz, 1H), 4.64 (t, *J* = 8.7 Hz, 2H), 3.30 (t, *J* = 8.7 Hz, 2H).

^{13}C NMR ($CDCl_3$, 101 MHz) δ: 159.7, 141.3, 134.0, 128.7, 127.6, 127.1, 126.8, 126.5, 123.8, 109.4, 71.5, 29.8.

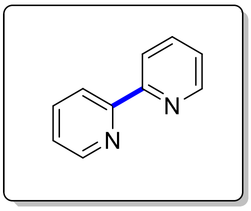

2,2'-Bipyridine (CAS: 366-18-7)[22]

^{1}H NMR ($CDCl_3$, 400 MHz) δ: 8.71 (d, *J* = 4.7 Hz, 2H), 8.42 (d, *J* = 8.0 Hz, 2H), 7.84 (td, *J* = 7.9, 1.8 Hz, 2H), 7.33 (ddd, *J* = 7.4, 4.8, 1.1 Hz, 2H).

^{13}C NMR ($CDCl_3$, 101 MHz) δ: 156.1, 149.2, 136.9, 123.7, 121.1.

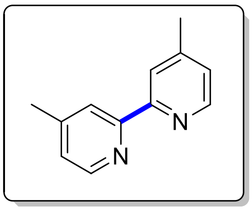

4,4'-Dimethyl-2,2'-bipyridine (CAS: 1134-35-6)[22]

^{1}H NMR ($CDCl_3$, 400 MHz) δ: 8.55 (d, *J* = 4.9 Hz, 2H), 8.24 (s, 2H), 7.15 (d, *J* = 4.8 Hz, 2H), 2.46 (s, 6H).

^{13}C NMR ($CDCl_3$, 101 MHz) δ: 156.0, 148.9, 148.1, 124.6, 122.0, 21.2.

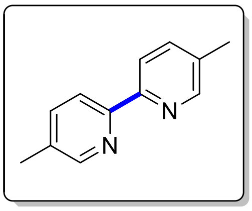

5,5'-Dimethyl-2,2'-bipyridine (CAS: 1762-34-1)[22]

^{1}H NMR ($CDCl_3$, 400 MHz) δ: 8.51 (s, 2H), 8.26 (d, *J* = 8.1 Hz, 2H), 7.63 (d, *J* = 8.1 Hz, 2H), 2.40 (s, 6H).

^{13}C NMR ($CDCl_3$, 101 MHz) δ: 153.8, 149.5, 137.4, 133.0, 120.3, 18.3.

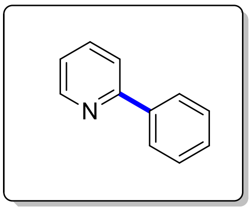

2-Phenylpyridine (CAS: 1008-89-5)[23]

^{1}H NMR ($CDCl_3$, 400 MHz) δ: 8.75 – 8.71 (m, 1H), 8.05 – 8.00 (m, 2H), 7.80 – 7.74 (m, 2H), 7.53 – 7.48 (m, 2H), 7.47 – 7.41 (m, 1H), 7.26 (ddd, *J* = 6.8, 4.8, 2.2 Hz, 1H).

^{13}C NMR ($CDCl_3$, 101 MHz) δ: 157.5, 149.7, 139.4, 136.7, 128.9, 128.7, 126.9, 122.1, 120.5.

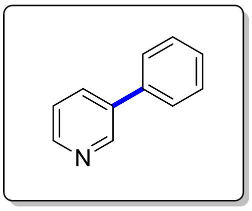

3-Phenylpyridine (CAS: 1008-88-4)[23]

^{1}H NMR ($CDCl_3$, 400 MHz) δ: 8.86 (s, 1H), 8.60 (d, *J* = 4.7 Hz, 1H), 7.91 – 7.85 (m, 1H), 7.59 (d, *J* = 8.0 Hz, 2H), 7.49 (dd, *J* = 8.1, 6.8 Hz, 2H), 7.40 (ddd, *J* = 12.8, 6.5, 3.1 Hz, 2H).

^{13}C NMR ($CDCl_3$, 101 MHz) δ: 148.3, 148.2, 137.7, 136.5, 134.2, 129.0, 128.0, 127.0, 123.4.

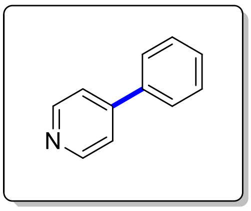

4-Phenylpyridine (CAS: 939-23-1)[23]

^{1}H NMR ($CDCl_3$, 600 MHz) δ: 8.66 (dd, *J* = 4.5, 1.6 Hz, 2H), 7.66 – 7.62 (m, 2H), 7.52 – 7.47 (m, 4H), 7.45 (dd, J = 4.9, 3.7 Hz, 1H).

^{13}C NMR ($CDCl_3$, 151 MHz) δ: 150.2, 148.4, 138.2, 129.1 (2C), 127.0, 121.7.

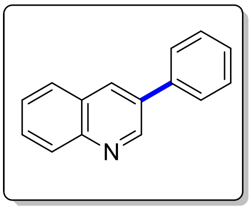

3-Phenylquinoline (CAS: 1666-96-2)[23]

^{1}H NMR ($CDCl_3$, 400 MHz) δ: 9.22 (d, *J* = 2.3 Hz, 1H), 8.33 (d, *J* = 2.1 Hz, 1H), 8.18 (d, *J* = 8.5 Hz, 1H), 7.91 (dd, *J* = 8.1, 1.2 Hz, 1H), 7.75 (ddt, *J* = 3.9, 2.9, 1.6 Hz, 3H), 7.61 (ddd, *J* = 8.1, 6.9, 1.1 Hz, 1H), 7.58 – 7.54 (m, 2H), 7.49 – 7.45 (m, 1H).

^{13}C NMR ($CDCl_3$, 101 MHz) δ: 149.9, 147.3, 137.9, 133.8, 133.2, 129.4, 129.2 (2C), 128.1, 128.0 (2C), 127.4, 127.0.

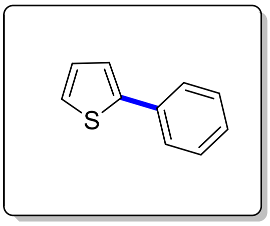

2-Phenylthiophene (CAS: 825-55-8)[10]

^{1}H NMR (CDCl$_3$, 400 MHz) δ: 7.64 (d, *J* = 7.7 Hz, 2H), 7.41 (t, *J* = 7.7 Hz, 2H), 7.35 – 7.30 (m, 3H), 7.12 – 7.10 (m, 1H).

^{13}C NMR (CDCl$_3$, 101 MHz) δ: 144.4, 134.4, 128.9, 128.0, 127.4, 126.0, 124.8, 123.1.

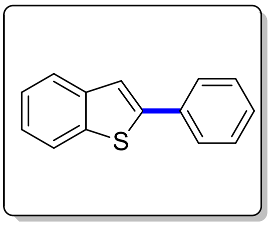

2-Phenylbenzo[b]thiophene (CAS: 1207-95-0)[24]

^{1}H NMR (CDCl$_3$, 500 MHz) δ: 7.86 (d, *J* = 7.9 Hz, 1H), 7.80 (d, *J* = 7.9 Hz, 1H), 7.75 (d, *J* = 7.2 Hz, 2H), 7.58 (s, 1H), 7.46 (t, *J* = 7.6 Hz, 2H), 7.39 – 7.33 (m, 3H).

^{13}C NMR (CDCl$_3$, 126 MHz) δ: 144.2, 140.7, 139.5, 134.3, 128.9, 128.3, 126.5, 124.5, 124.3, 123.6, 122.3, 119.4.

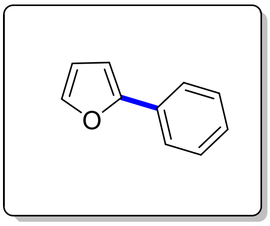

2-Phenylfuran (CAS: 17113-33-6)[25]

^{1}H NMR (CDCl$_3$, 500 MHz) δ: 7.70 (dd, *J* = 8.3, 1.1 Hz, 2H), 7.49 (d, *J* = 1.3 Hz, 1H), 7.41 (t, *J* = 7.8 Hz, 2H), 7.25 – 7.29 (m, 1H), 6.68 (d, *J* = 3.3 Hz, 1H), 6.50 (dd, *J* = 3.3, 1.8 Hz, 1H).

^{13}C NMR (CDCl$_3$, 126 MHz) δ: 154.0, 142.0, 130.9, 128.6, 127.3, 123.8, 111.6, 104.9.

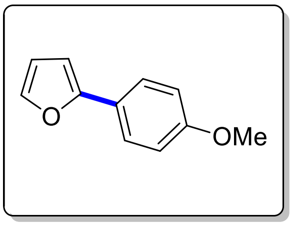

2-(4-Methoxyphenyl)furan (CAS: 17113-31-4)[25]

^{1}H NMR (CDCl$_3$, 400 MHz) δ: 7.63 (d, *J* = 8.5 Hz, 2H), 7.45 (s, 1H), 6.95 (d, *J* = 8.6 Hz, 2H), 6.54 (d, *J* = 3.3 Hz, 1H), 6.47 (dd, *J* = 3.0, 2.1 Hz, 1H), 3.86 (s, 3H).

^{13}C NMR (CDCl$_3$, 101 MHz) δ: 159.0, 154.0, 141.4, 125.2, 124.3, 114.1, 111.5, 103.4, 55.3.

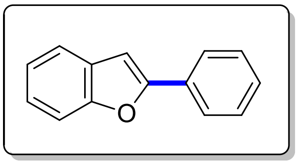

2-Phenylbenzofuran (CAS: 1839-72-1)[26]

^{1}H NMR ($CDCl_3$, 400 MHz) δ: 7.90 (d, *J* = 7.7 Hz, 2H), 7.62 (d, *J* = 7.6 Hz, 1H), 7.56 (d, *J* = 8.1 Hz, 1H), 7.48 (t, *J* = 7.7 Hz, 2H), 7.38 (t, *J* = 7.4 Hz, 1H), 7.32 – 7.26 (m, 2H), 7.06 (s, 1H).

^{13}C NMR ($CDCl_3$, 101 MHz) δ: 155.9, 154.9, 130.5, 129.2, 128.8, 128.5, 124.9, 124.2, 122.9, 120.9, 111.2, 101.3.

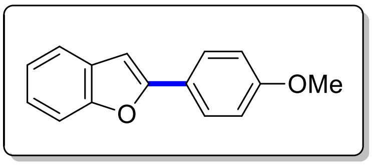

2-(4-Methoxyphenyl)benzofuran (CAS: 19234-04-9)[27]

^{1}H NMR ($CDCl_3$, 400 MHz) δ: 7.83 (d, *J* = 8.5 Hz, 2H), 7.58 (d, *J* = 7.6 Hz, 1H), 7.52 (d, *J* = 8.0 Hz, 1H), 7.28 – 7.20 (m, 2H), 7.01 (d, *J* = 8.5 Hz, 2H), 6.91 (s, 1H), 3.89 (s, 3H).

^{13}C NMR ($CDCl_3$, 101 MHz) δ: 160.0, 156.0, 154.7, 129.5, 126.4, 123.7, 123.4, 122.8, 120.6, 114.3, 111.0, 99.7, 55.4.

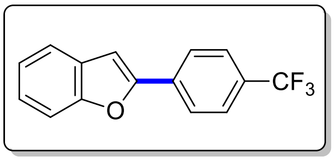

2-(4-(Trifluoromethyl)phenyl)benzofuran (CAS: 904325-87-7)[26]

^{1}H NMR ($CDCl_3$, 500 MHz) δ: 7.99 (d, *J* = 8.2 Hz, 2H), 7.73 (d, *J* = 8.3 Hz, 2H), 7.65 (d, *J* = 7.8 Hz, 1H), 7.57 (d, *J* = 8.2 Hz, 1H), 7.38 – 7.34 (m, 1H), 7.29 (d, *J* = 1.8 Hz, 1H), 7.17 (s, 1H).

^{13}C NMR ($CDCl_3$, 126 MHz) δ: 155.1, 154.2, 133.7, 130.1 (q, J^F = 32.3 Hz), 128.8, 125.8 (q, J^F = 3.8 Hz), 125.1, 125.0, 124.0 (q, J^F = 271.5 Hz), 123.2, 121.3, 111.4, 103.3.

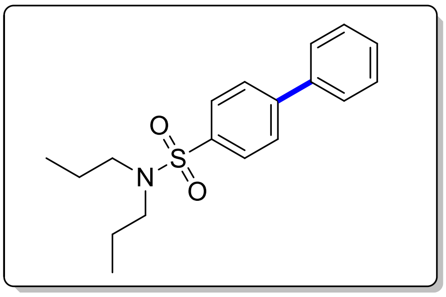

N,N-Dipropyl-[1,1'-biphenyl]-4-sulfonamide (CAS: 2326074-88-6)[28]

^{1}H NMR ($CDCl_3$, 400 MHz) δ: 7.89 (d, *J* = 8.4 Hz, 2H), 7.73 (d, *J* = 8.4 Hz, 2H), 7.63 (d, *J* = 7.3 Hz, 2H), 7.50 (t, *J* = 7.4 Hz, 2H), 7.43 (t, *J* = 7.3 Hz, 1H), 3.17 – 3.11 (m, 4H), 1.61 (dd, *J* = 15.1, 7.5 Hz, 4H), 0.92 (t, *J* = 7.4 Hz, 6H).

^{13}C NMR ($CDCl_3$, 101 MHz) δ: 145.0, 139.4, 138.7, 129.0, 128.3, 127.5 (2C), 127.2, 50.1, 22.1, 11.2.

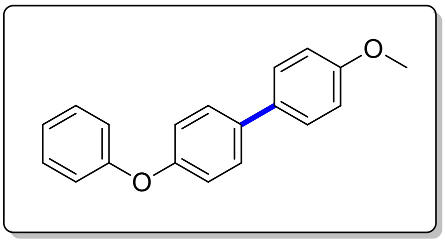

4-Methoxy-4'-phenoxy-1,1'-biphenyl (CAS: 1360567-60-7)[28]

^{1}H NMR (CDCl$_3$, 400 MHz) δ: 7.54 – 7.49 (m, 4H), 7.41 – 7.35 (m, 2H), 7.15 (d, *J* = 7.4 Hz, 1H), 7.08 (dd, *J* = 8.2, 1.9 Hz, 4H), 7.00 (d, *J* = 8.7 Hz, 2H), 3.88 (s, 3H).

^{13}C NMR (CDCl$_3$, 101 MHz) δ: 159.0, 157.3, 156.3, 136.0, 133.1, 129.7, 128.0, 127.9, 123.2, 119.1, 118.9, 114.2, 55.3.

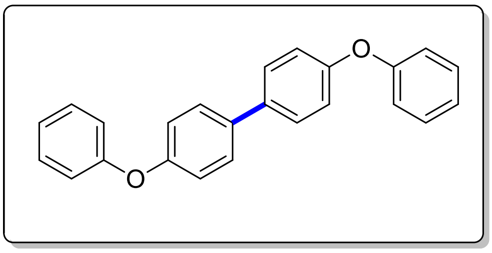

4,4'-Diphenoxy-1,1'-biphenyl (CAS: 2519-16-6)[29]

^{1}H NMR (CDCl$_3$, 400 MHz) δ: 7.56 (d, *J* = 8.6 Hz, 4H), 7.39 (t, *J* = 7.9 Hz, 4H), 7.17 (d, *J* = 7.4 Hz, 2H), 7.15 – 7.06 (m, 8H).

^{13}C NMR (CDCl$_3$, 101 MHz) δ: 157.1, 156.6, 135.6, 129.8, 128.2, 123.3, 119.1, 119.0.

Ⅳ. Supplementary Figures

NMR spectra of products

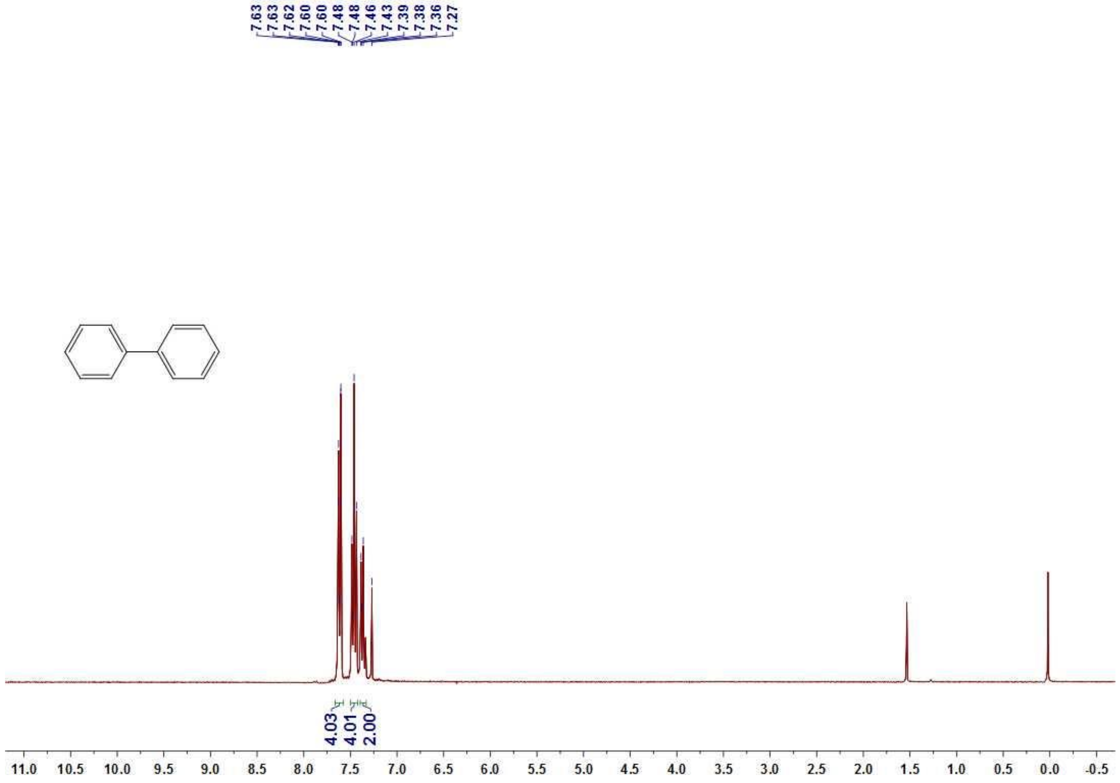

Supplementary Figure 18 ^{1}H NMR of **1,1'-biphenyl**

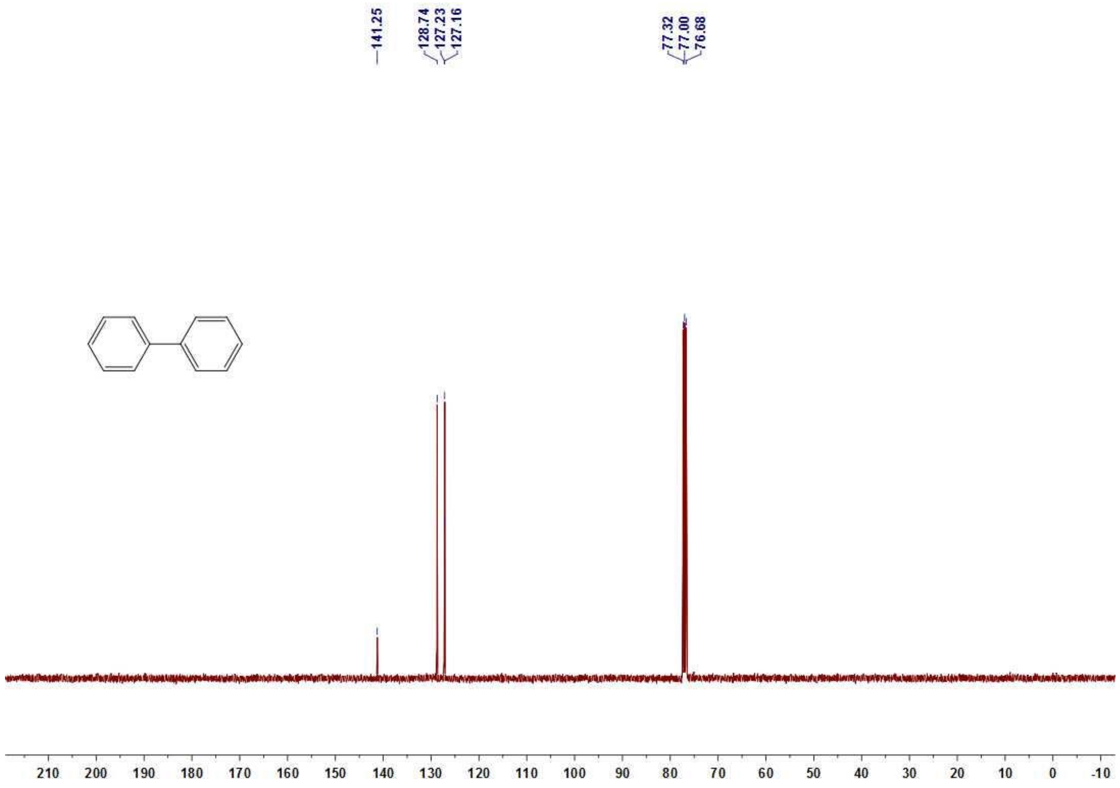

Supplementary Figure 19 ^{13}C NMR of **1,1'-biphenyl**

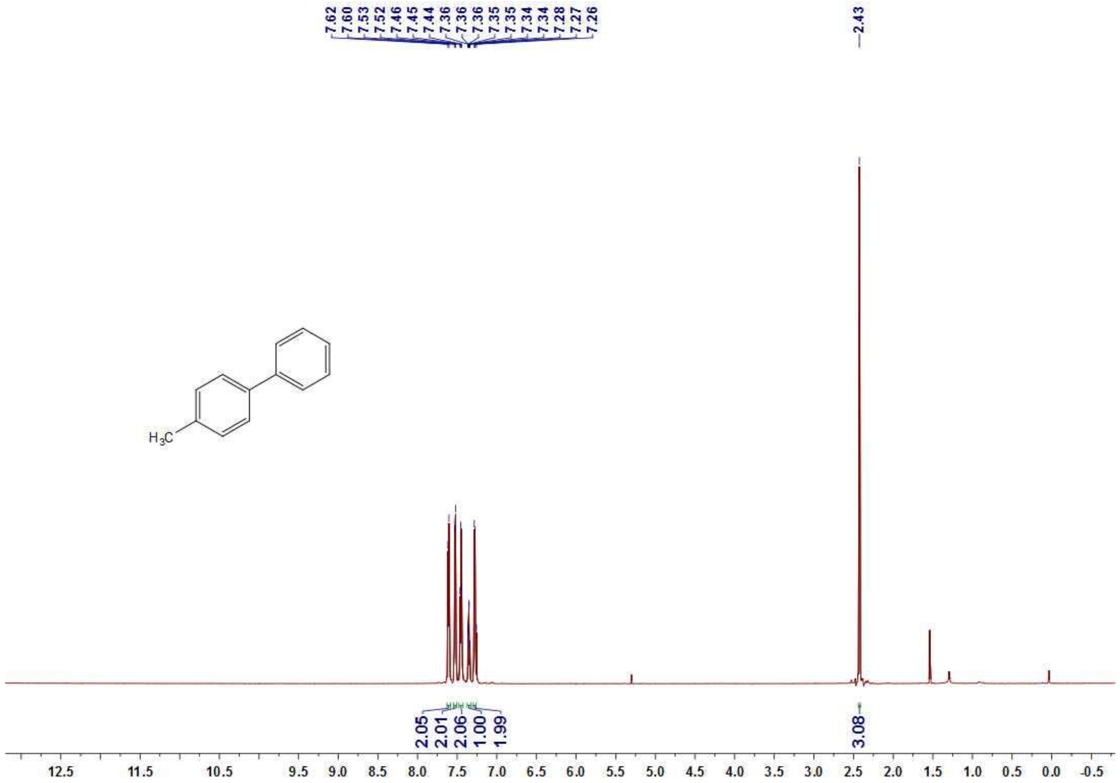

Supplementary Figure 20 ^{1}H NMR of **4-methyl-1,1'-biphenyl**

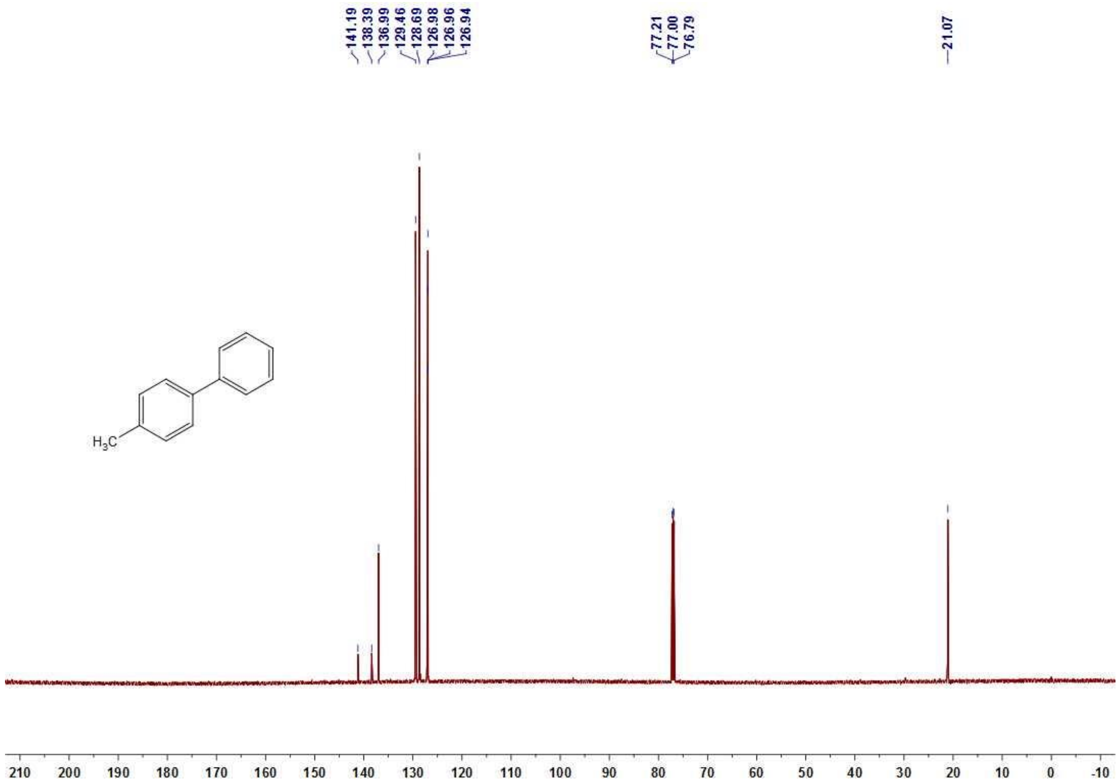

Supplementary Figure 21 ^{13}C NMR of **4-methyl-1,1'-biphenyl**

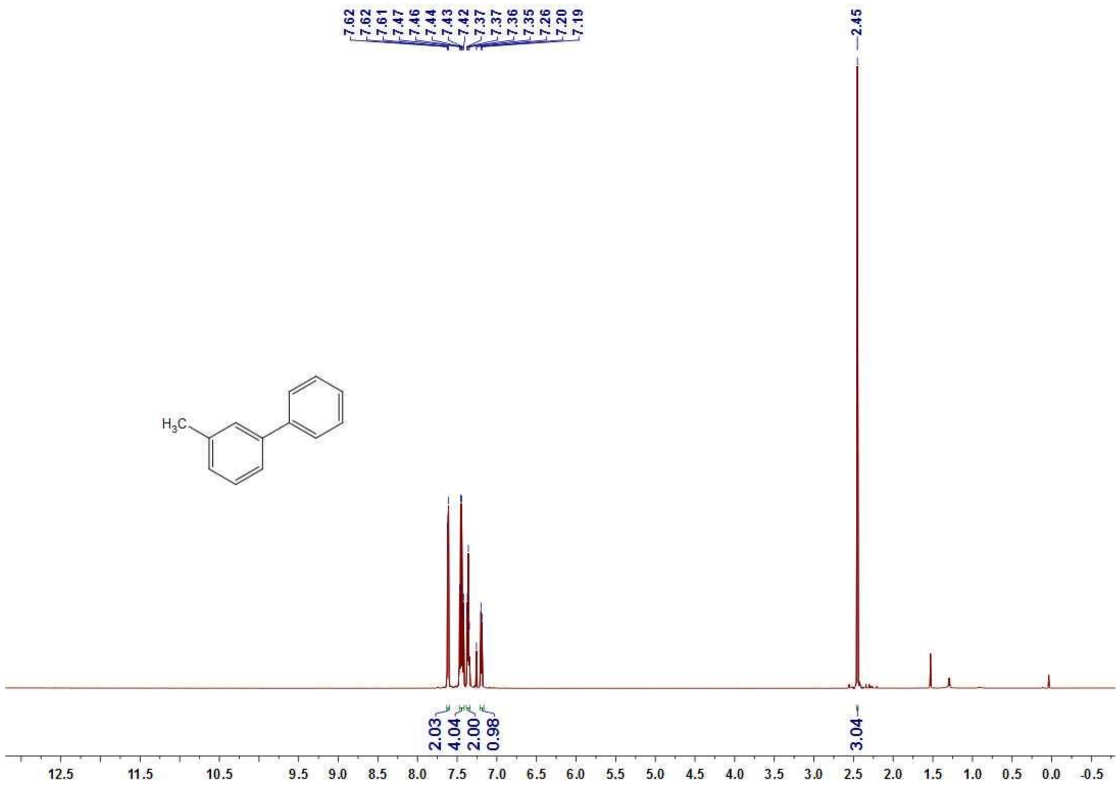

Supplementary Figure 22 ^{1}H NMR of **3-methyl-1,1'-biphenyl**

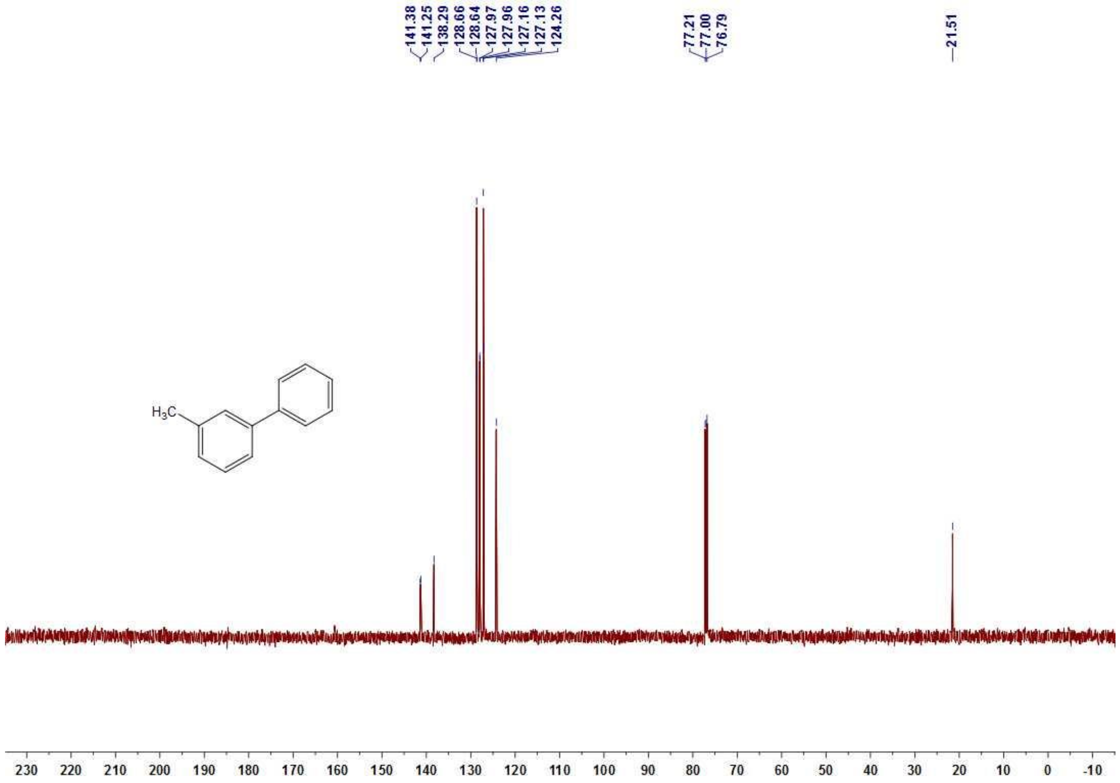

Supplementary Figure 23 ^{13}C NMR of **3-methyl-1,1'-biphenyl**

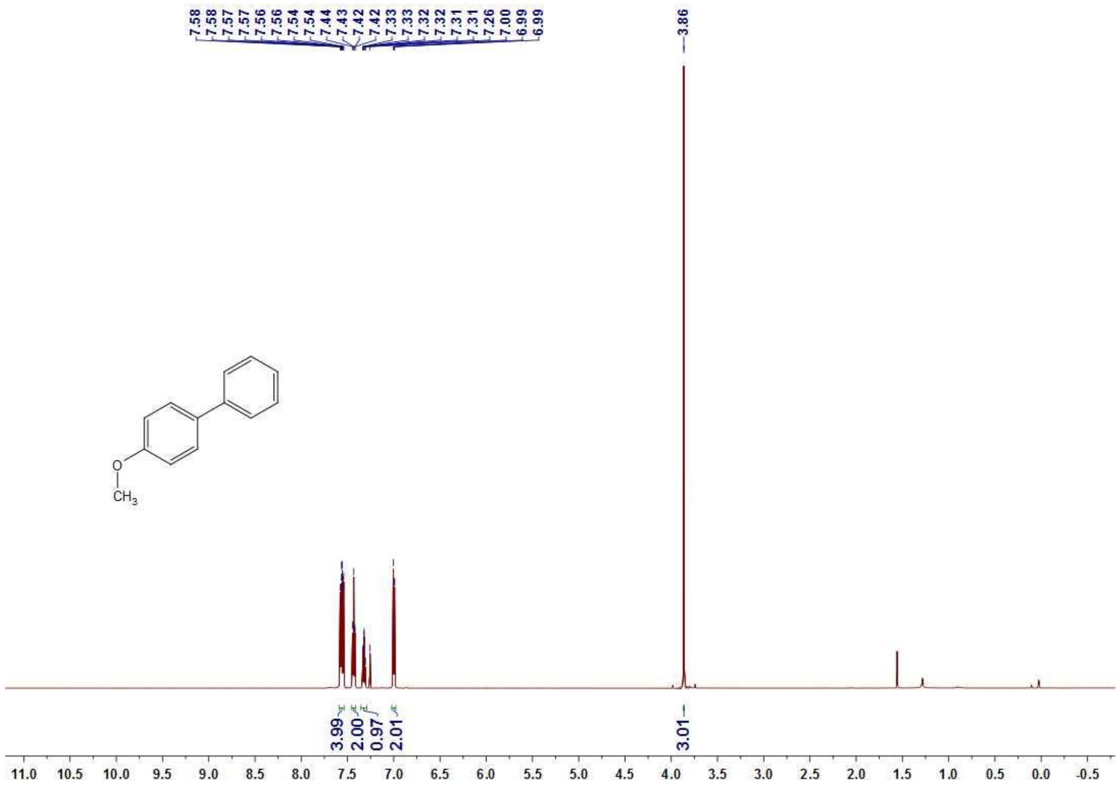

Supplementary Figure 24 ^{1}H NMR of **4-methoxy-1,1'-biphenyl**

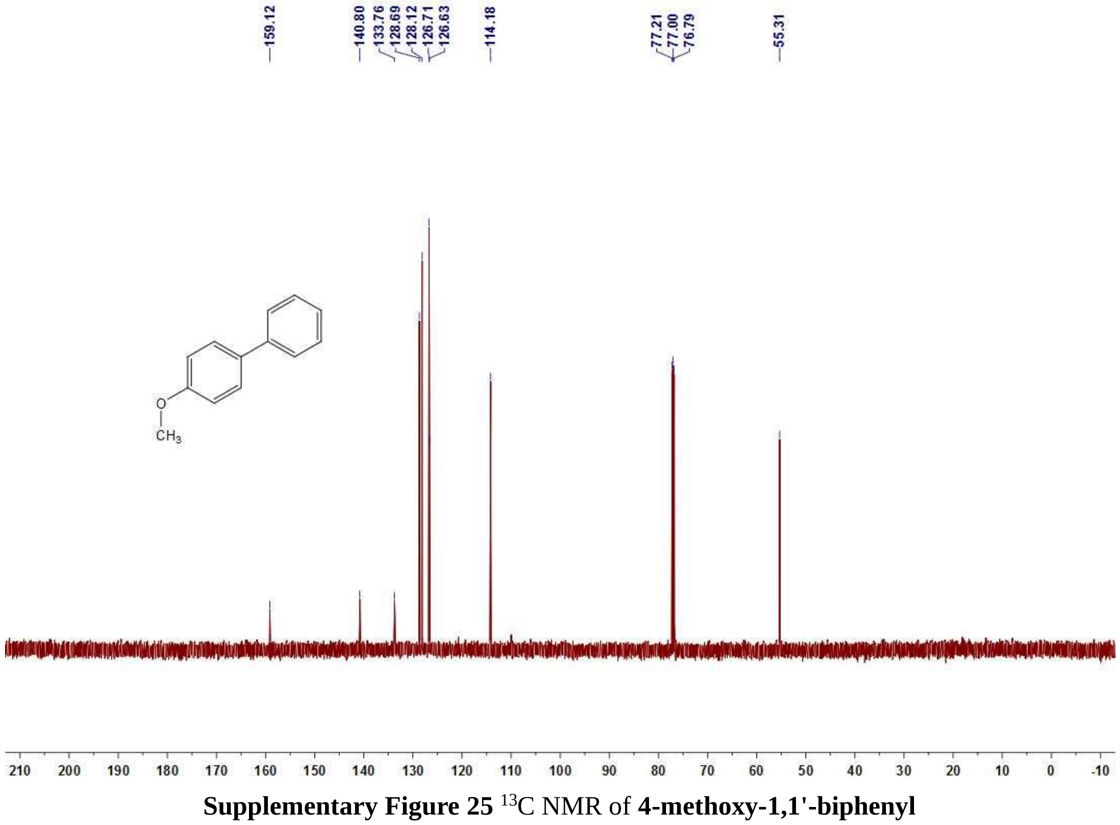

Supplementary Figure 25 ^{13}C NMR of **4-methoxy-1,1'-biphenyl**

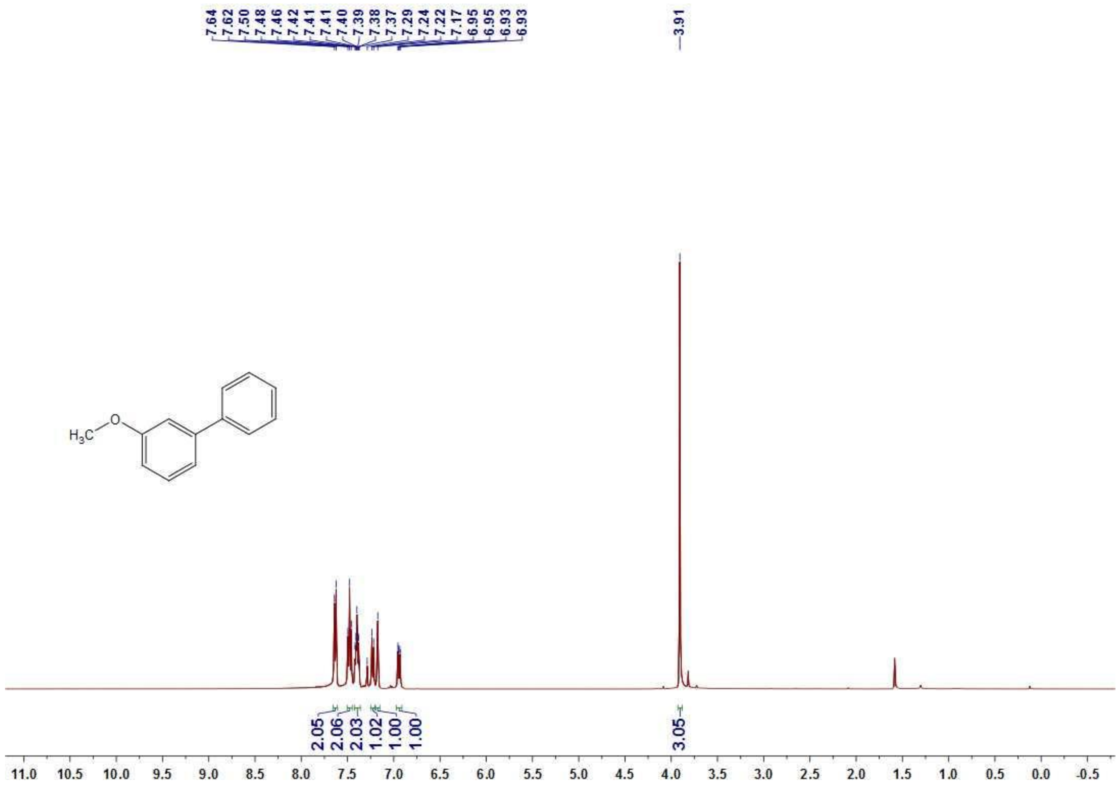

Supplementary Figure 26 ^{1}H NMR of **3-methoxy-1,1'-biphenyl**

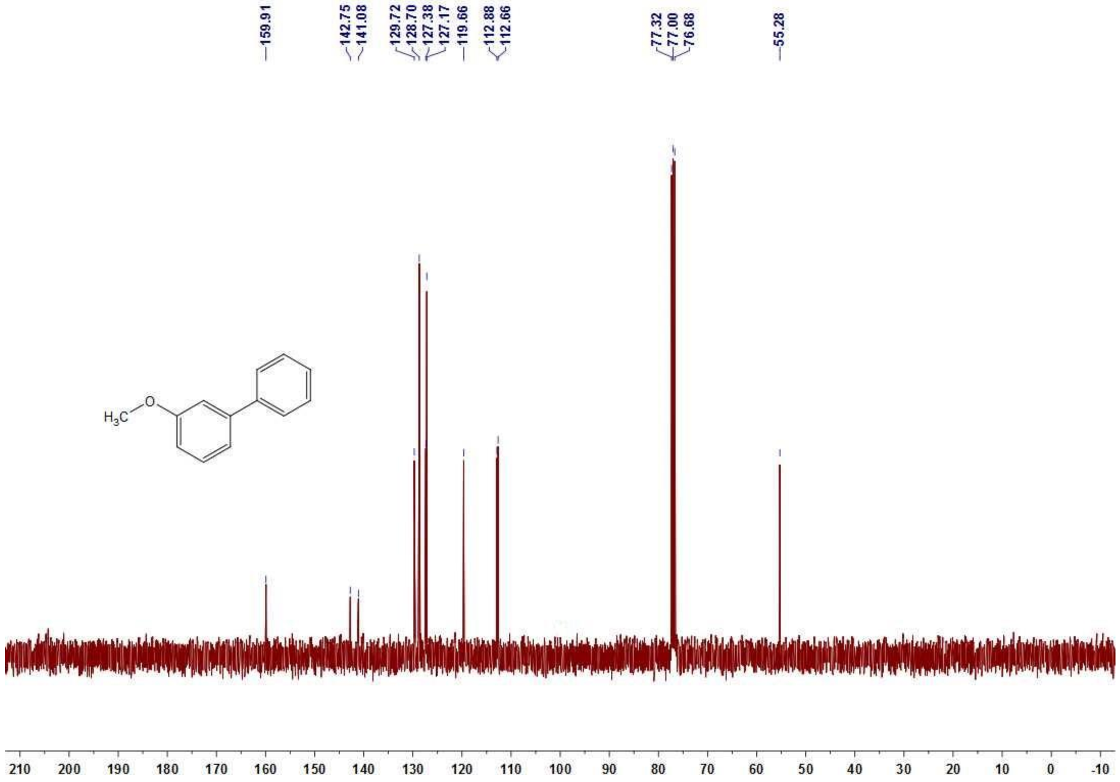

Supplementary Figure 27 ^{13}C NMR of **3-methoxy-1,1'-biphenyl**

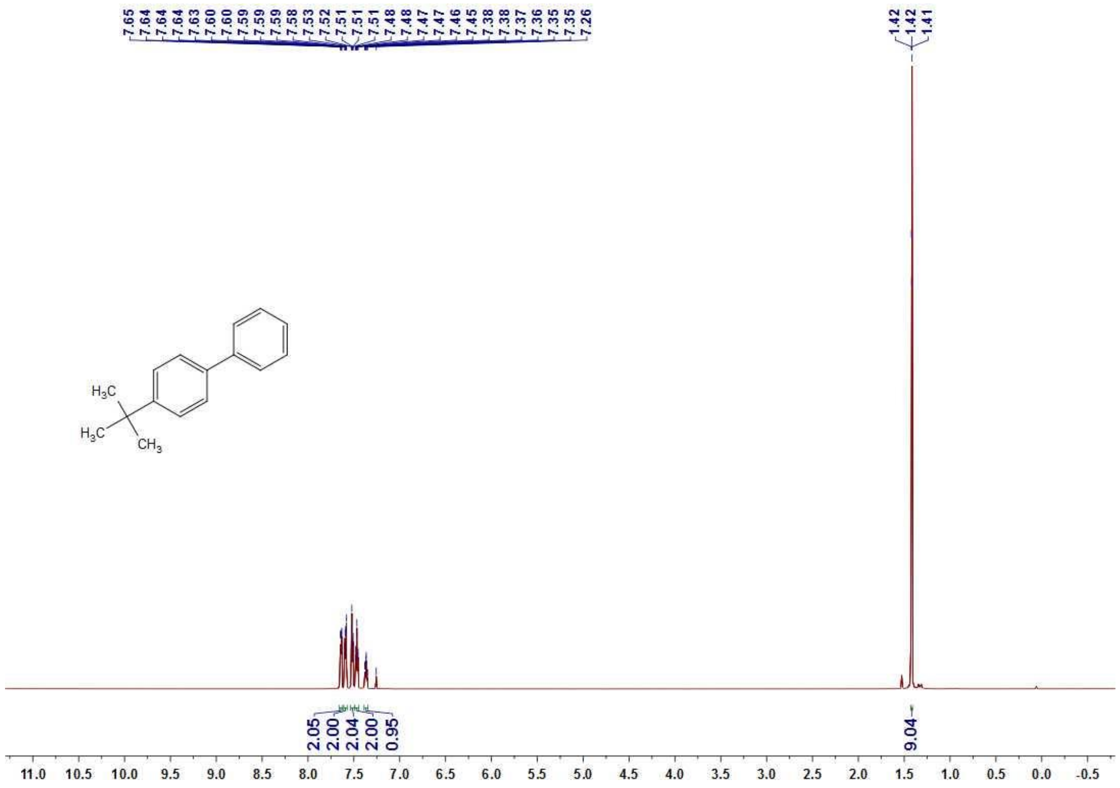

Supplementary Figure 28 ^{1}H NMR of **4-(tert-butyl)-1,1'-biphenyl**

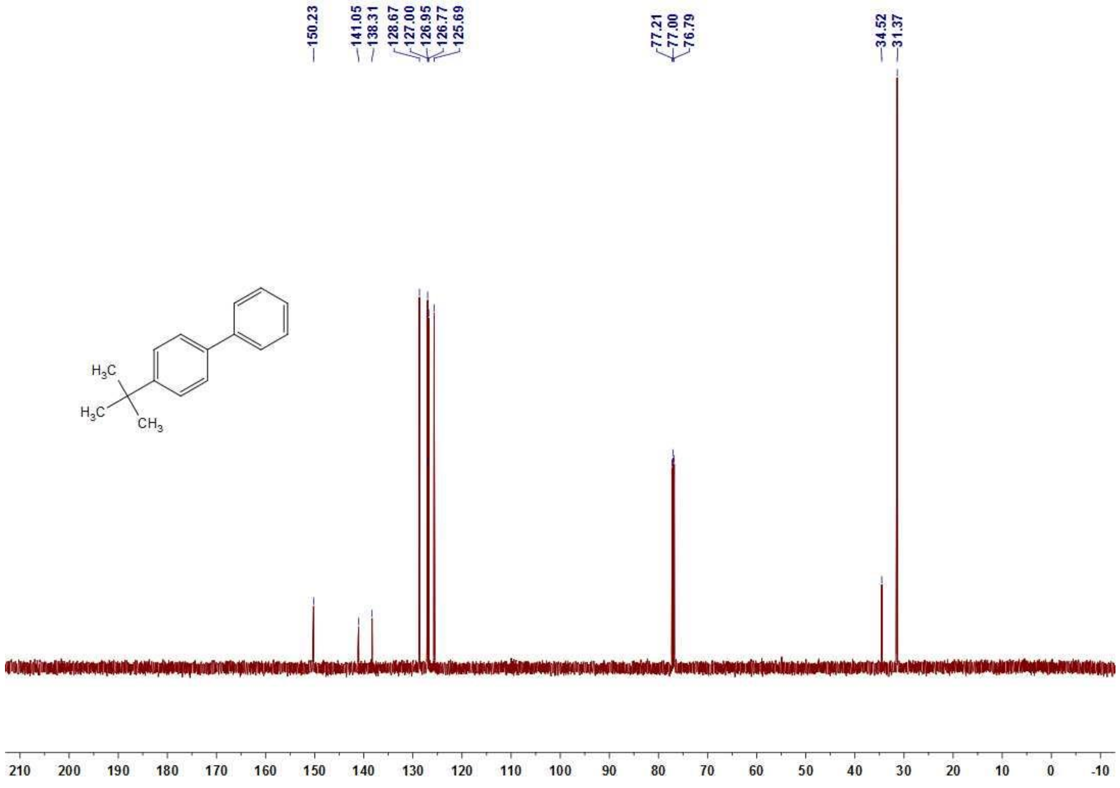

Supplementary Figure 29 ^{13}C NMR of **4-(tert-butyl)-1,1'-biphenyl**

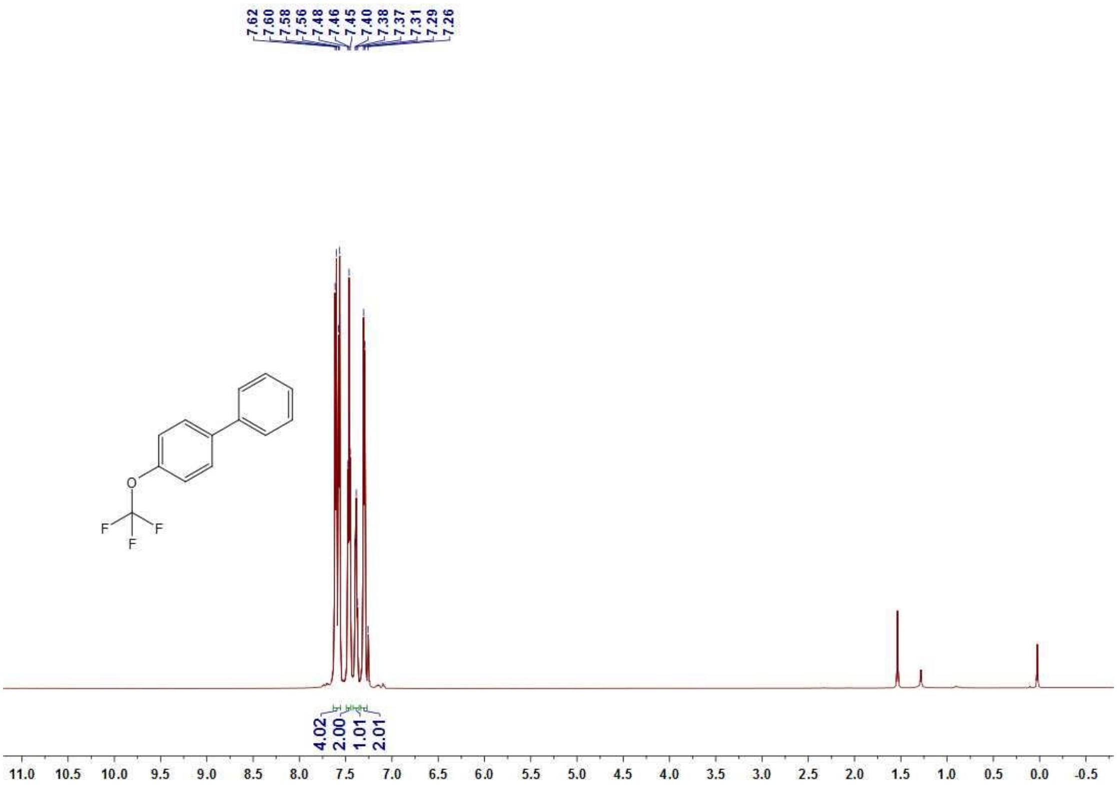

Supplementary Figure 30 ^{1}H NMR of **4-(Trifluoromethoxy)-1,1'-biphenyl**

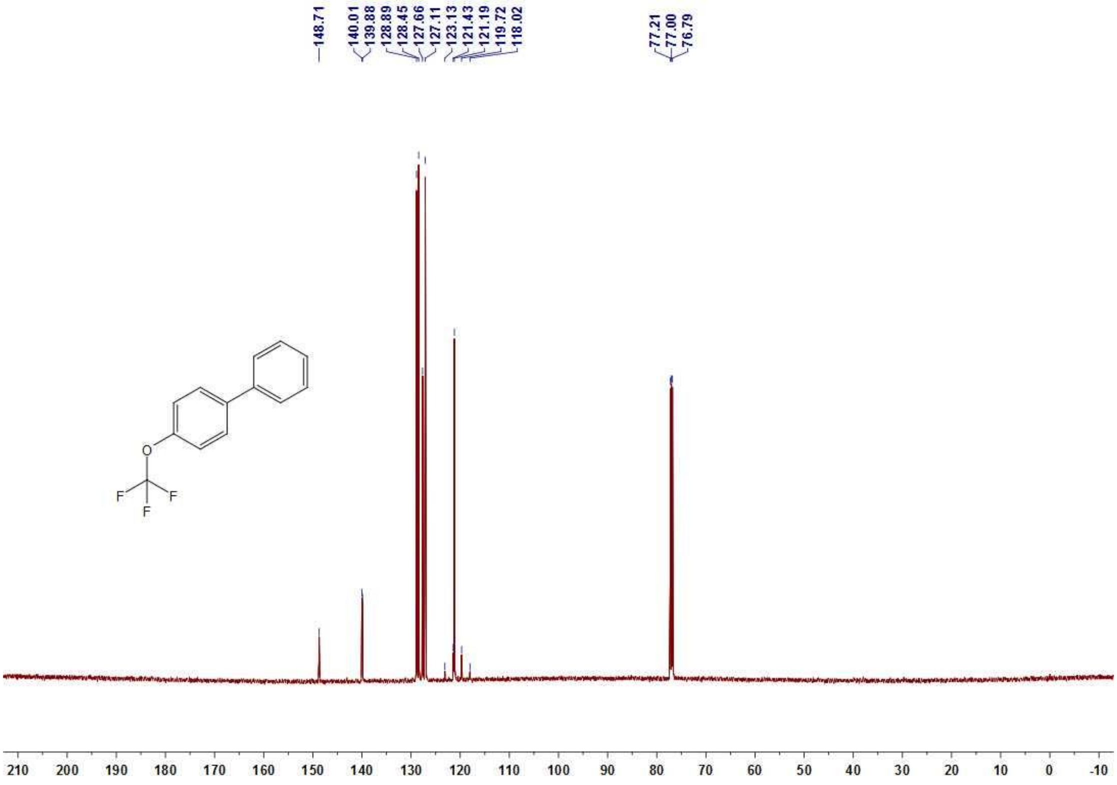

Supplementary Figure 31 ^{13}C NMR of **4-(Trifluoromethoxy)-1,1'-biphenyl**

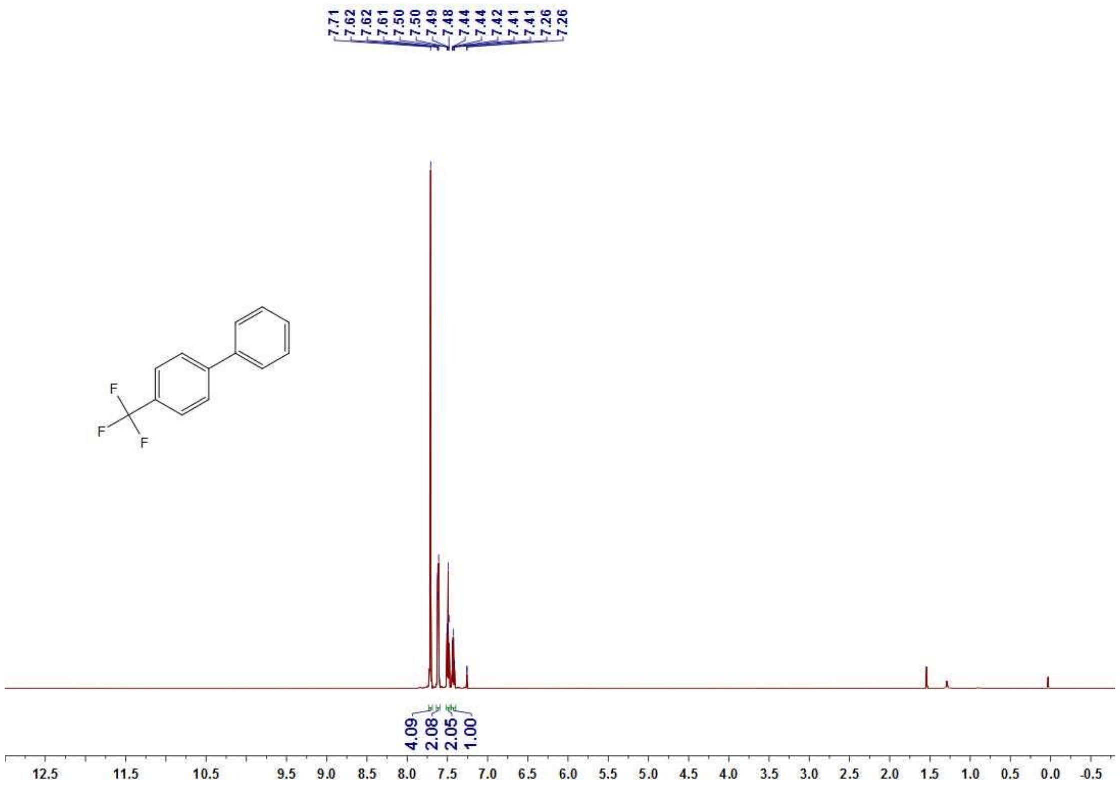

Supplementary Figure 32 ^{1}H NMR of **4-(trifluoromethyl)-1,1'-biphenyl**

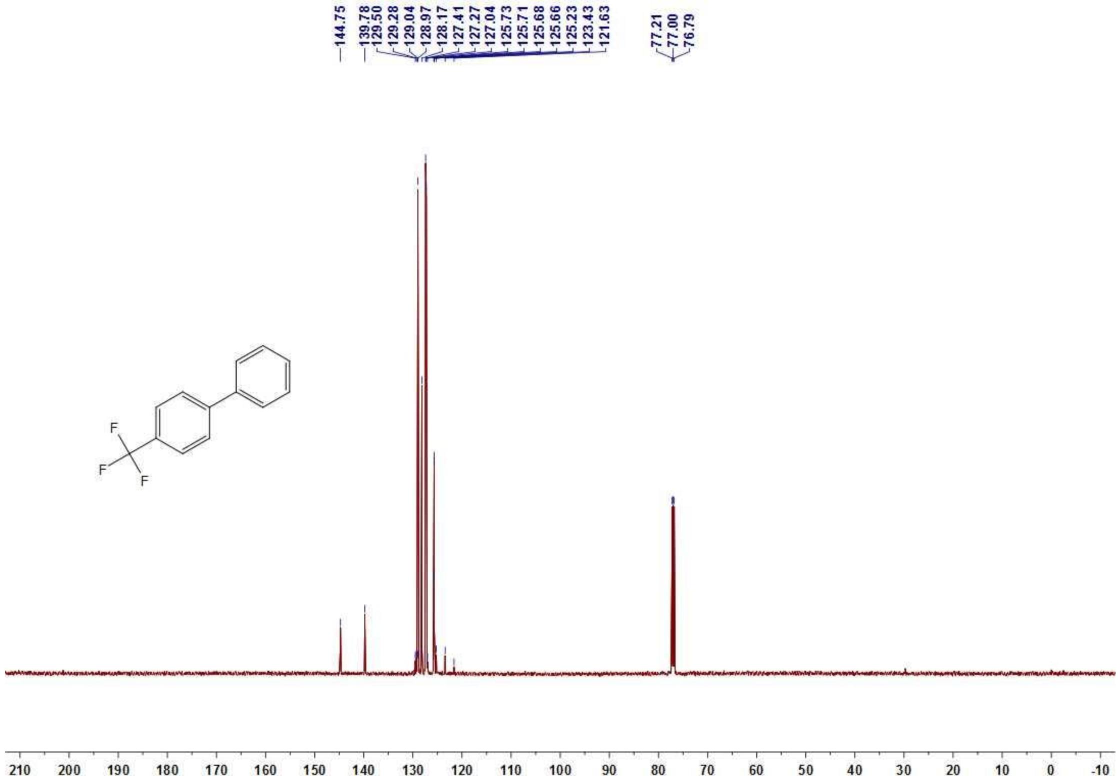

Supplementary Figure 33 ^{13}C NMR of **4-(trifluoromethyl)-1,1'-biphenyl**

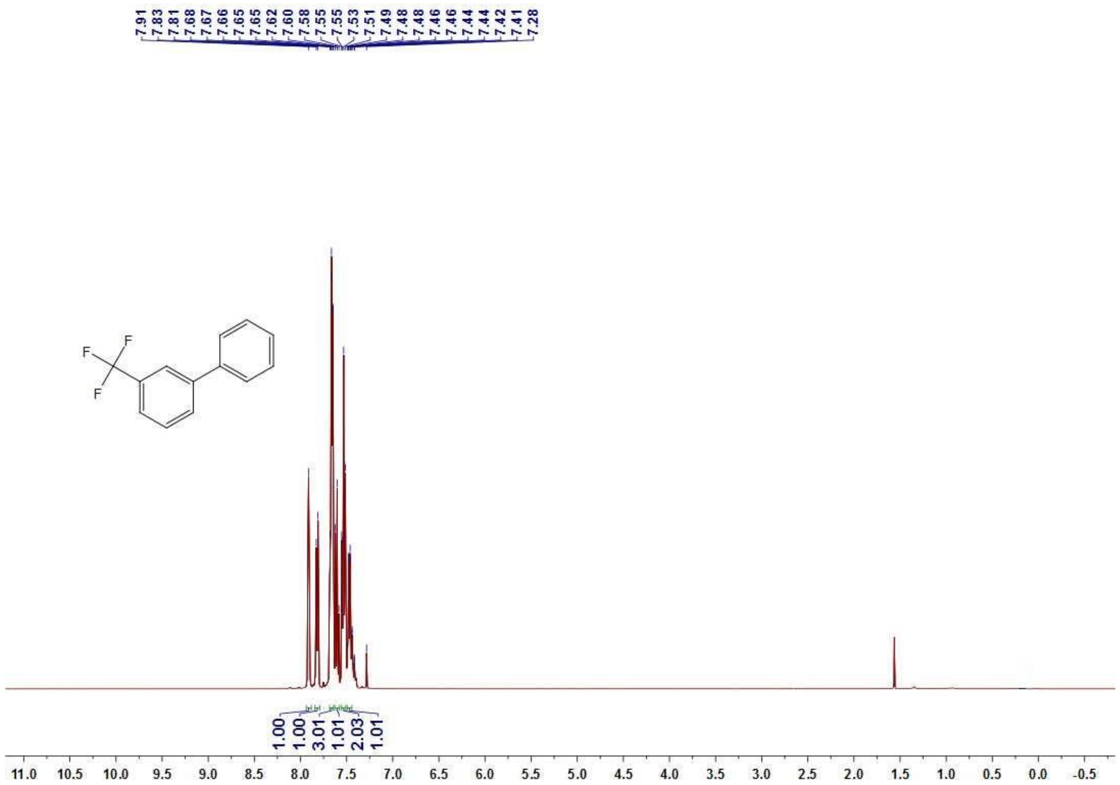

Supplementary Figure 34 ^{1}H NMR of **3-(trifluoromethyl)-1,1'-biphenyl**

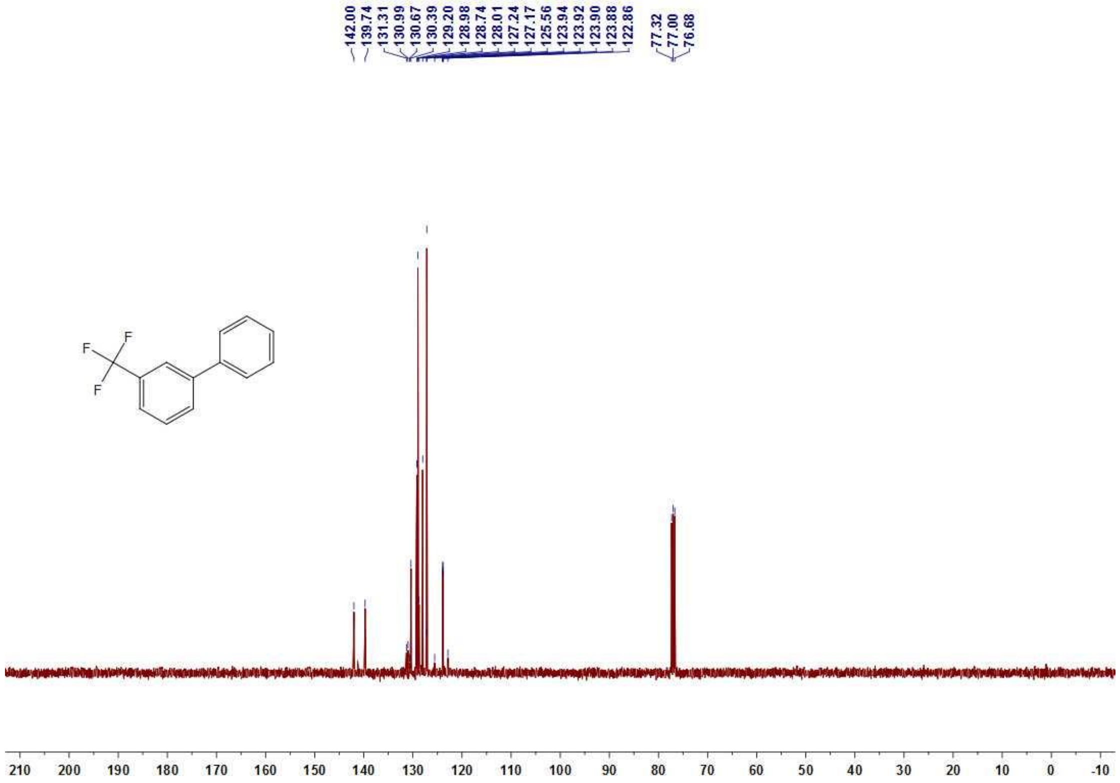

Supplementary Figure 35 ^{13}C NMR of **3-(trifluoromethyl)-1,1'-biphenyl**

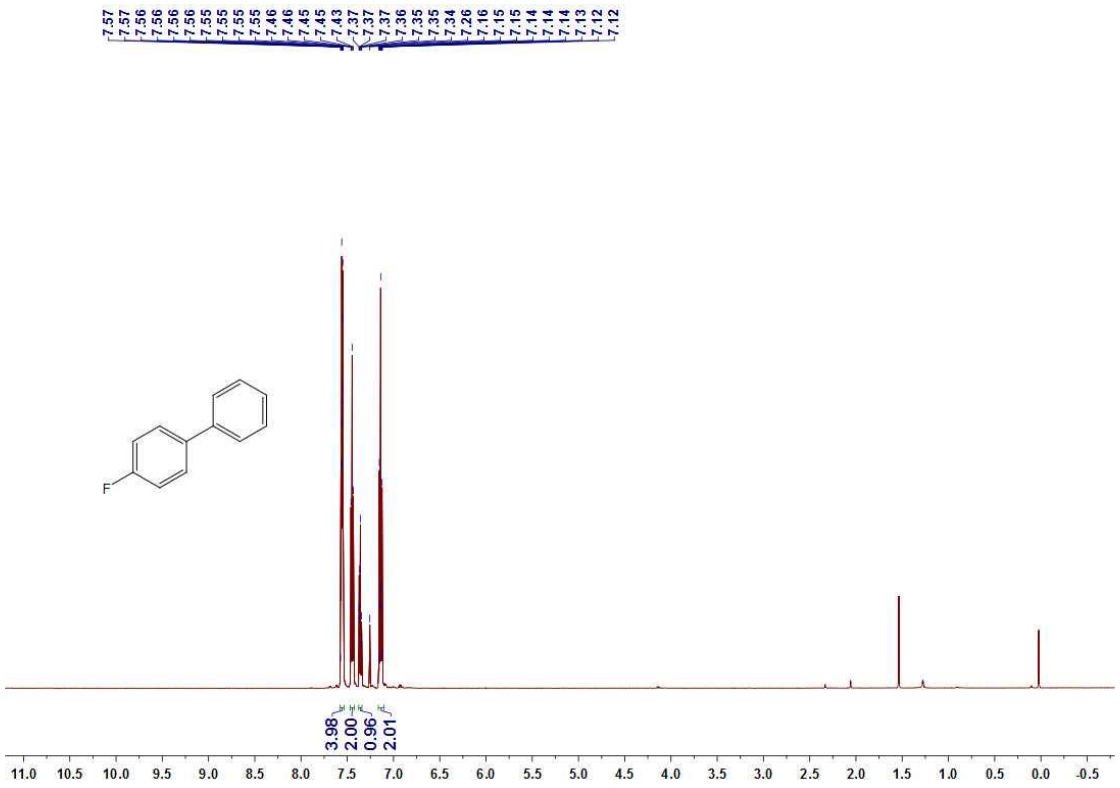

Supplementary Figure 36 ^{1}H NMR of **4-fluoro-1,1'-biphenyl**

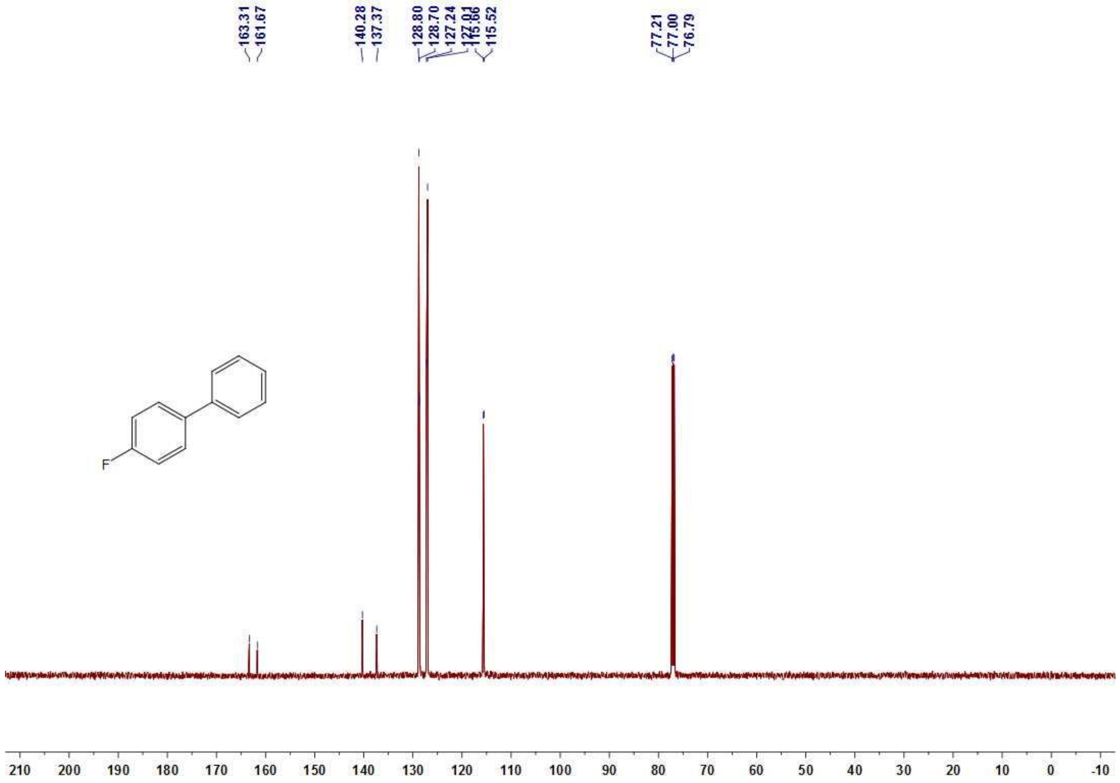

Supplementary Figure 37 ^{13}C NMR of **4-fluoro-1,1'-biphenyl**

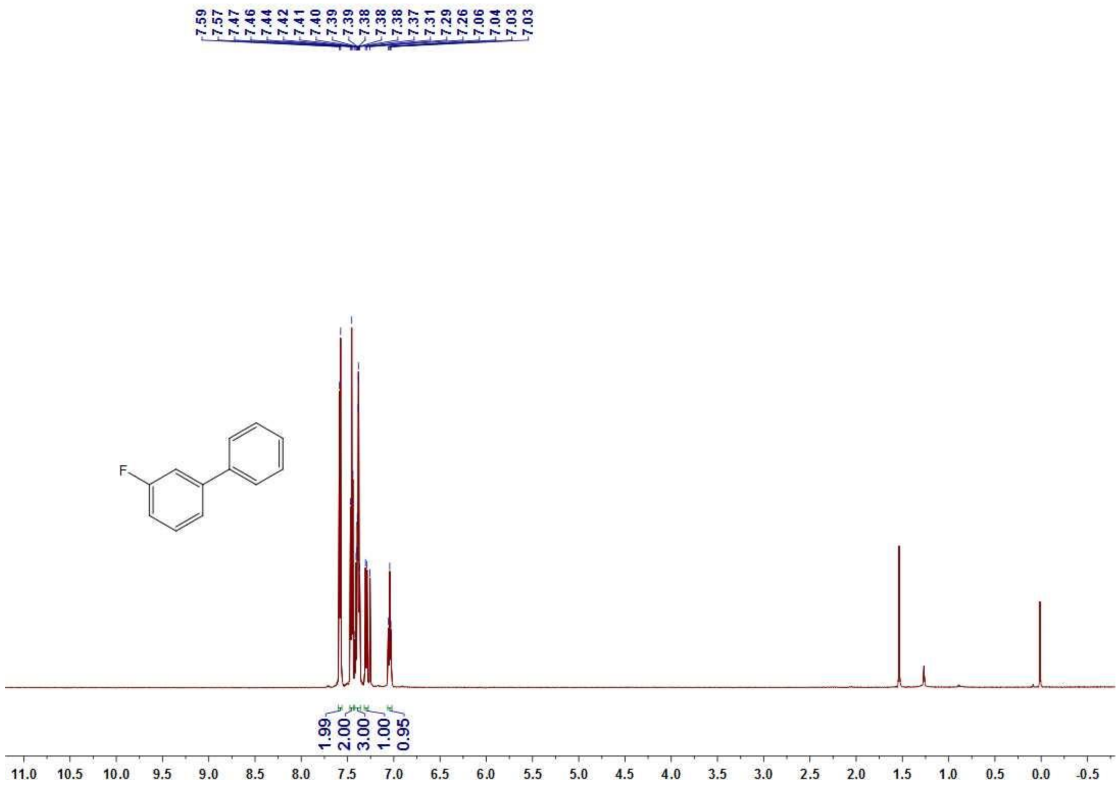

Supplementary Figure 38 ^{1}H NMR of **3-fluoro-1,1'-biphenyl**

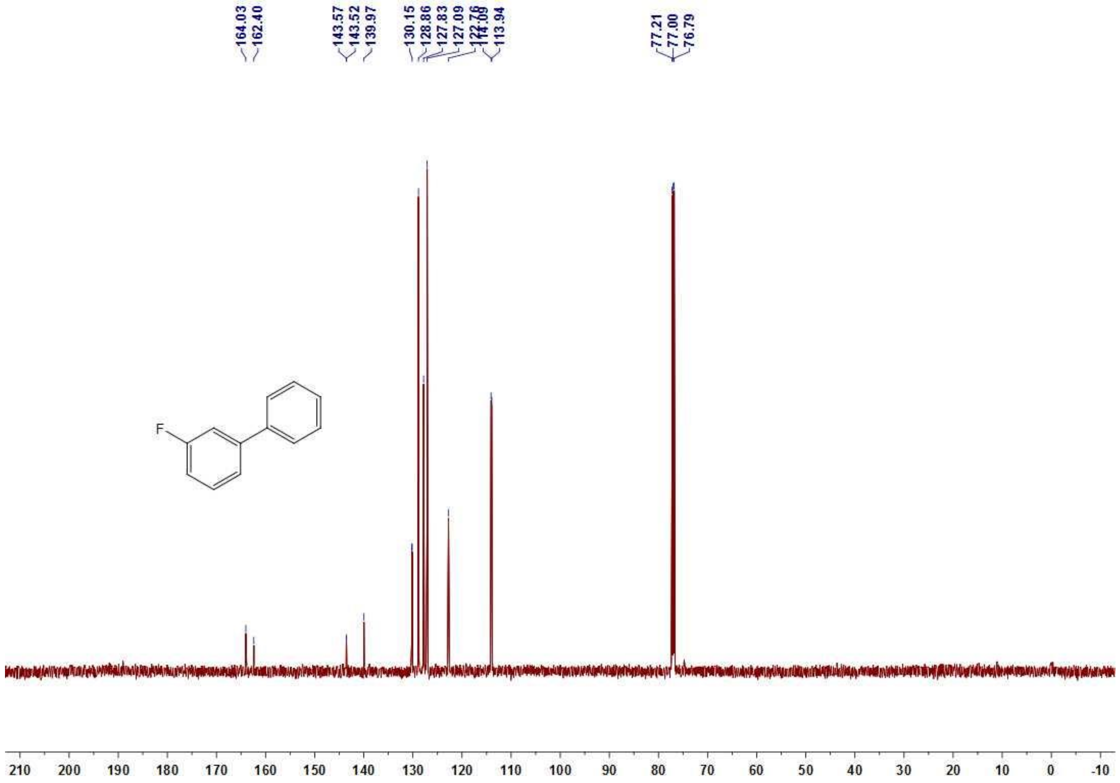

Supplementary Figure 39 ^{13}C NMR of **3-fluoro-1,1'-biphenyl**

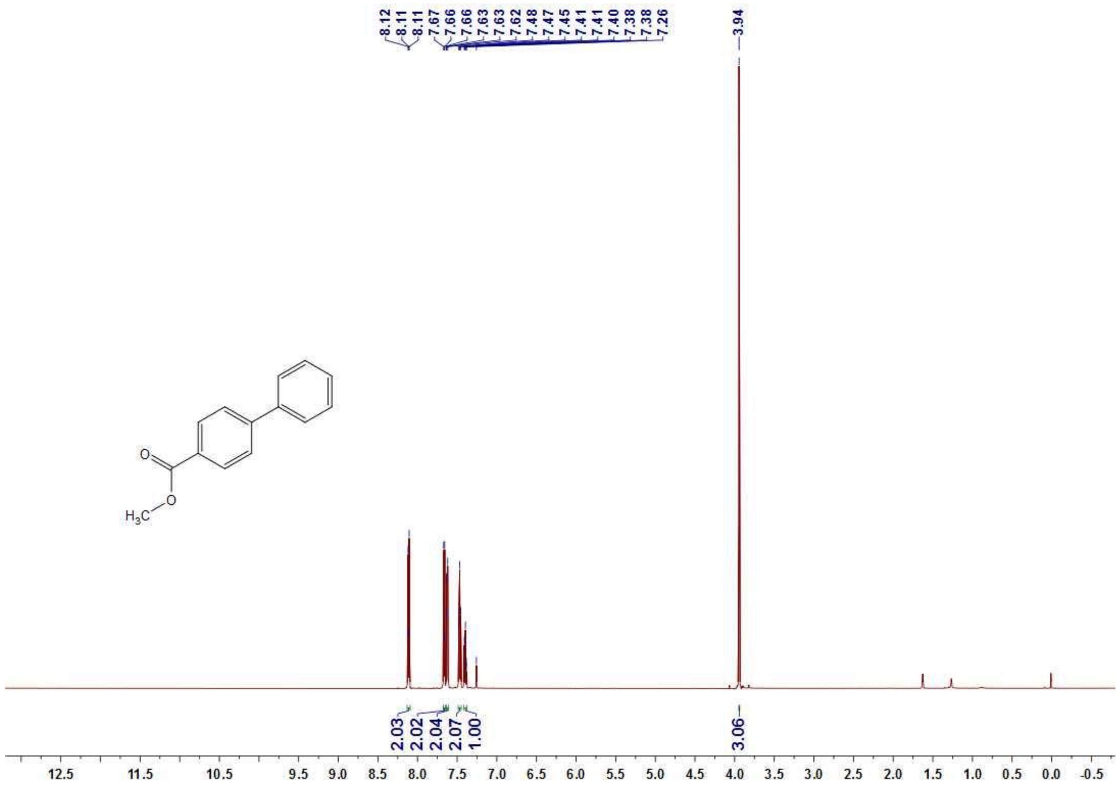

Supplementary Figure 40 ^{1}H NMR of **methyl [1,1'-biphenyl]-4-carboxylate**

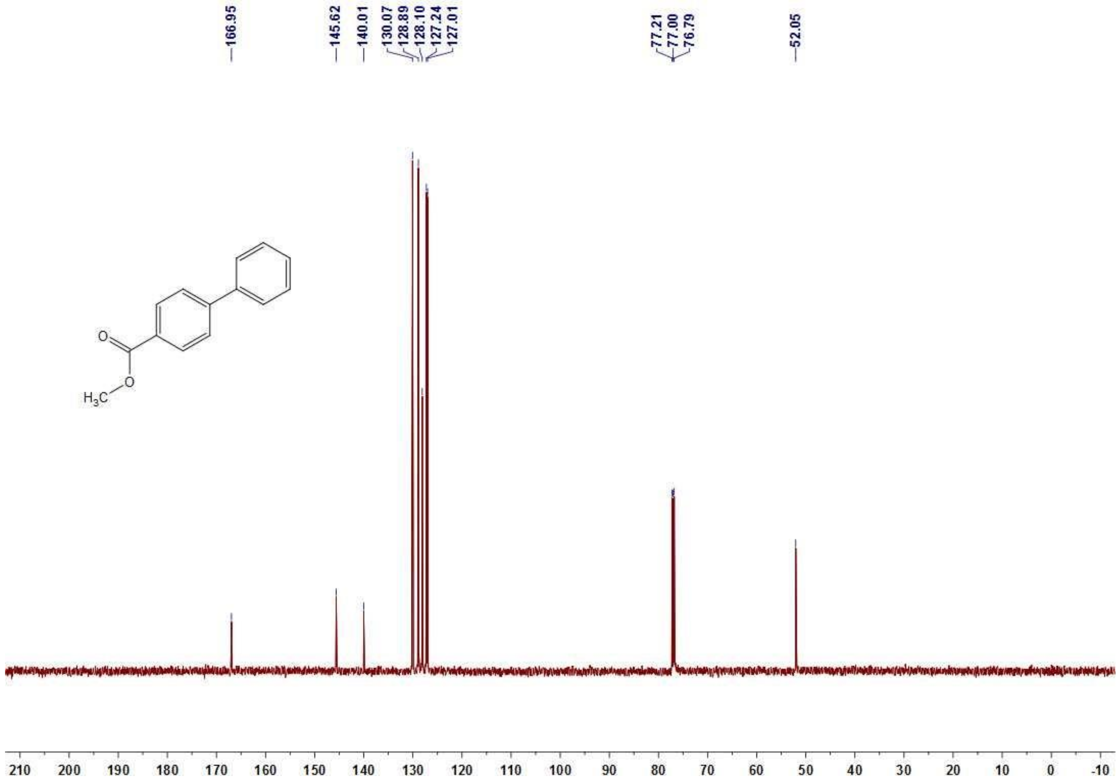

Supplementary Figure 41 ^{13}C NMR of **methyl [1,1'-biphenyl]-4-carboxylate**

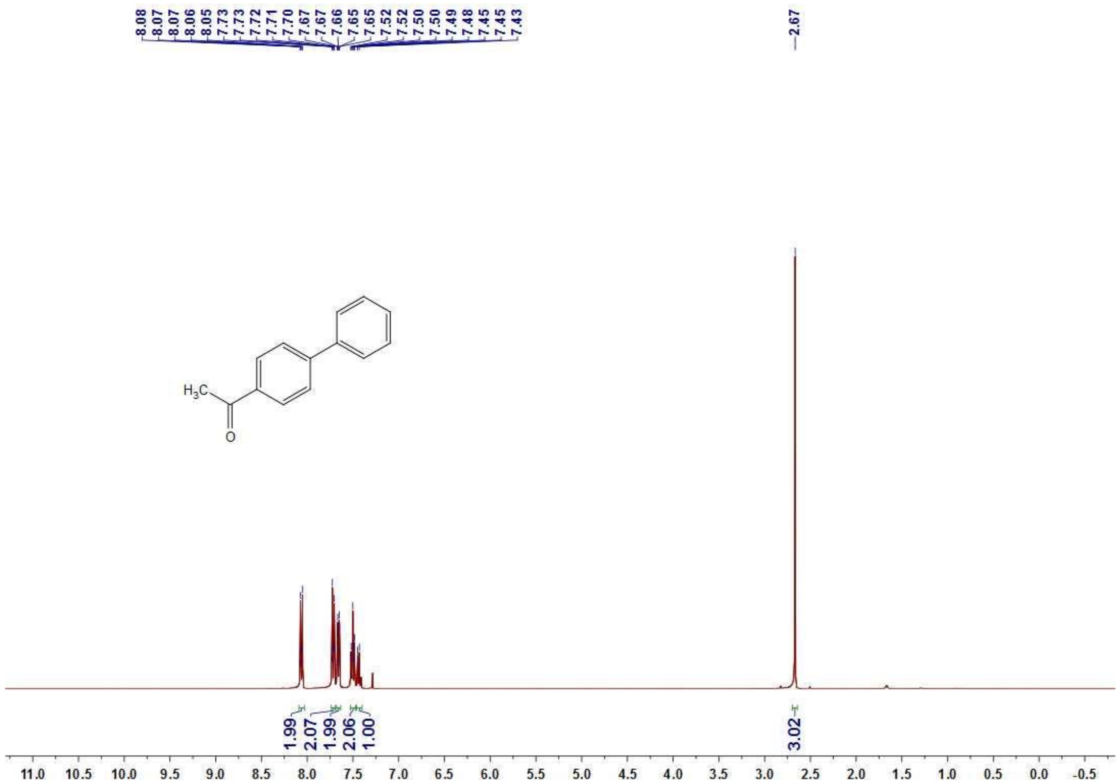

Supplementary Figure 42 ^{1}H NMR of **4-acetylbiphenyl**

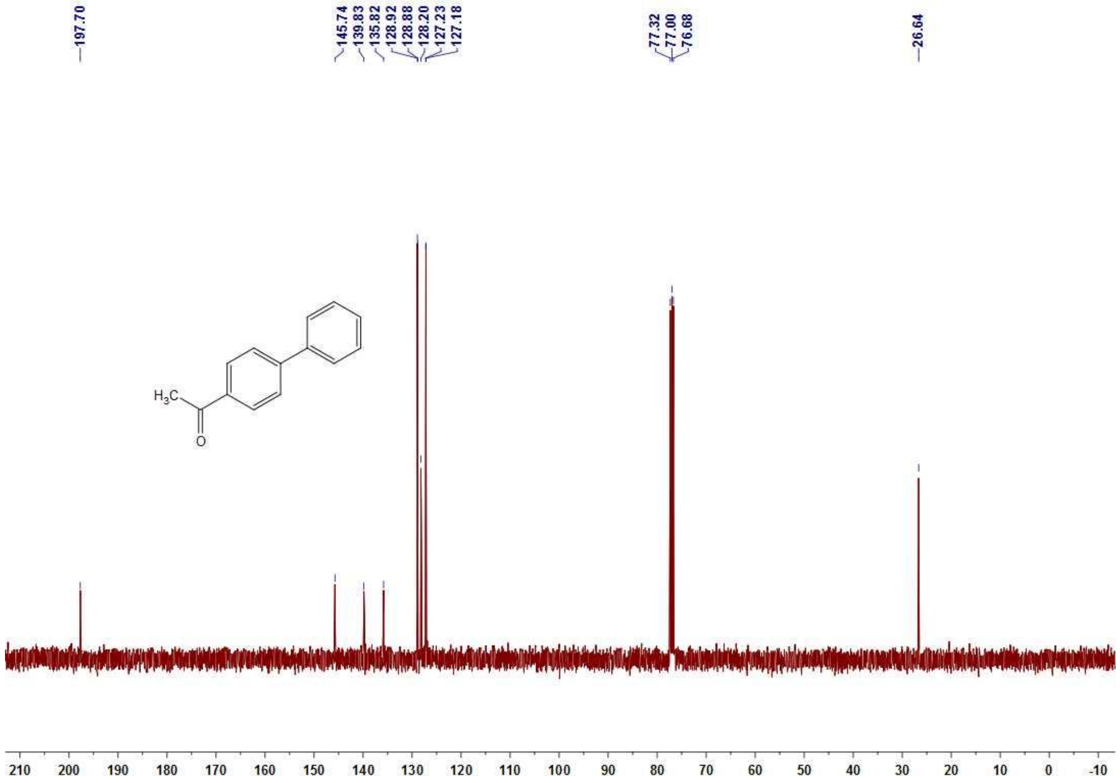

Supplementary Figure 43 ^{13}C NMR of **4-acetylbiphenyl**

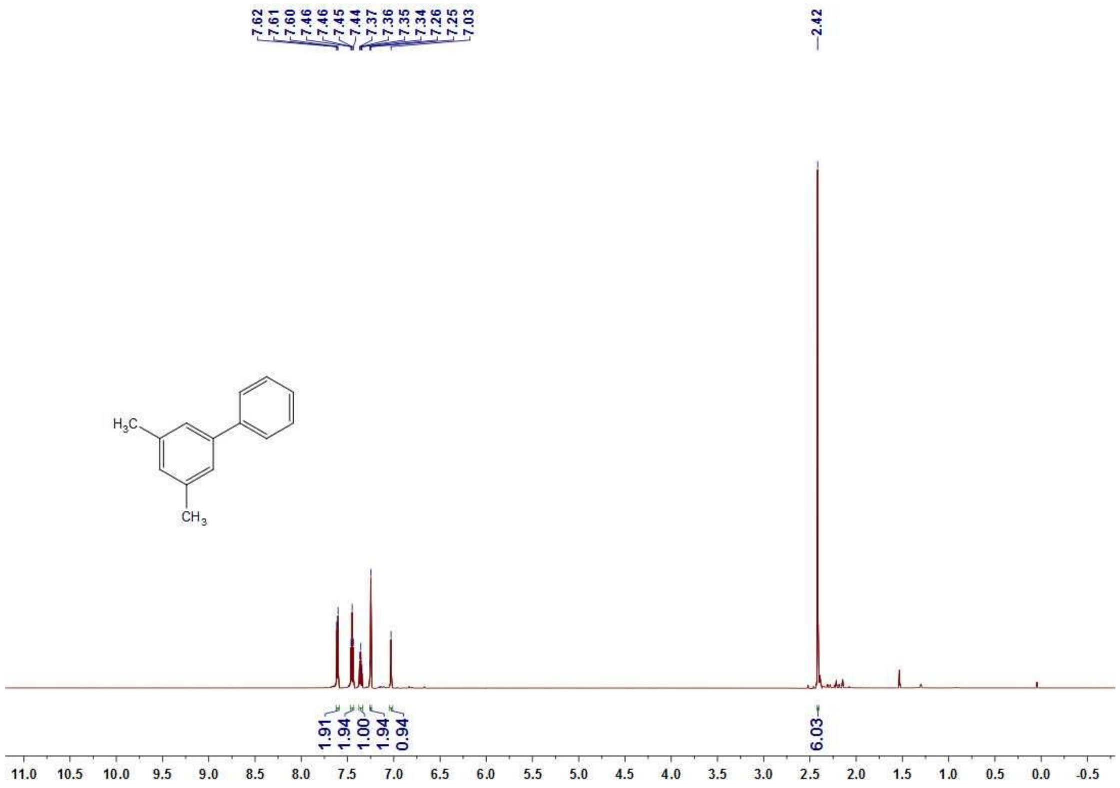

Supplementary Figure 44 ^{1}H NMR of **3,5-dimethyl-1,1'-biphenyl**

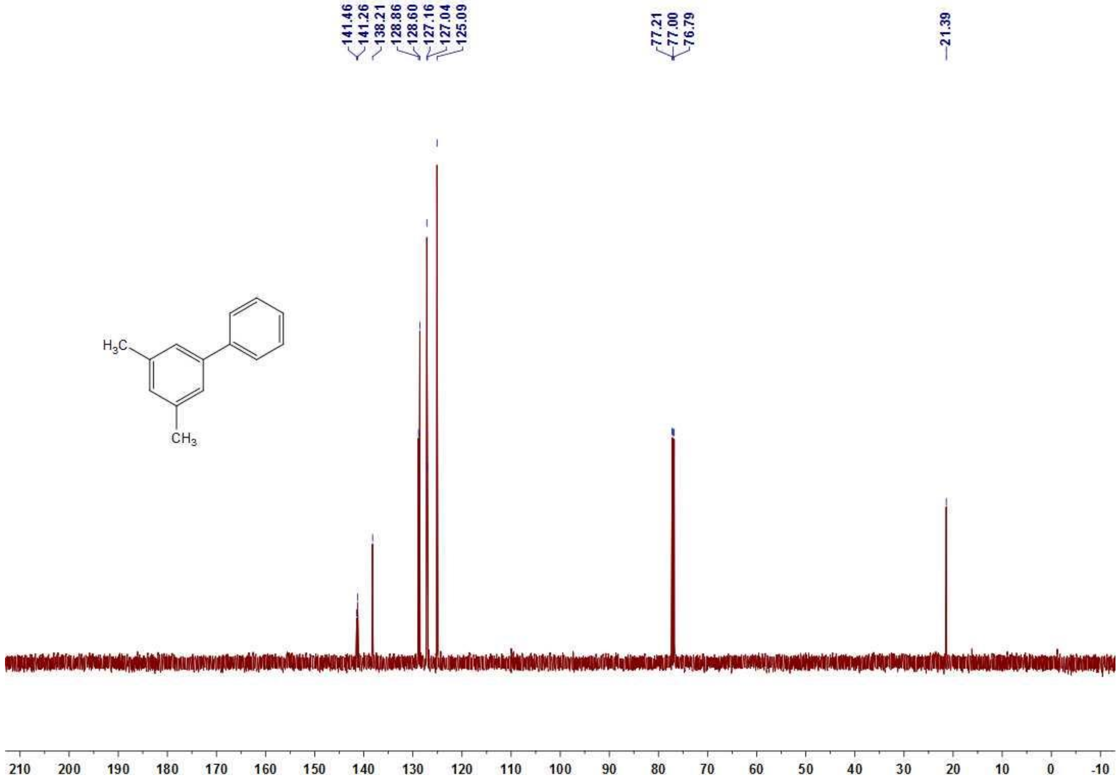

Supplementary Figure 45 ^{13}C NMR of **3,5-dimethyl-1,1'-biphenyl**

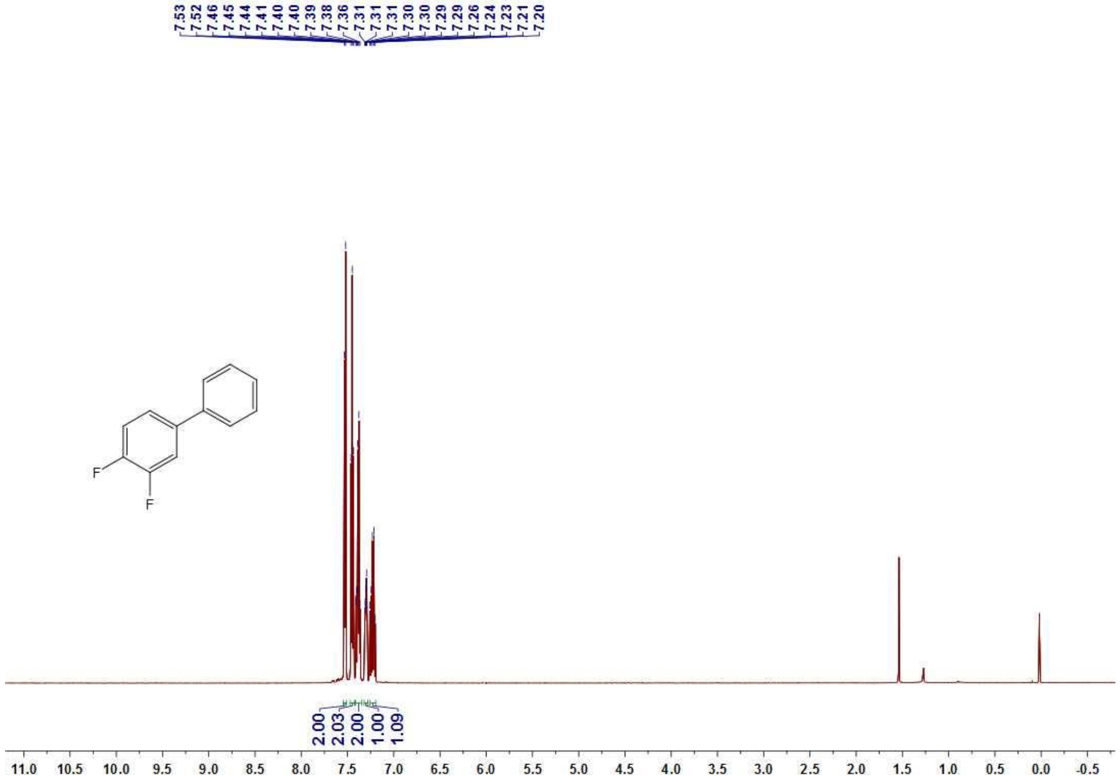

Supplementary Figure 46 ^{1}H NMR of **3,4-difluoro-1,1'-biphenyl**

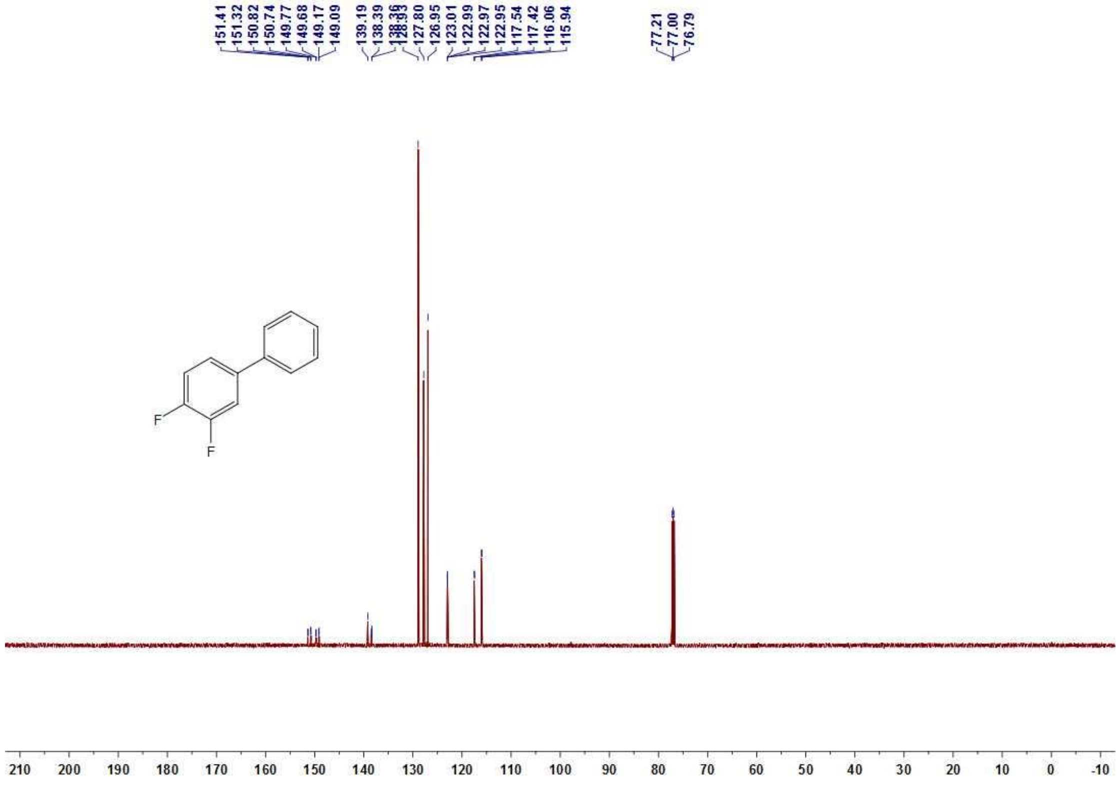

Supplementary Figure 47 ^{13}C NMR of **3,4-difluoro-1,1'-biphenyl**

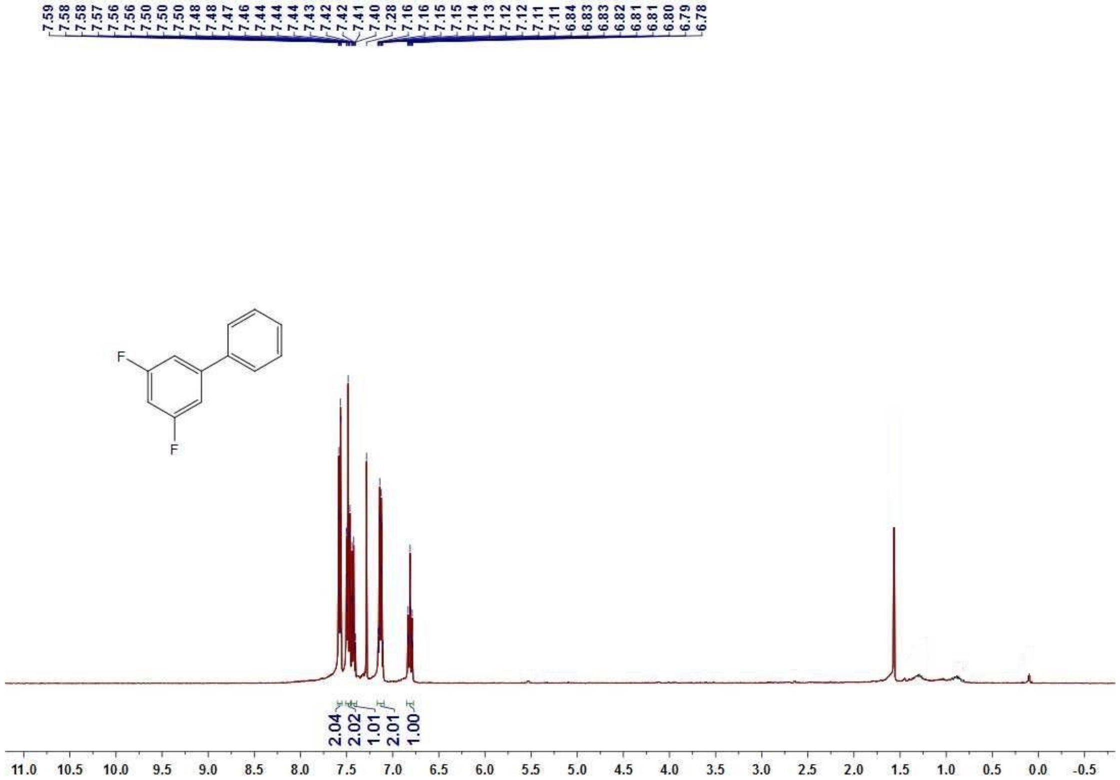

Supplementary Figure 48 ^{1}H NMR of **3,5-difluoro-1,1'-biphenyl**

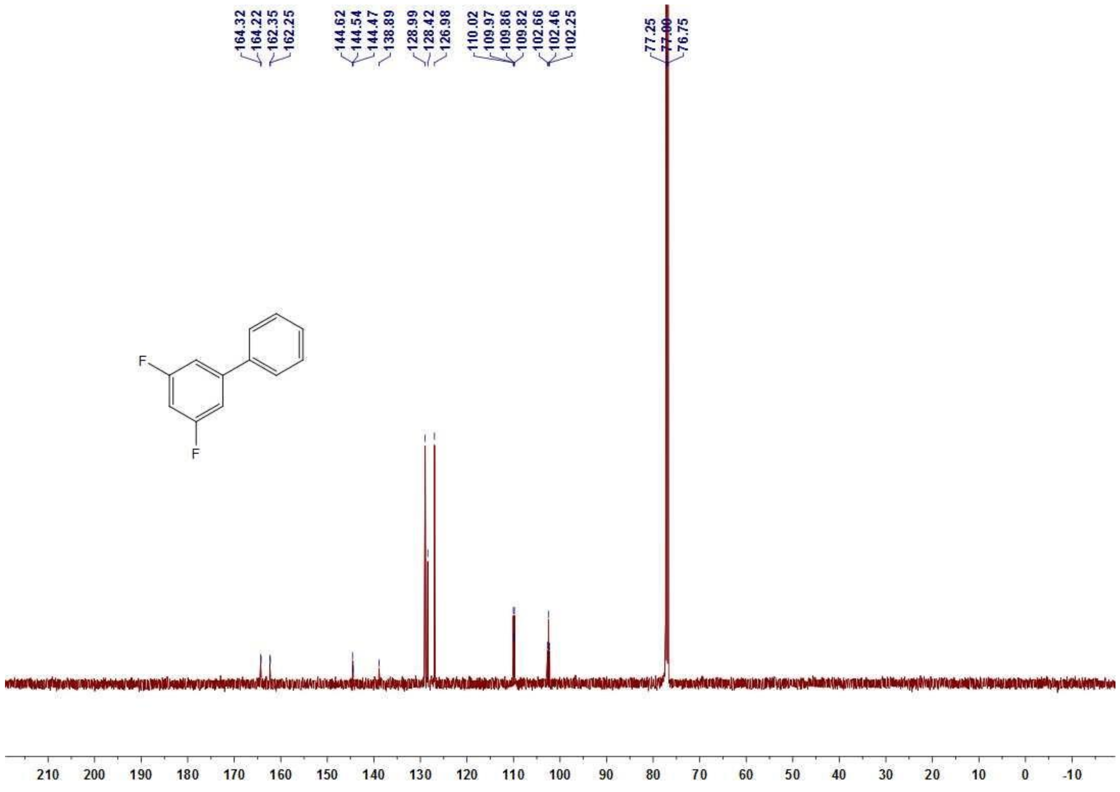

Supplementary Figure 49 ^{13}C NMR of **3,5-difluoro-1,1'-biphenyl**

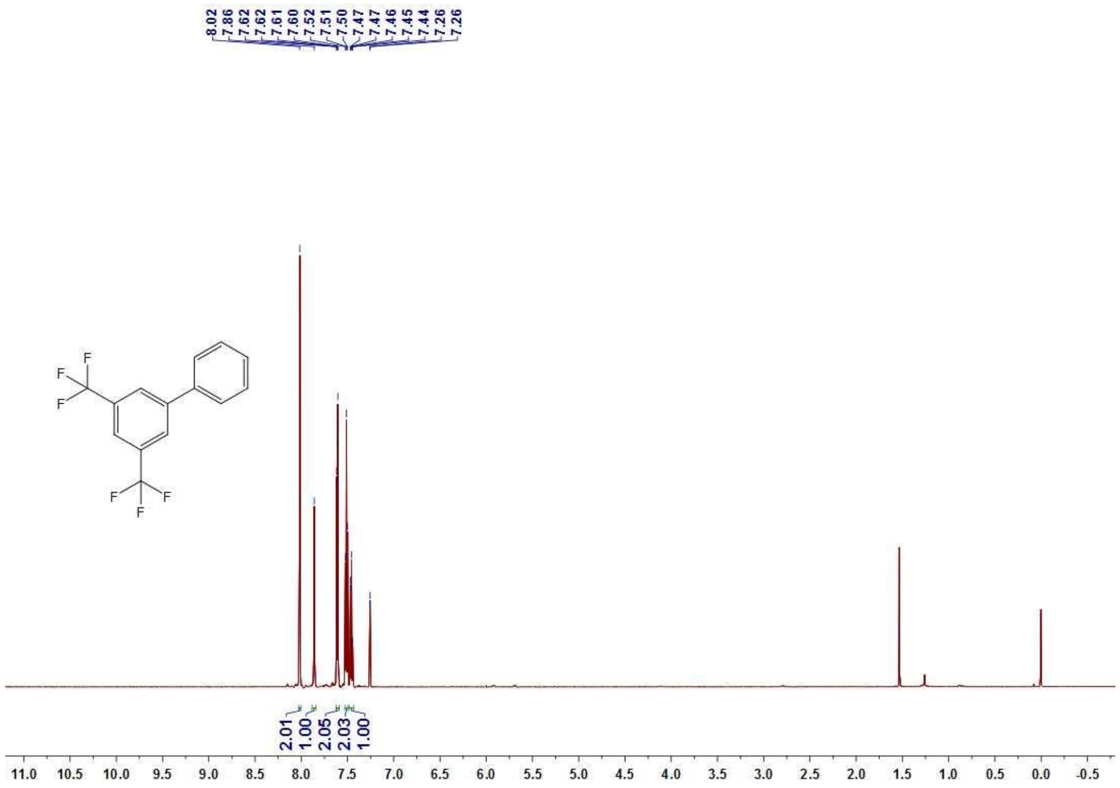

Supplementary Figure 50 ^{1}H NMR of **3,5-bis(trifluoromethyl)-1,1'-biphenyl**

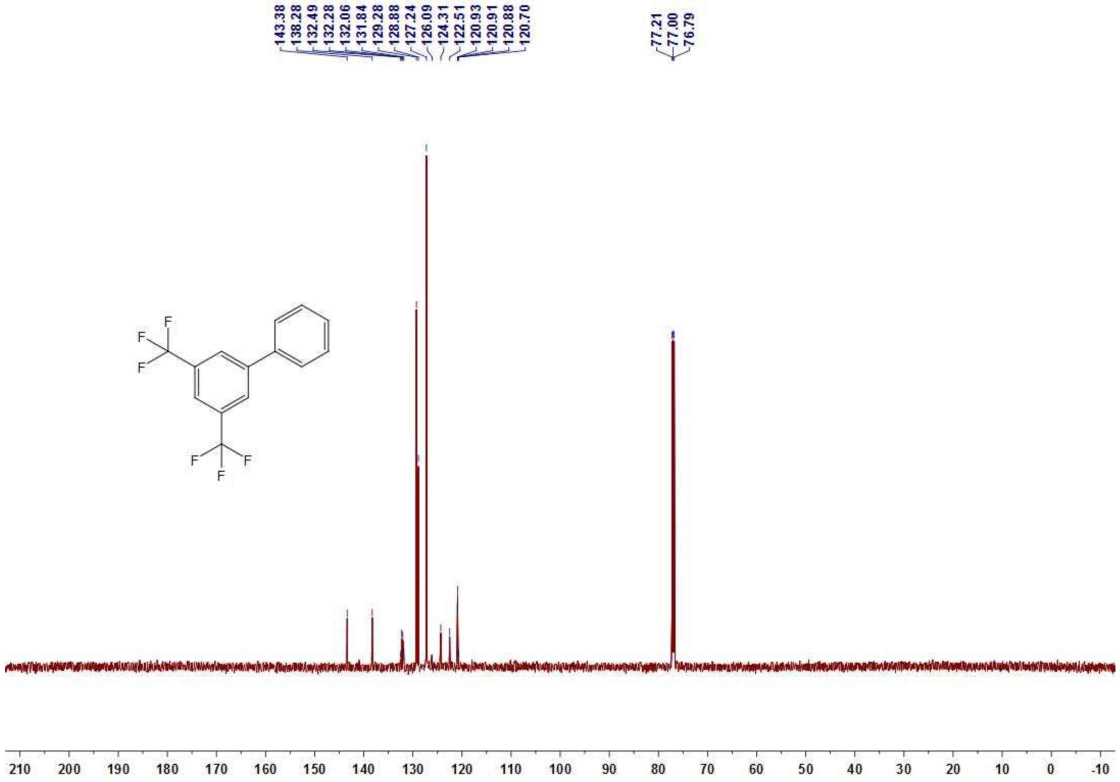

Supplementary Figure 51 ^{13}C NMR of **3,5-bis(trifluoromethyl)-1,1'-biphenyl**

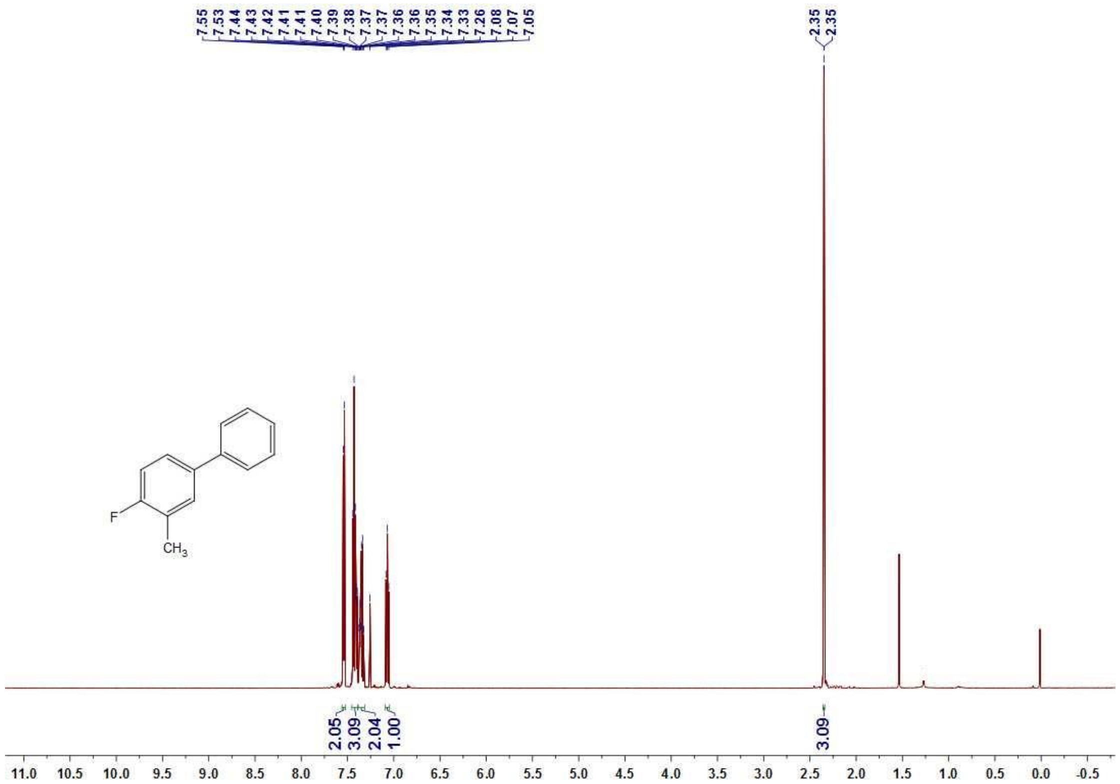

Supplementary Figure 52 ^{1}H NMR of **4-fluoro-3-methyl-1,1'-biphenyl**

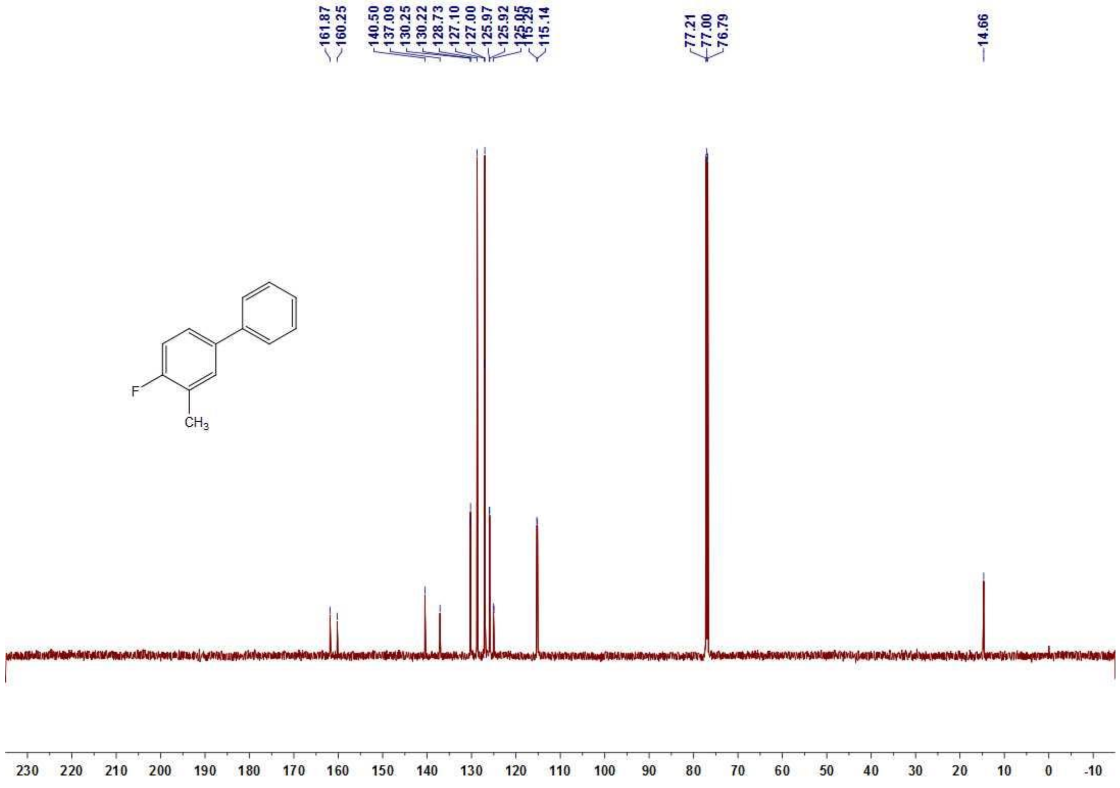

Supplementary Figure 53 ^{13}C NMR of **4-fluoro-3-methyl-1,1'-biphenyl**

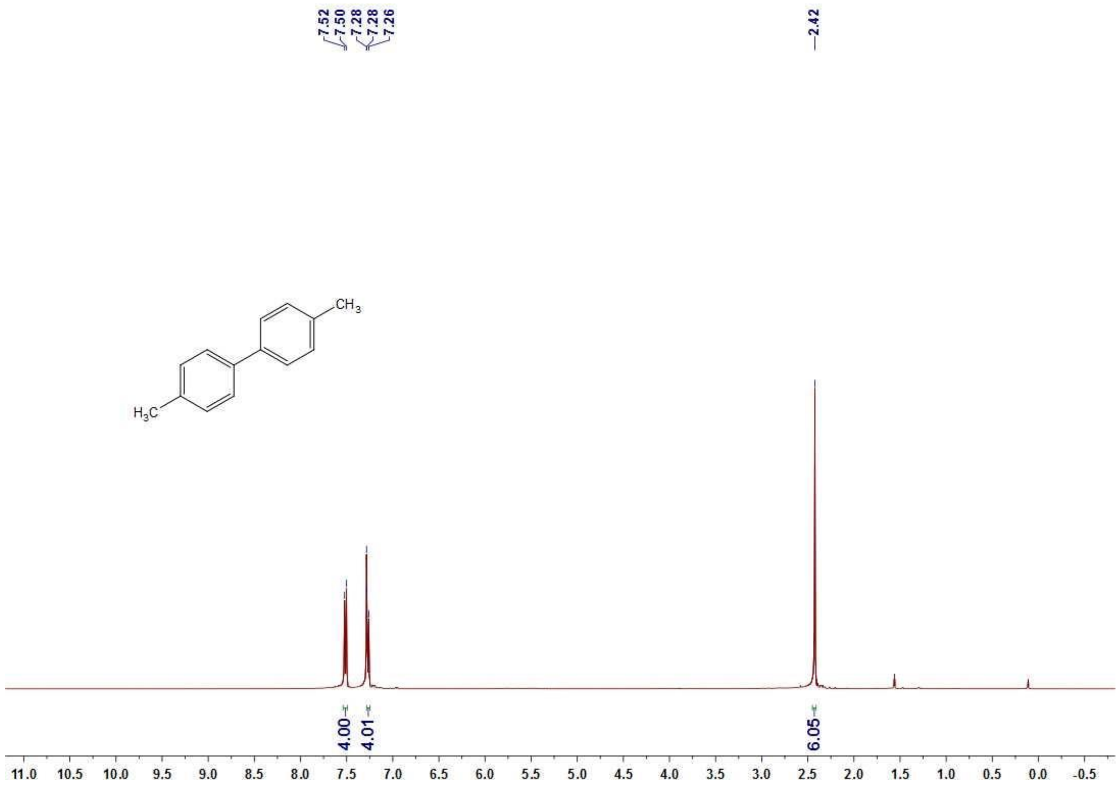

Supplementary Figure 54 ^{1}H NMR of **4,4'-dimethyl-1,1'-biphenyl**

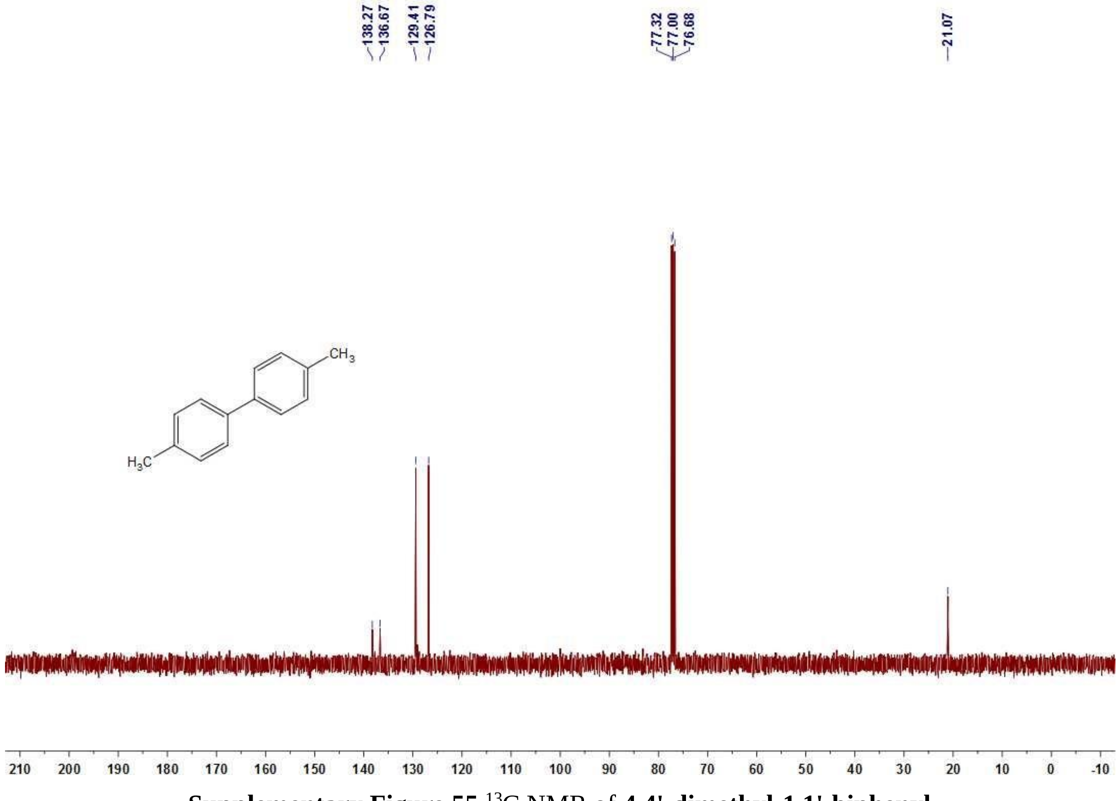

Supplementary Figure 55 ^{13}C NMR of **4,4'-dimethyl-1,1'-biphenyl**

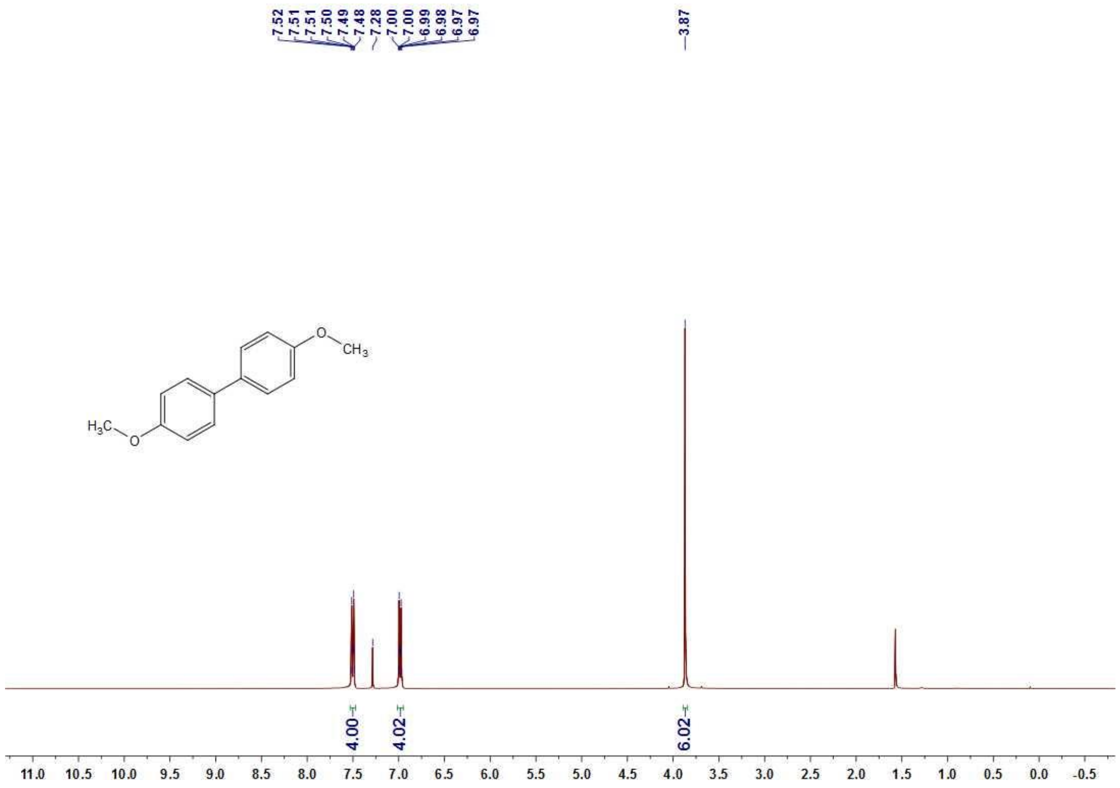

Supplementary Figure 56 ^{1}H NMR of **4,4'-dimethoxy-1,1'-biphenyl**

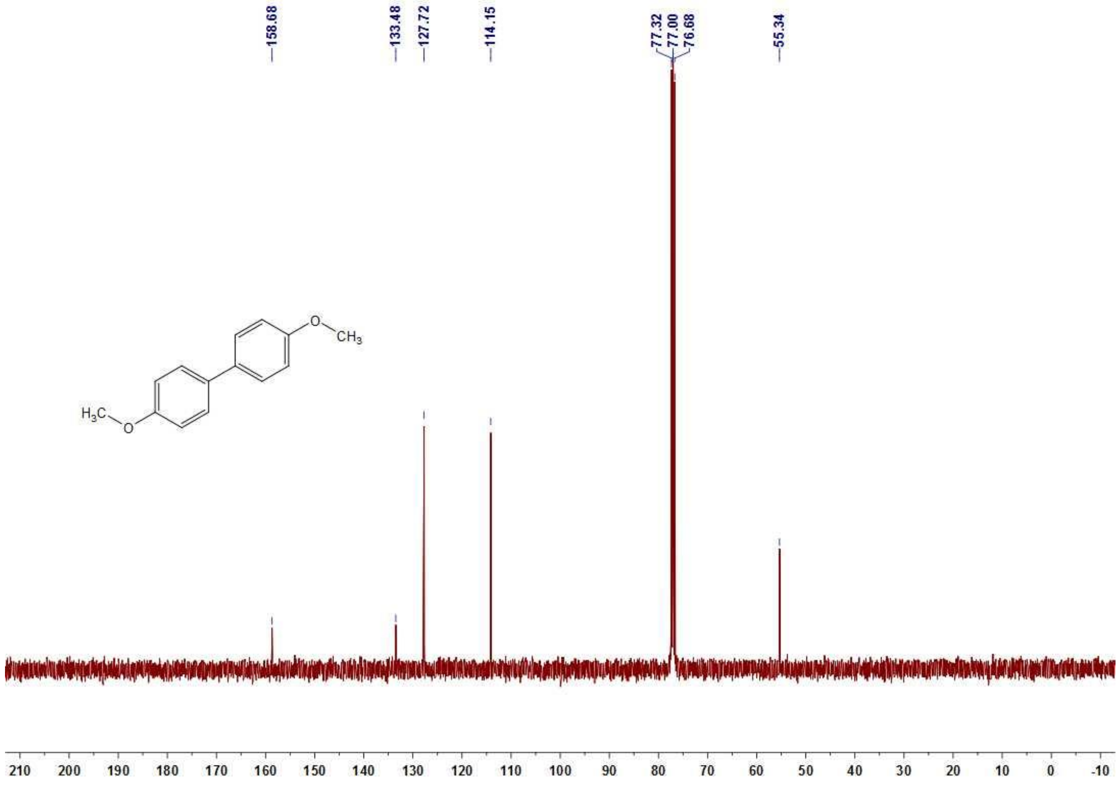

Supplementary Figure 57 ^{13}C NMR of **4,4'-dimethoxy-1,1'-biphenyl**

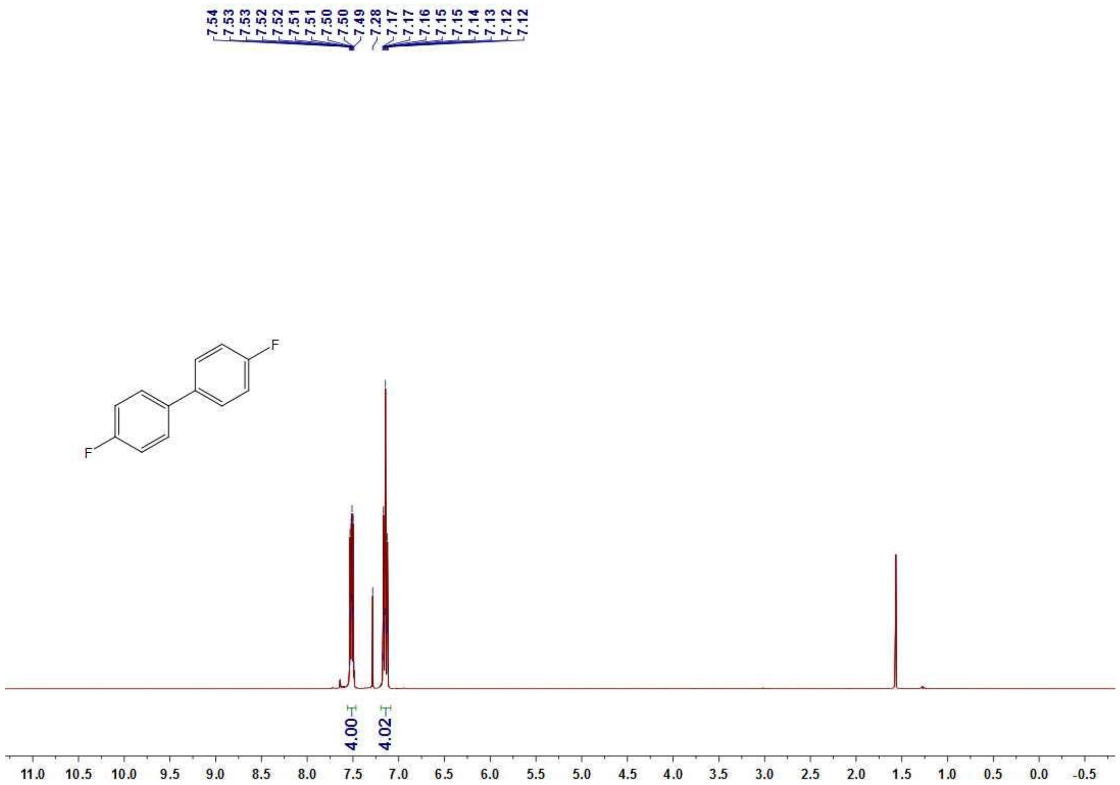

Supplementary Figure 58 ^{1}H NMR of **4,4'-difluoro-1,1'-biphenyl**

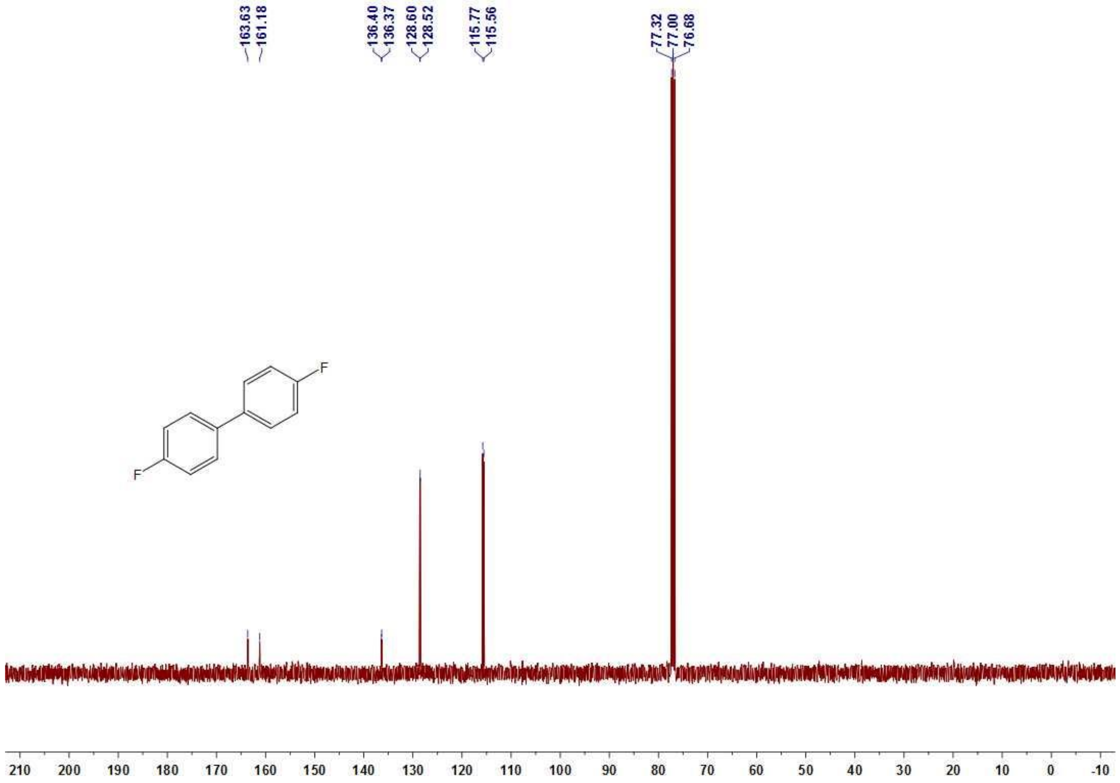

Supplementary Figure 59 ^{13}C NMR of **4,4'-difluoro-1,1'-biphenyl**

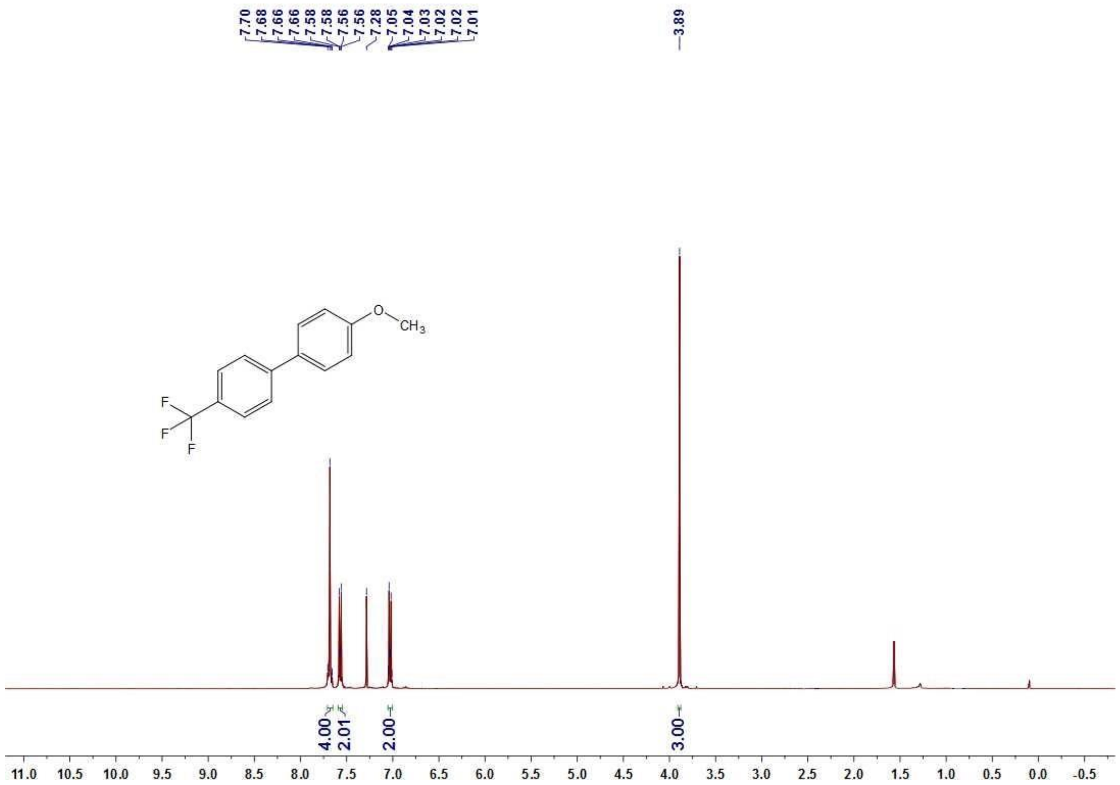

Supplementary Figure 60 ^{1}H NMR of **4-methoxy-4'-(trifluoromethyl)-1,1'-biphenyl**

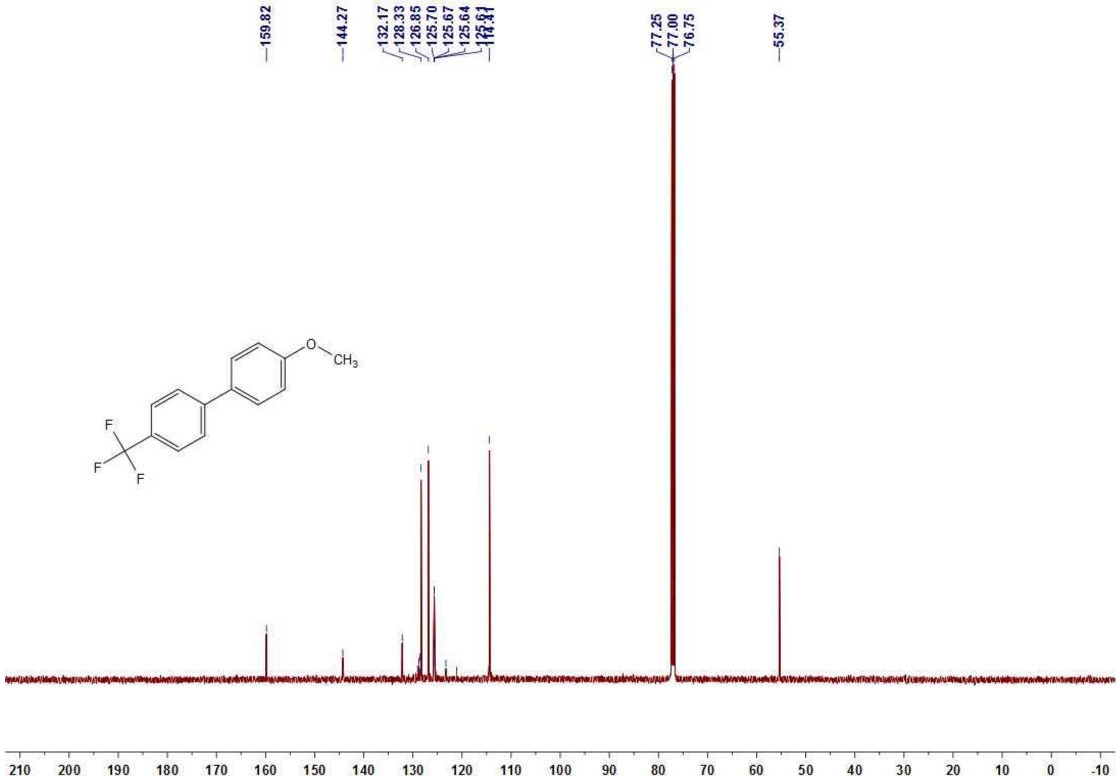

Supplementary Figure 61 ^{13}C NMR of **4-methoxy-4'-(trifluoromethyl)-1,1'-biphenyl**

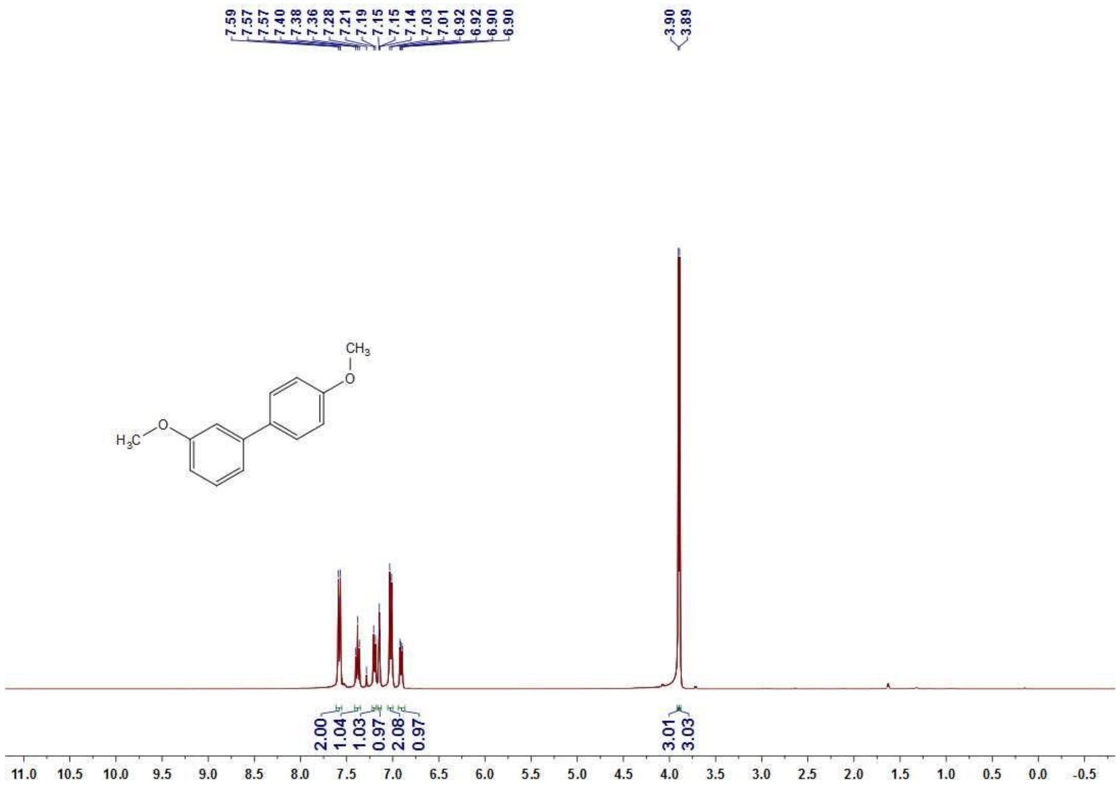

Supplementary Figure 62 ^{1}H NMR of **3,4'-dimethoxy-1,1'-biphenyl**

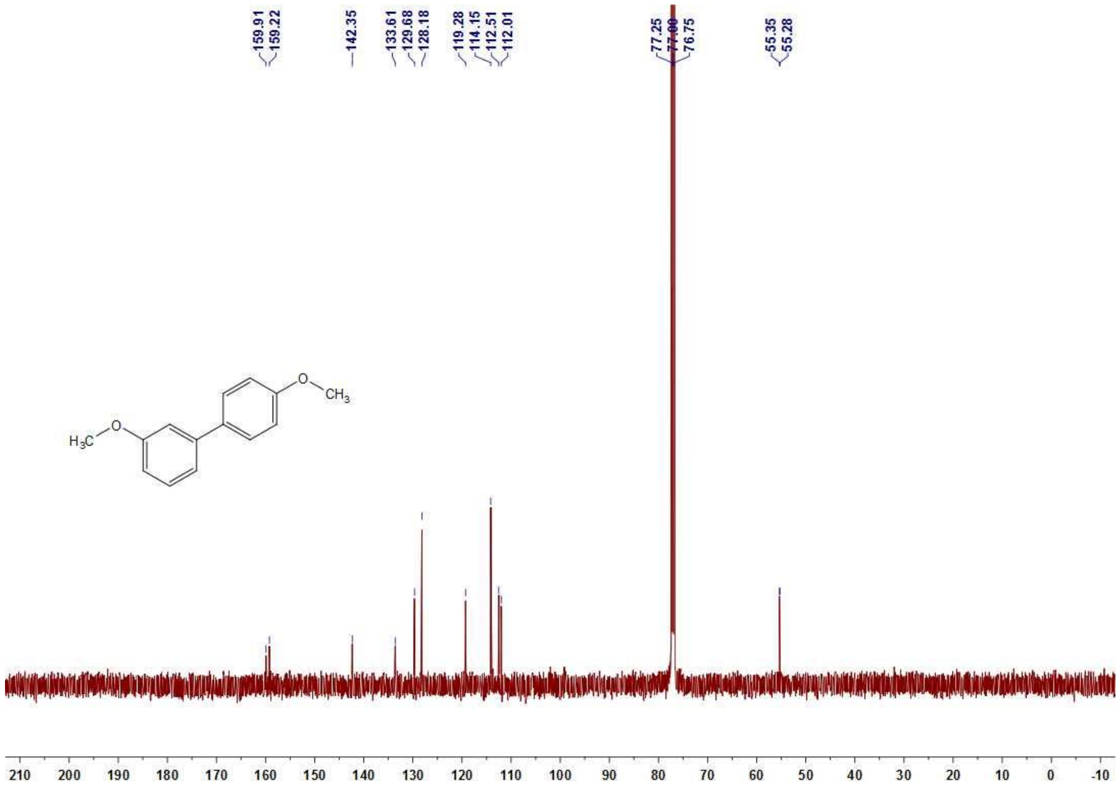

Supplementary Figure 63 ^{13}C NMR of **3,4'-dimethoxy-1,1'-biphenyl**

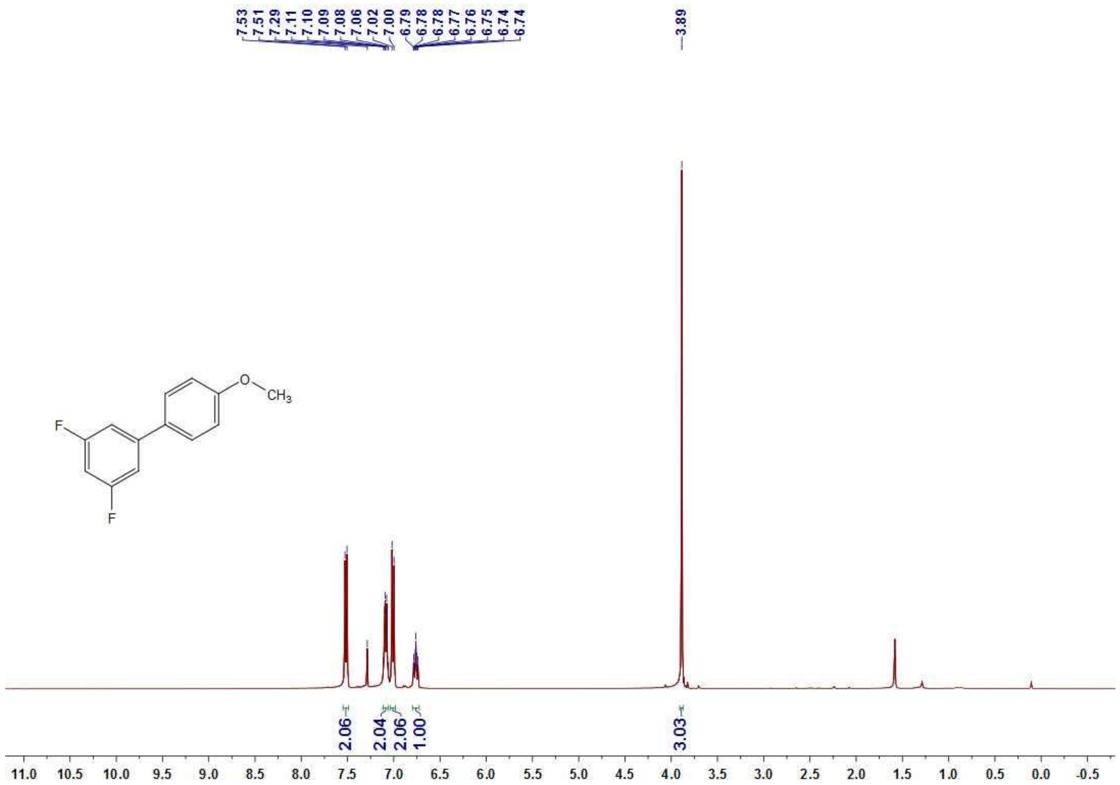

Supplementary Figure 64 ^{1}H NMR of **3,5-difluoro-4'-methoxy-1,1'-biphenyl**

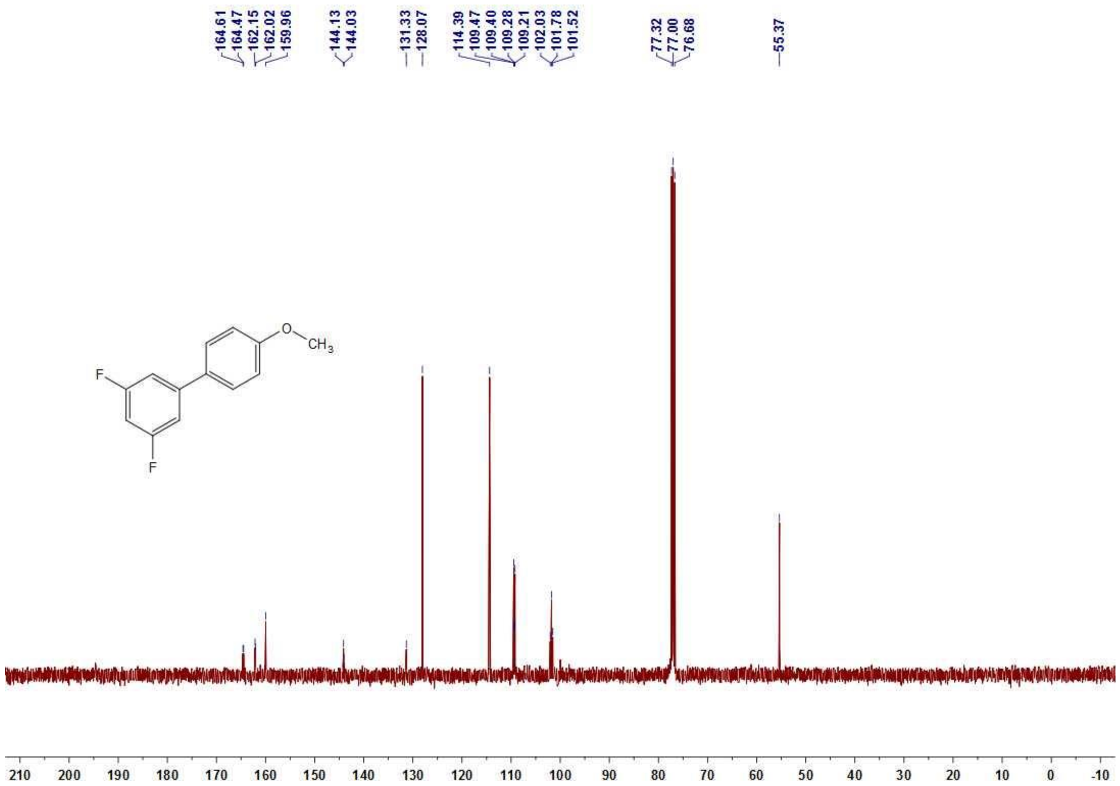

Supplementary Figure 65 ^{13}C NMR of **3,5-difluoro-4'-methoxy-1,1'-biphenyl**

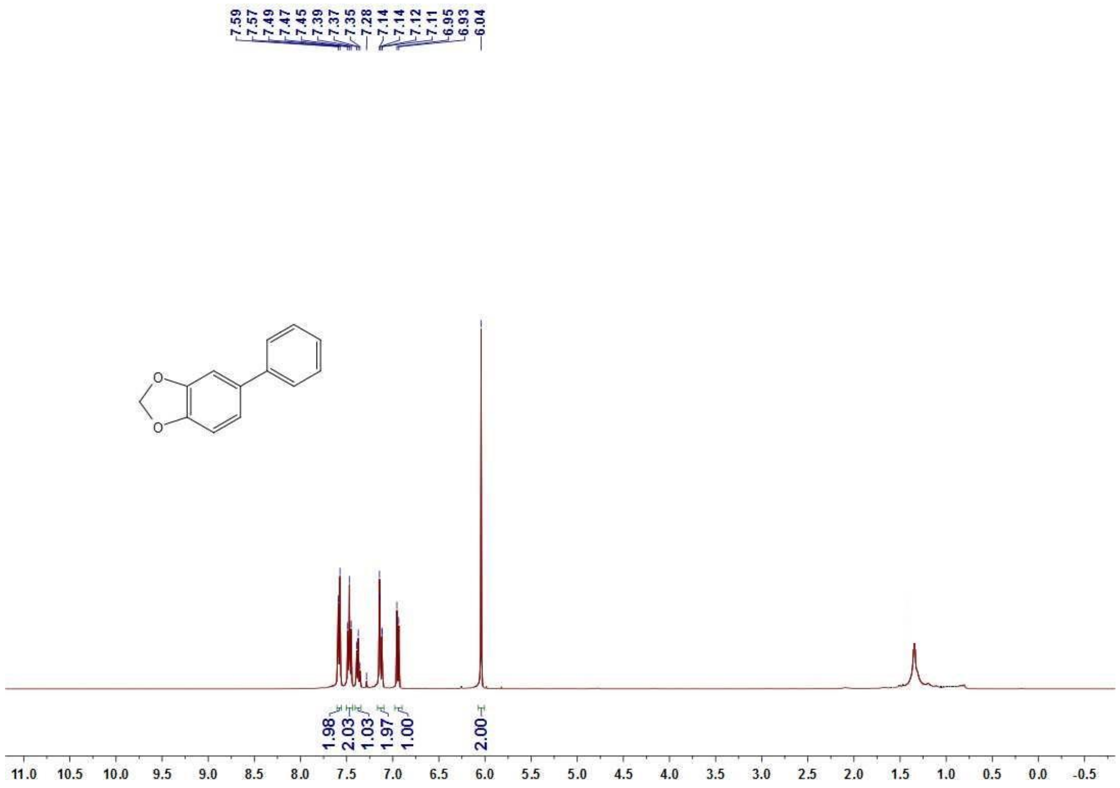

Supplementary Figure 66 ^{1}H NMR of **3,4-methylenedioxybiphenyl**

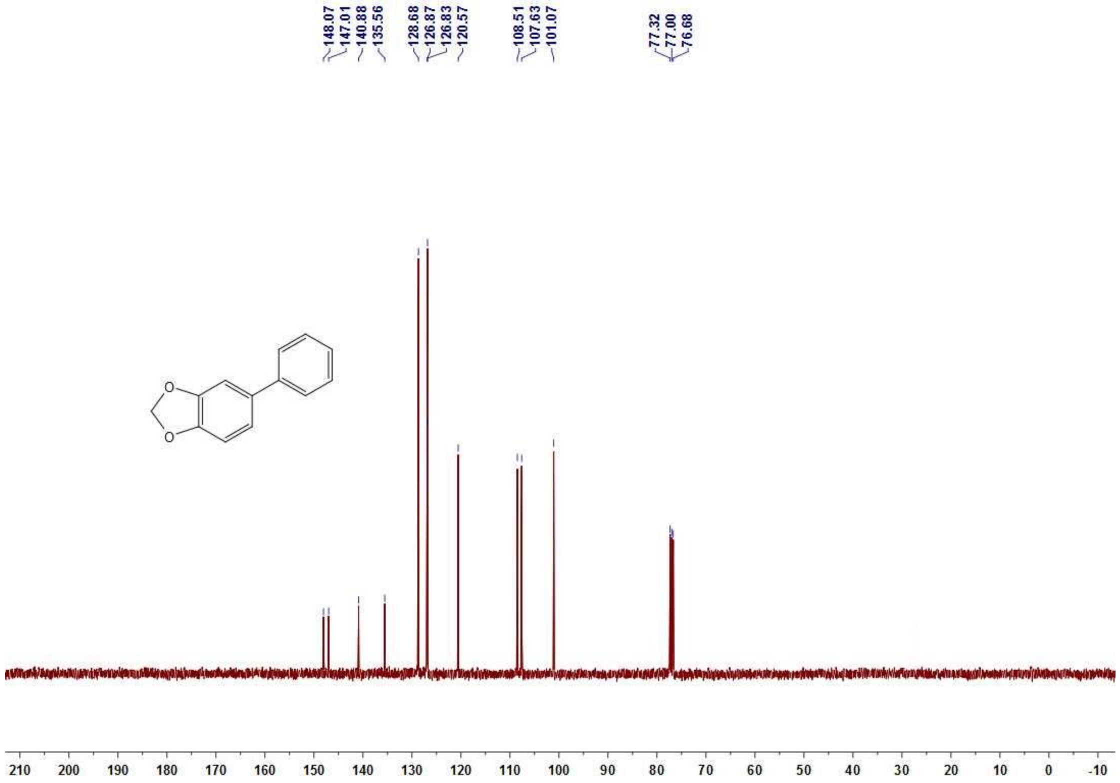

Supplementary Figure 67 ^{13}C NMR of **3,4-methylenedioxybiphenyl**

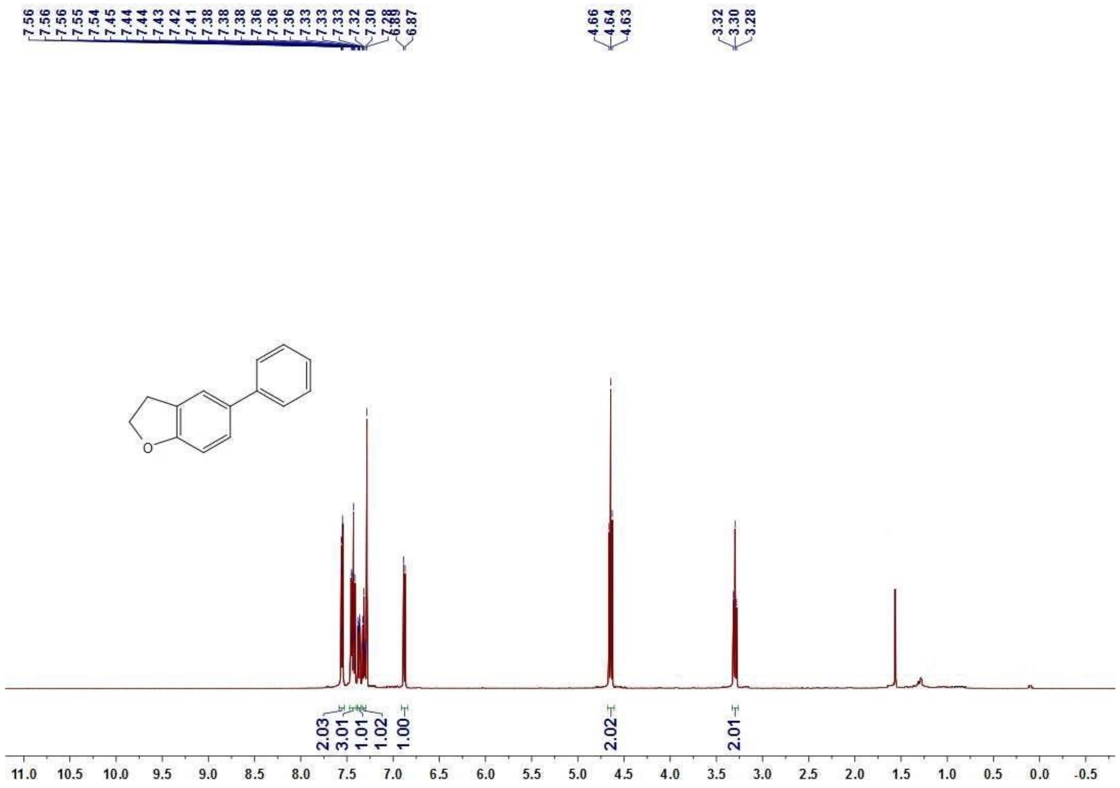

Supplementary Figure 68 ^{1}H NMR of **5-phenyl-2,3-dihydrobenzofuran**

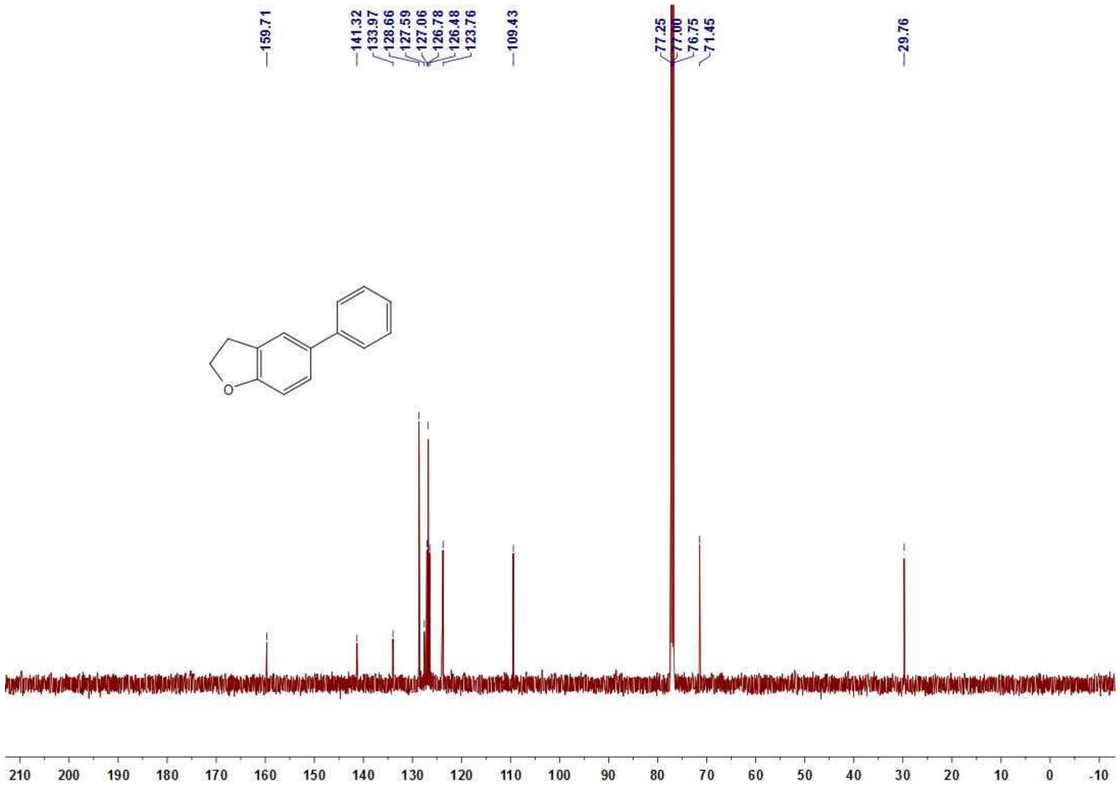

Supplementary Figure 69 ^{13}C NMR of **5-phenyl-2,3-dihydrobenzofuran**

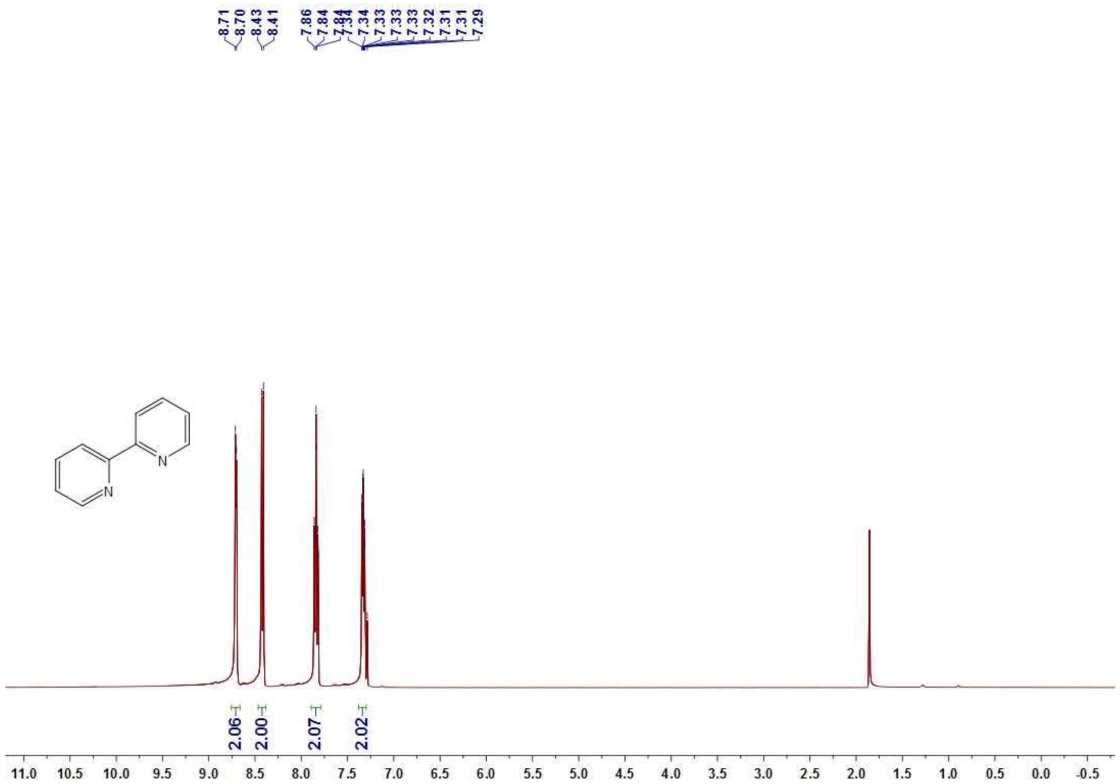

Supplementary Figure 70 ^{1}H NMR of **2,2'-bipyridine**

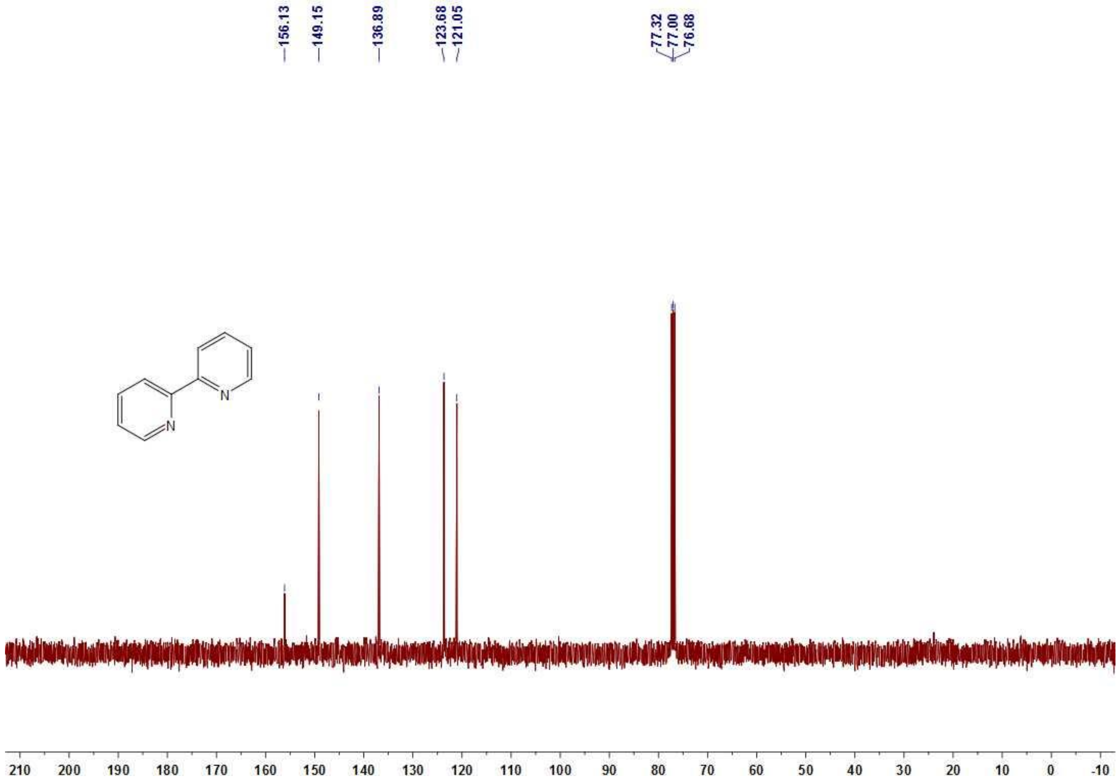

Supplementary Figure 71 ^{13}C NMR of **2,2'-bipyridine**

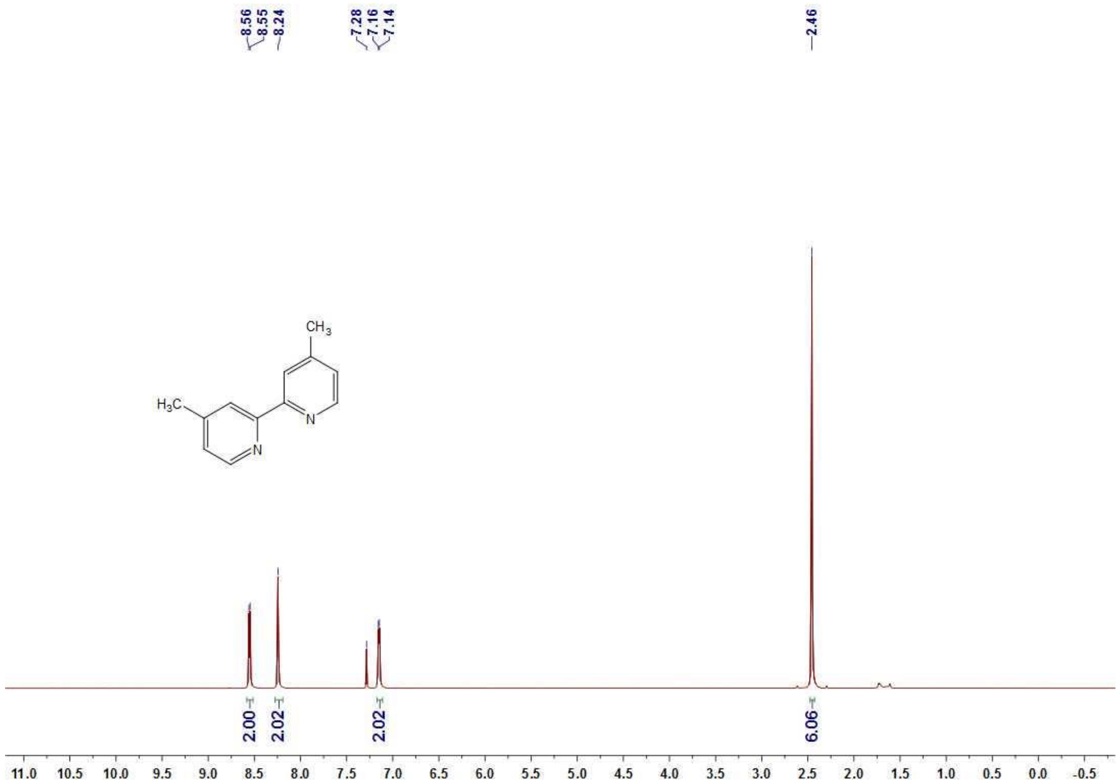

Supplementary Figure 72 ^{1}H NMR of **4,4'-dimethyl-2,2'-bipyridine**

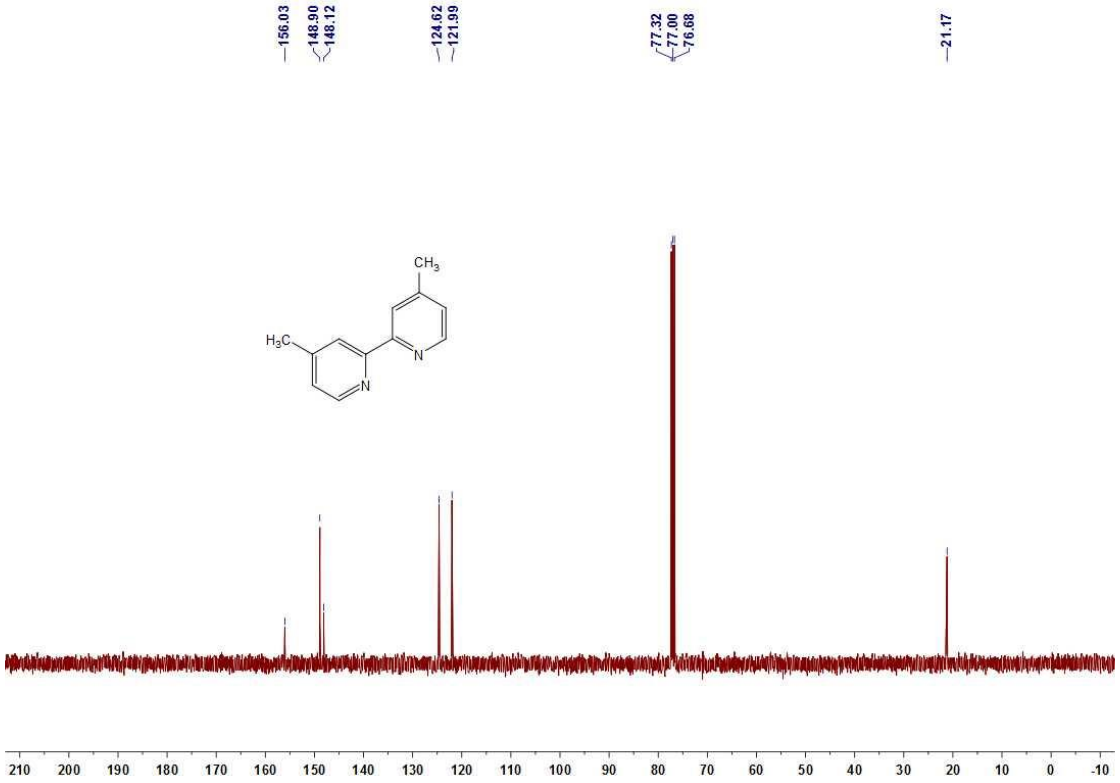

Supplementary Figure 73 ^{13}C NMR of **4,4'-dimethyl-2,2'-bipyridine**

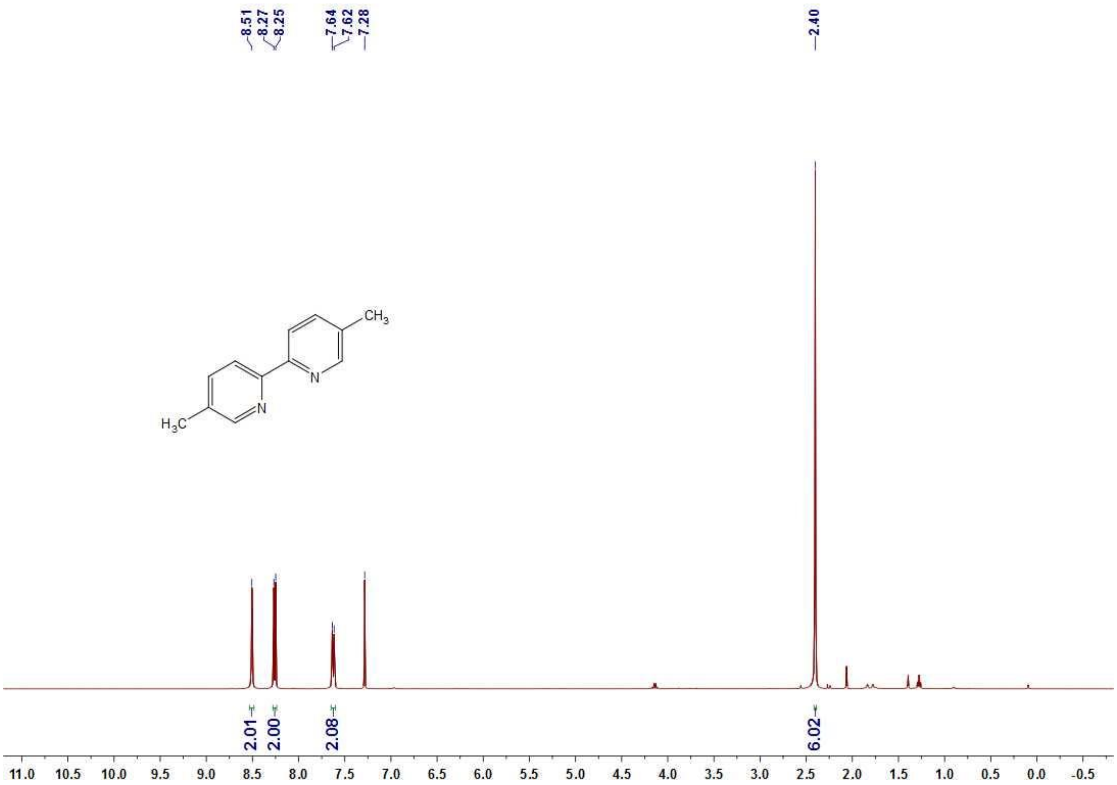

Supplementary Figure 74 ^{1}H NMR of **5,5'-dimethyl-2,2'-bipyridine**

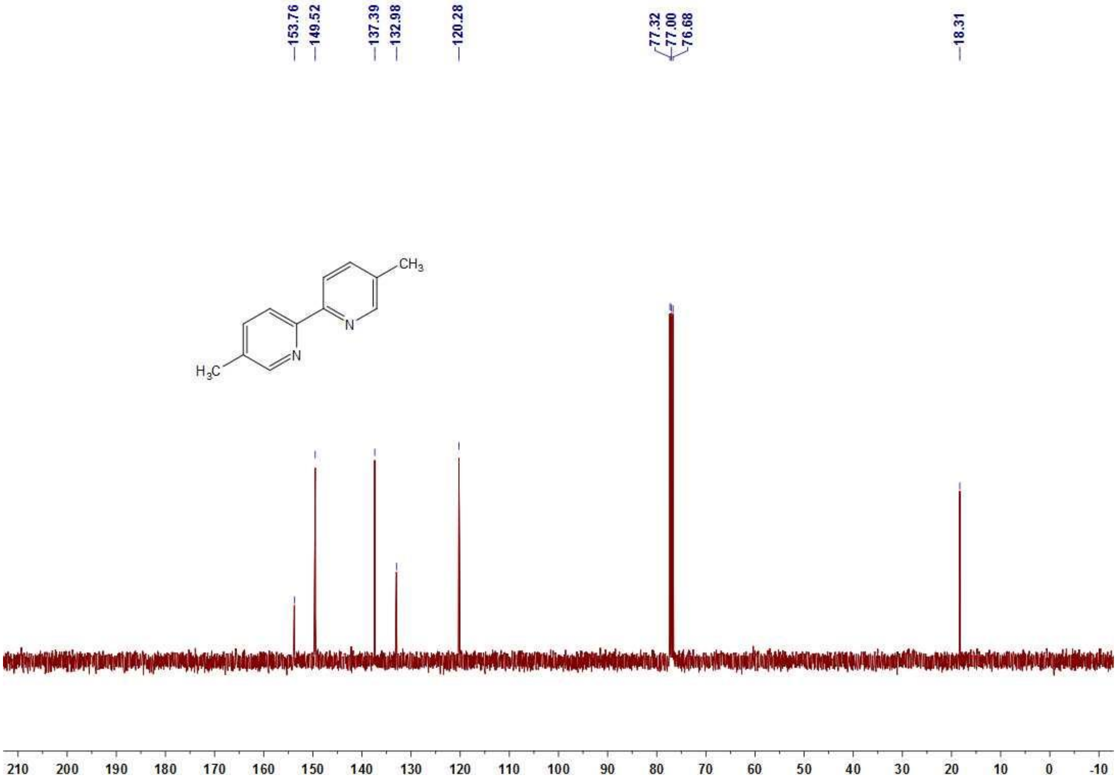

Supplementary Figure 75 ^{13}C NMR of **5,5'-dimethyl-2,2'-bipyridine**

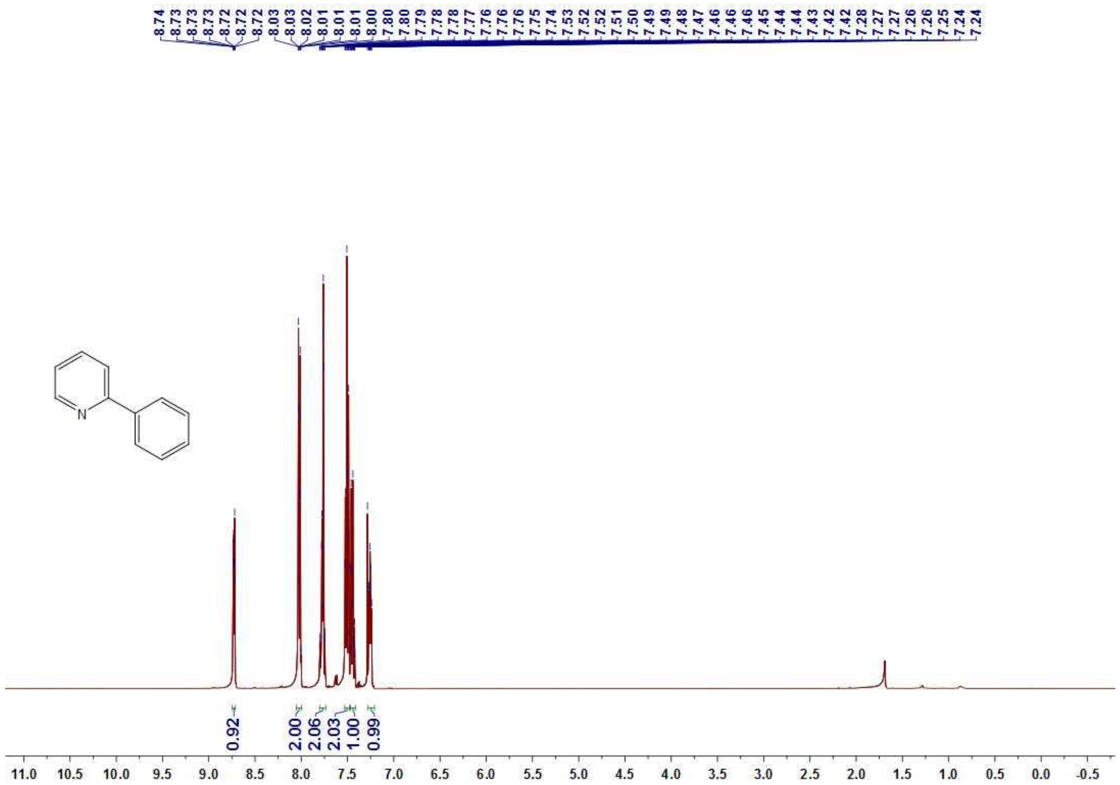

Supplementary Figure 76 ^{1}H NMR of **2-phenylpyridine**

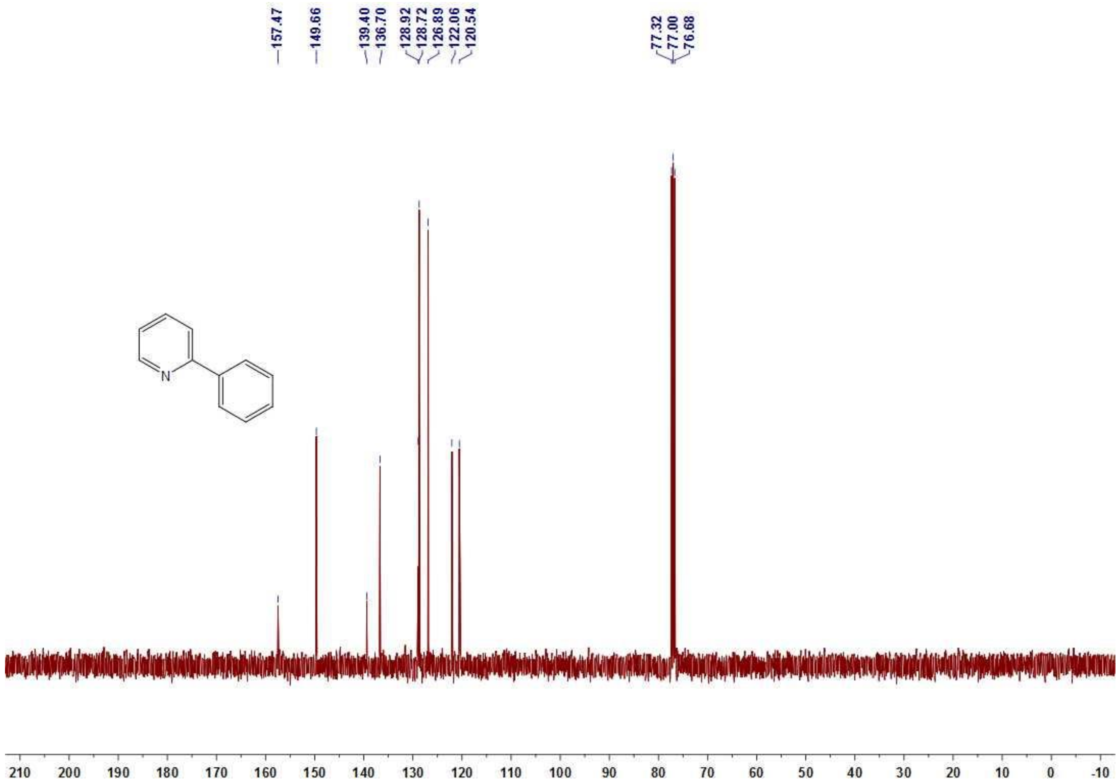

Supplementary Figure 77 ^{13}C NMR of **2-phenylpyridine**

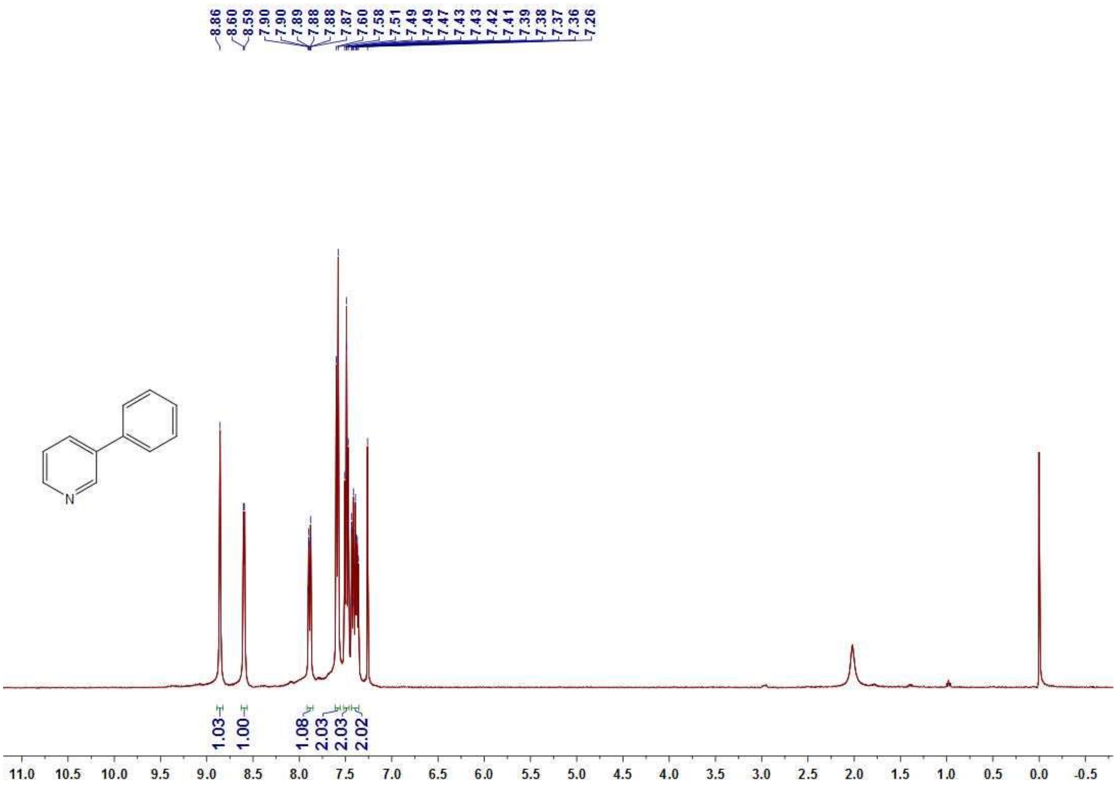

Supplementary Figure 78 ^{1}H NMR of **3-phenylpyridine**

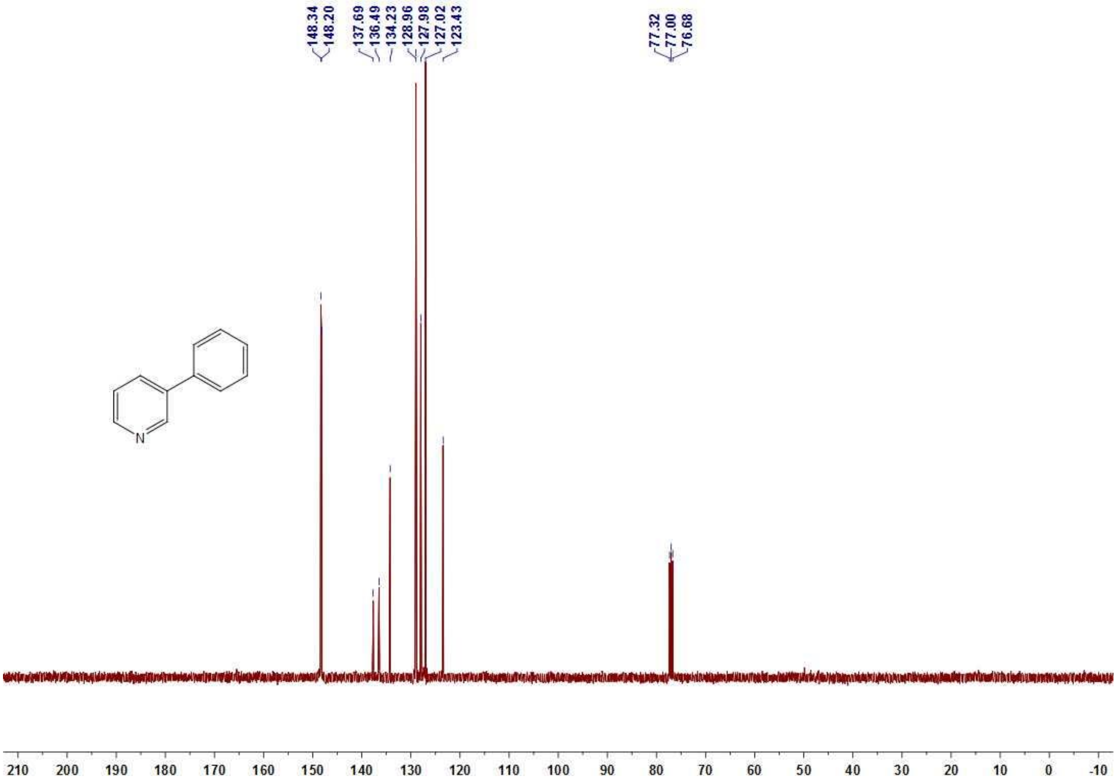

Supplementary Figure 79 ^{13}C NMR of **3-phenylpyridine**

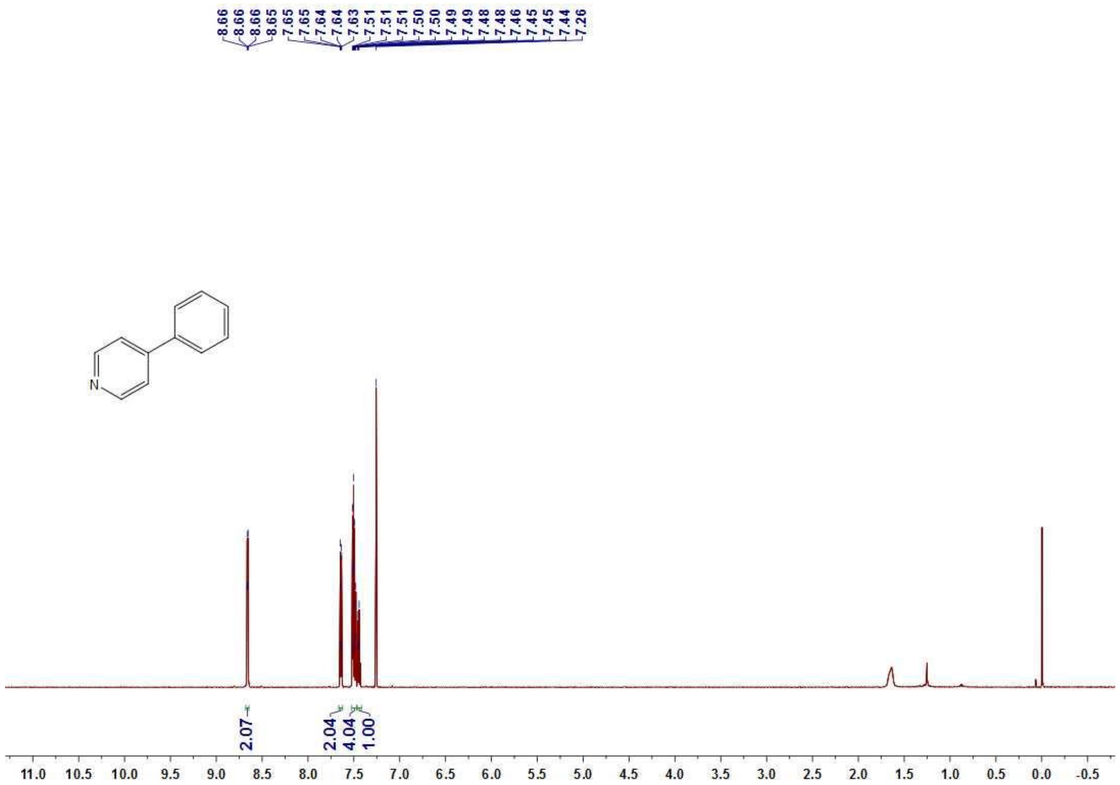

Supplementary Figure 80 ^{1}H NMR of **4-phenylpyridine**

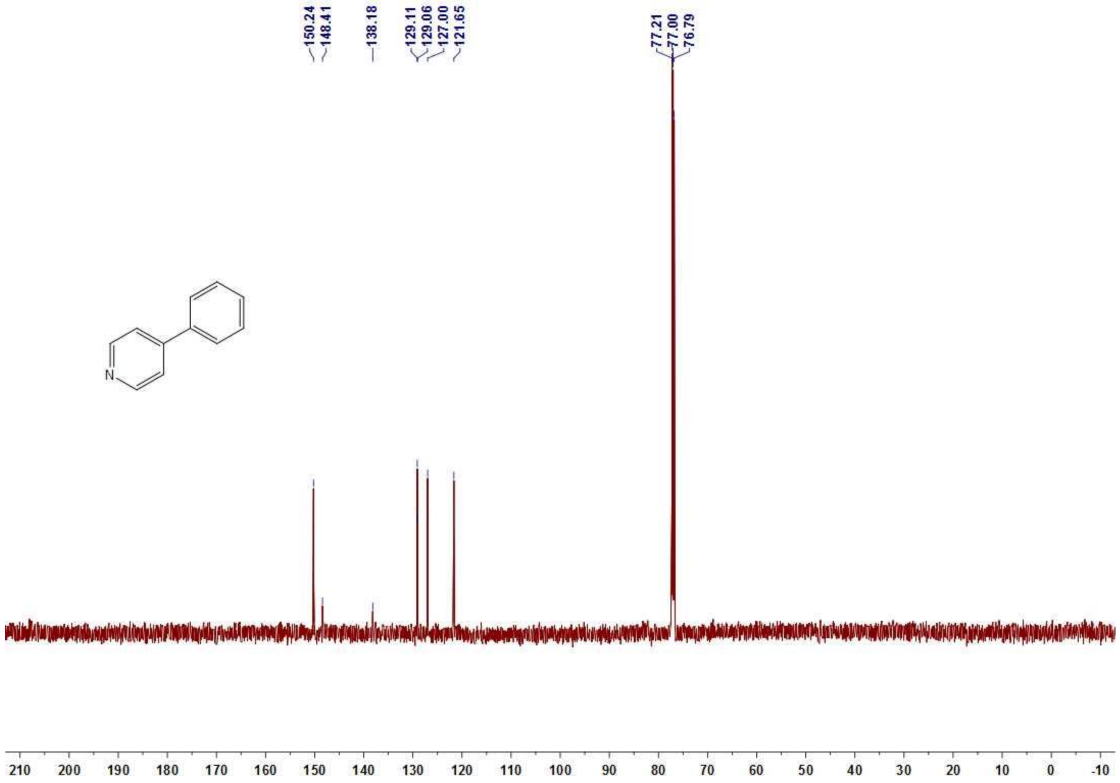

Supplementary Figure 81 ^{13}C NMR of **4-phenylpyridine**

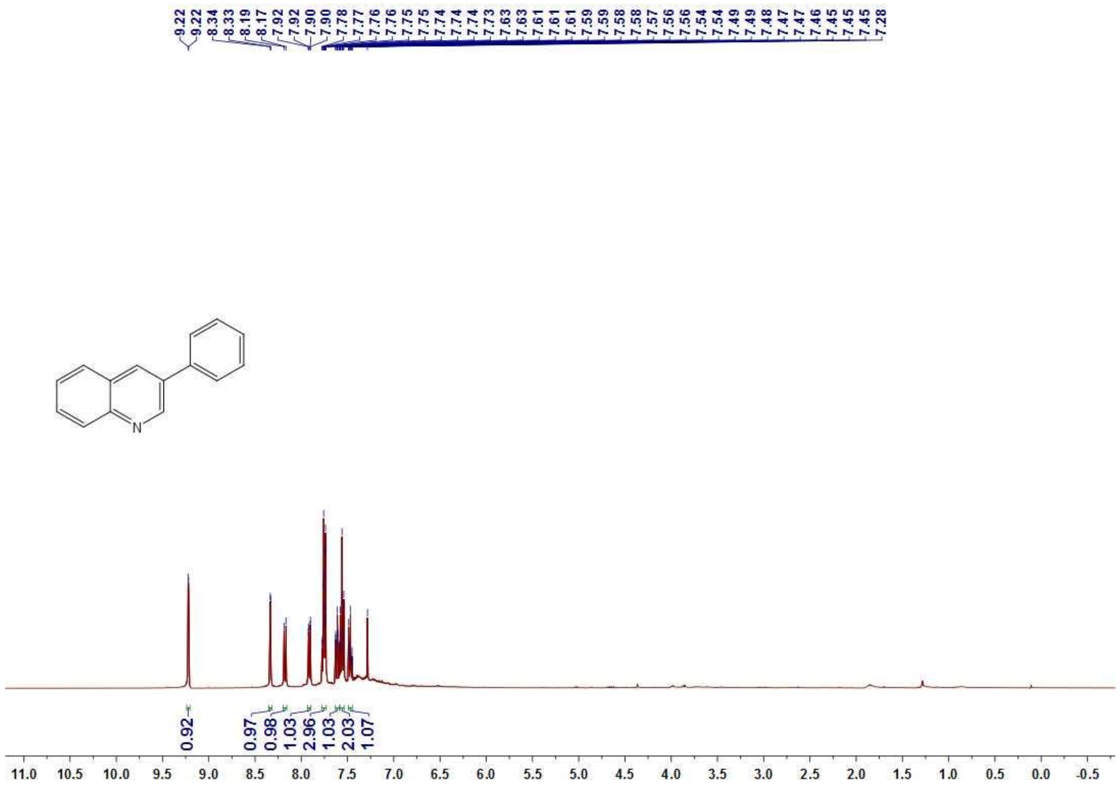

Supplementary Figure 82 ^{1}H NMR of **3-phenylquinoline**

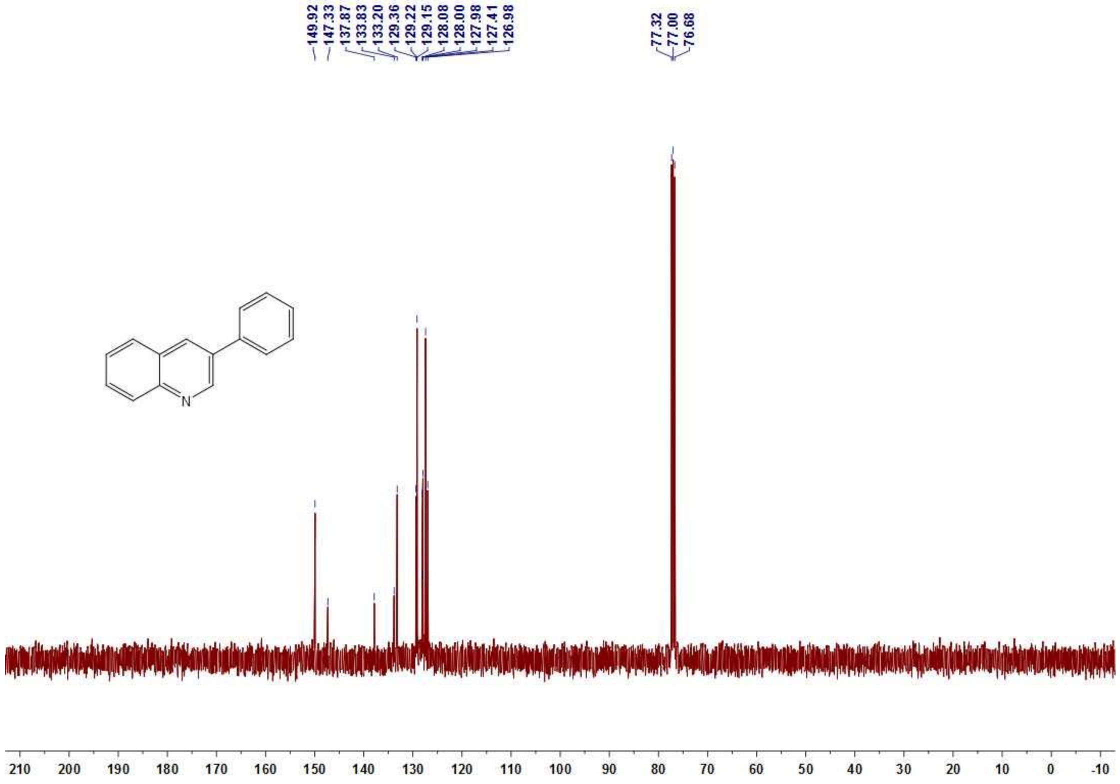

Supplementary Figure 83 ^{13}C NMR of **3-phenylquinoline**

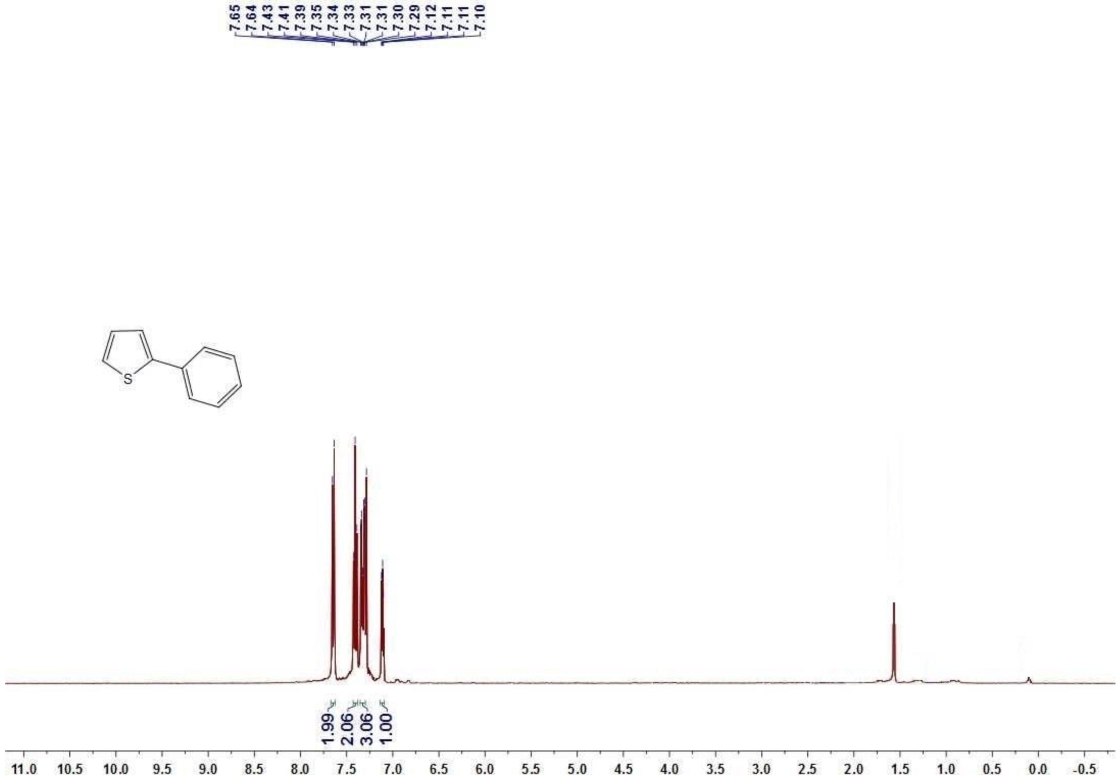

Supplementary Figure 84 ^{1}H NMR of **2-phenylthiophene**

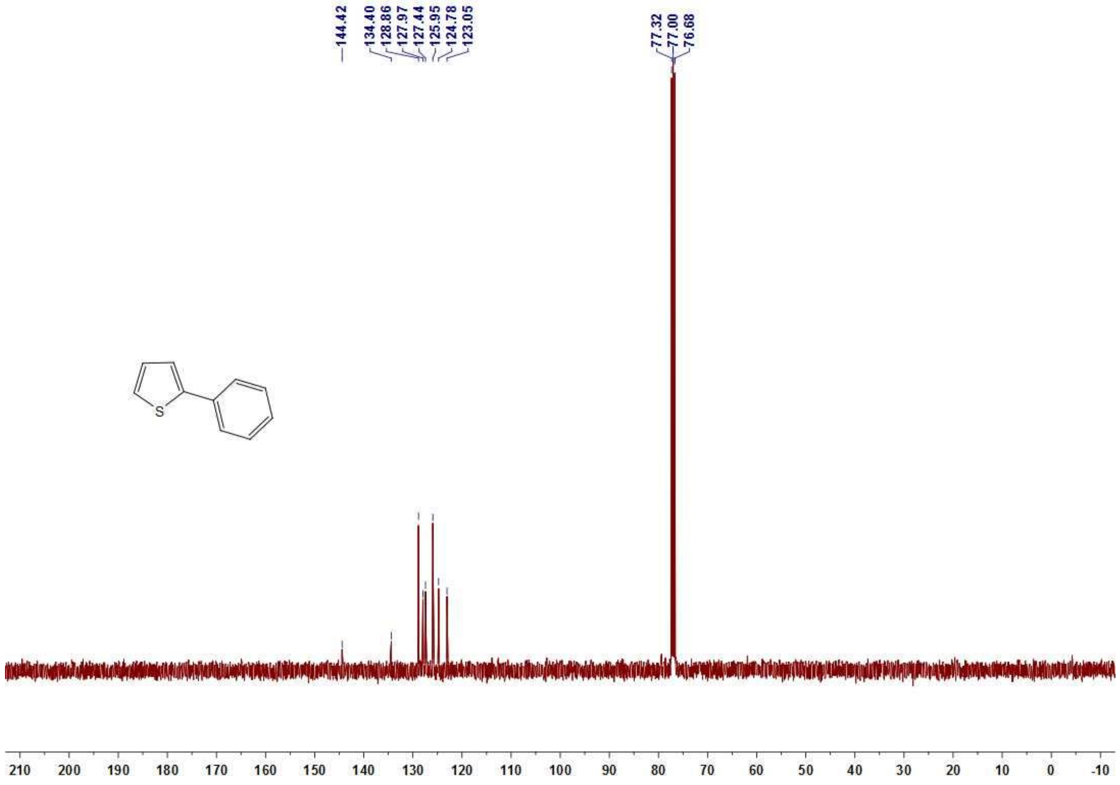

Supplementary Figure 85 ^{13}C NMR of **2-phenylthiophene**

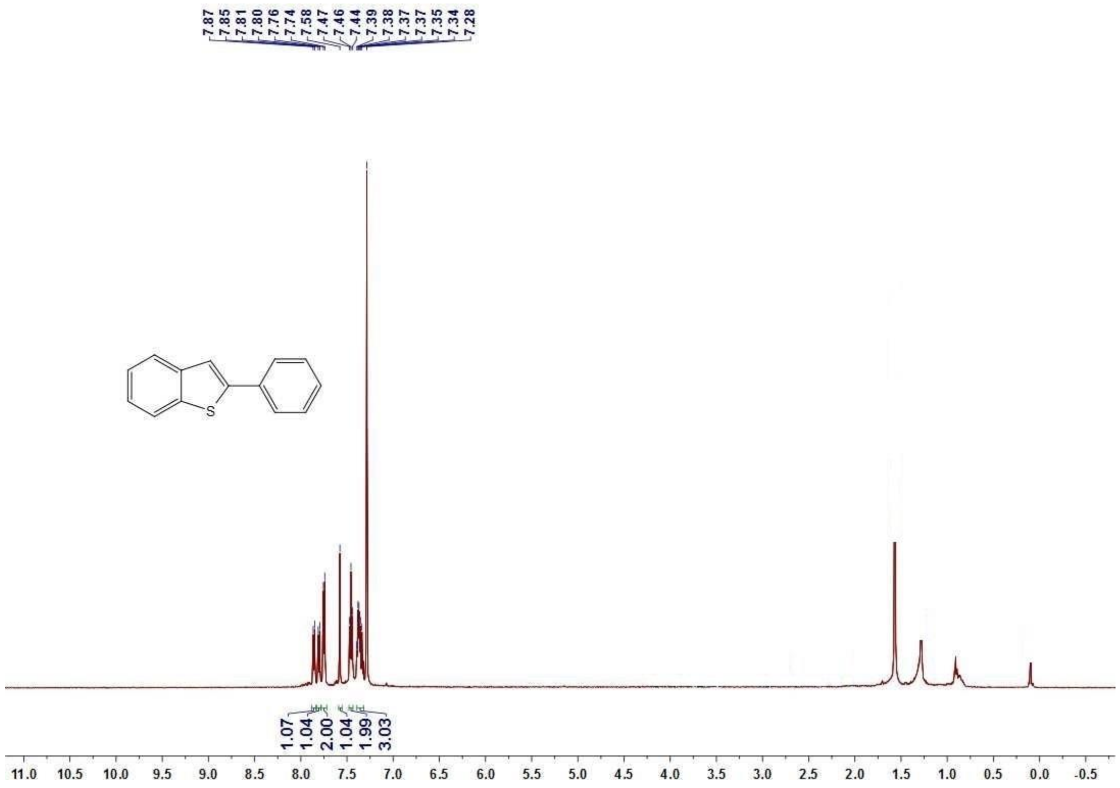

Supplementary Figure 86 ^{1}H NMR of **2-phenylbenzo[b]thiophene**

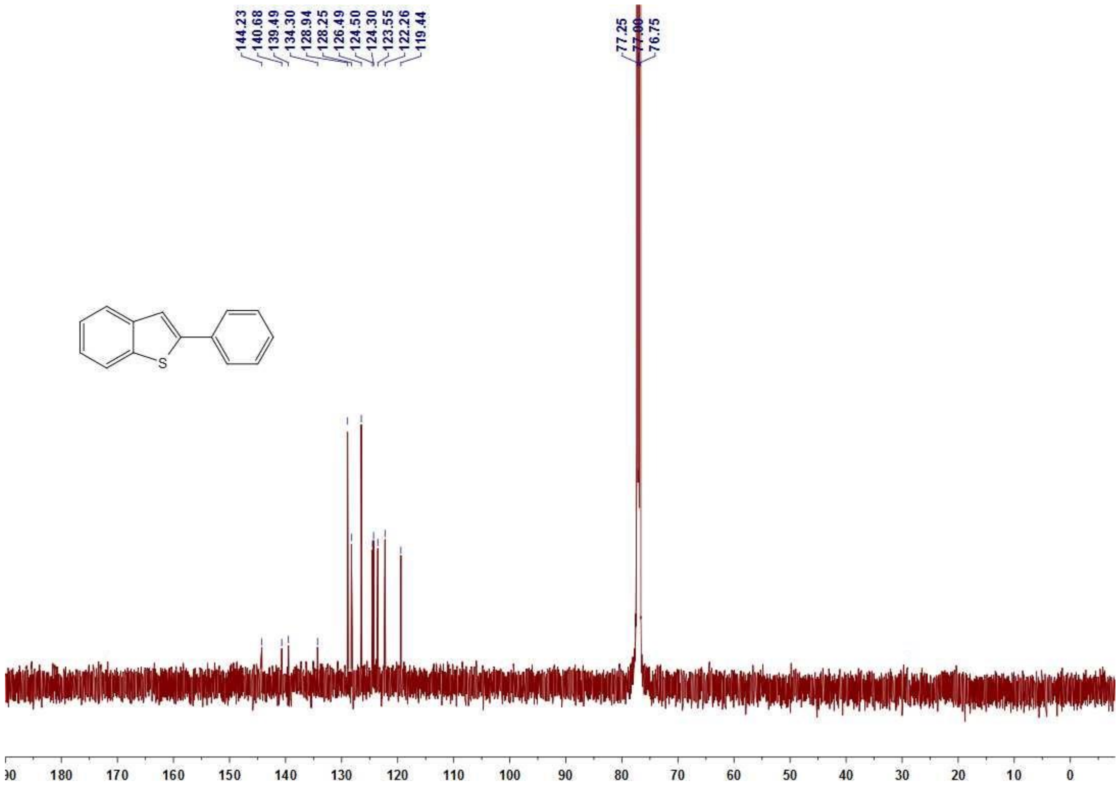

Supplementary Figure 87 ^{13}C NMR of **2-phenylbenzo[b]thiophene**

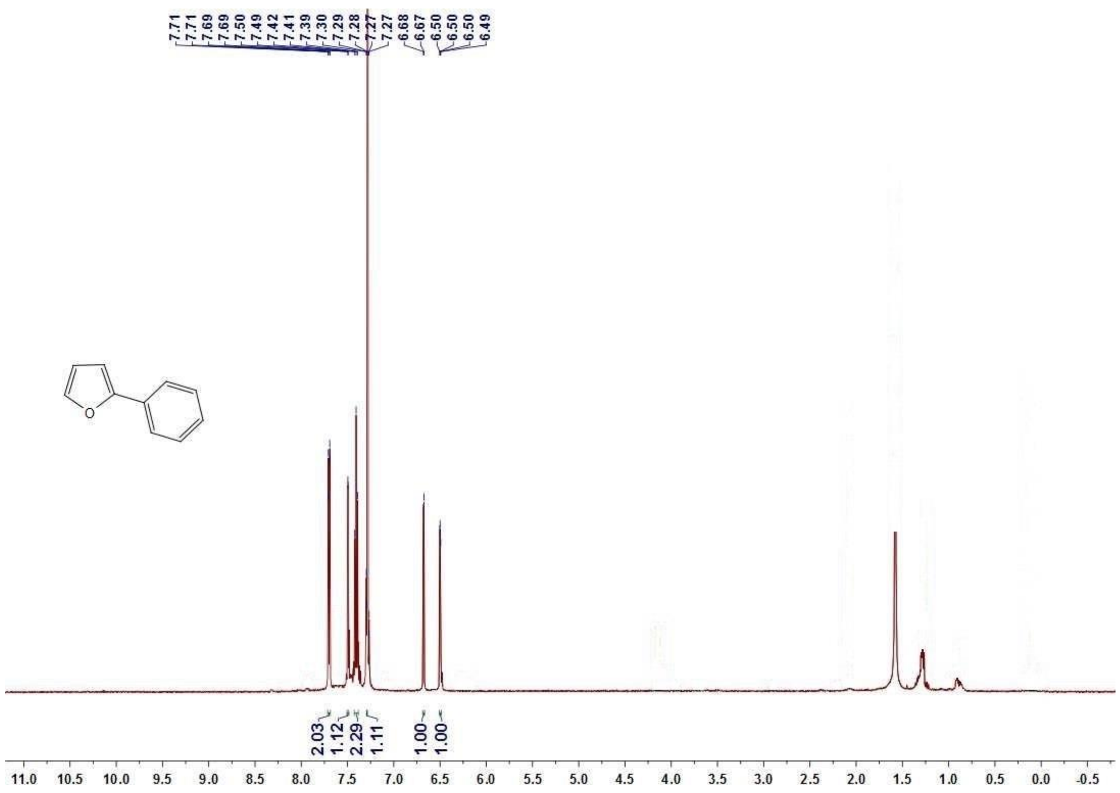

Supplementary Figure 88 ^{1}H NMR of **2-phenylfuran**

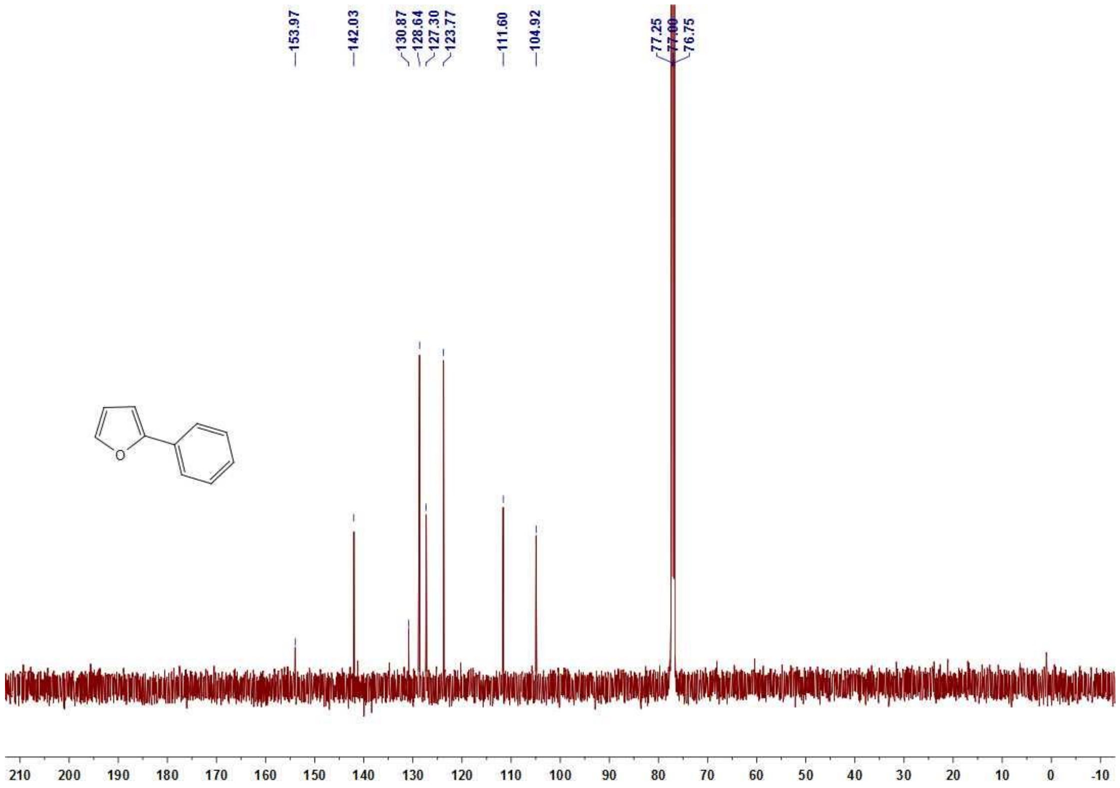

Supplementary Figure 89 ^{13}C NMR of **2-phenylfuran**

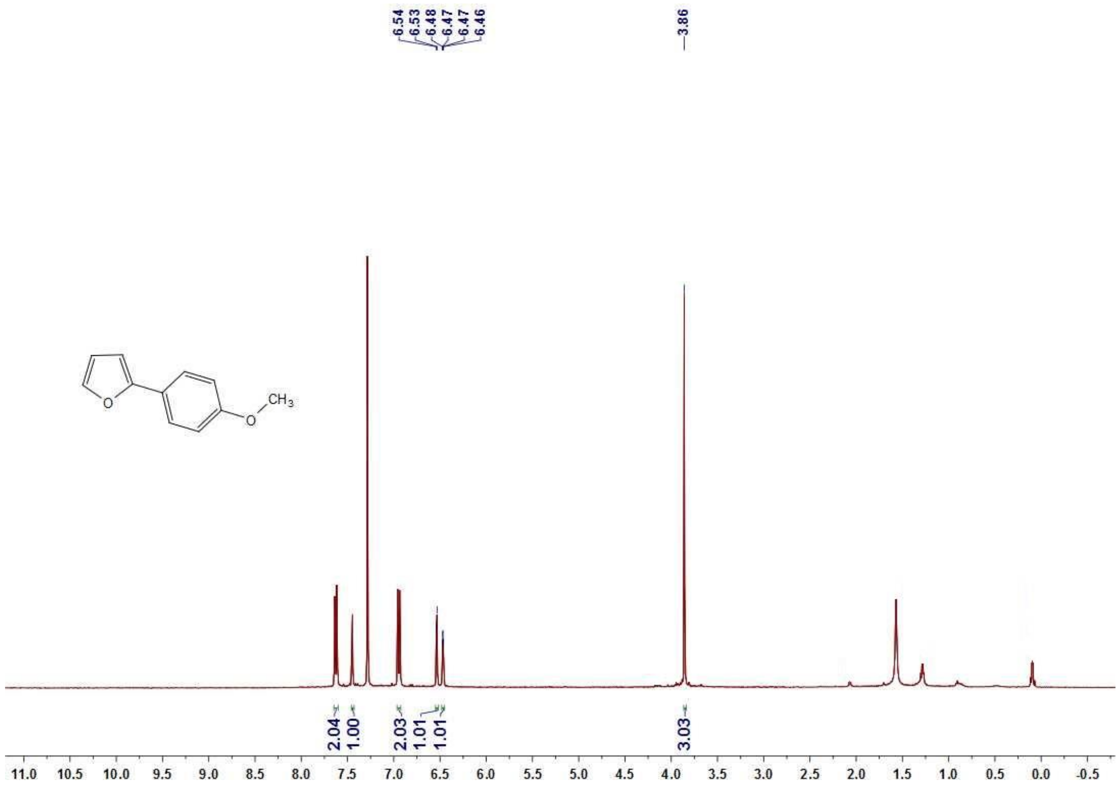

Supplementary Figure 90 ^{1}H NMR of **2-(4-methoxyphenyl)furan**

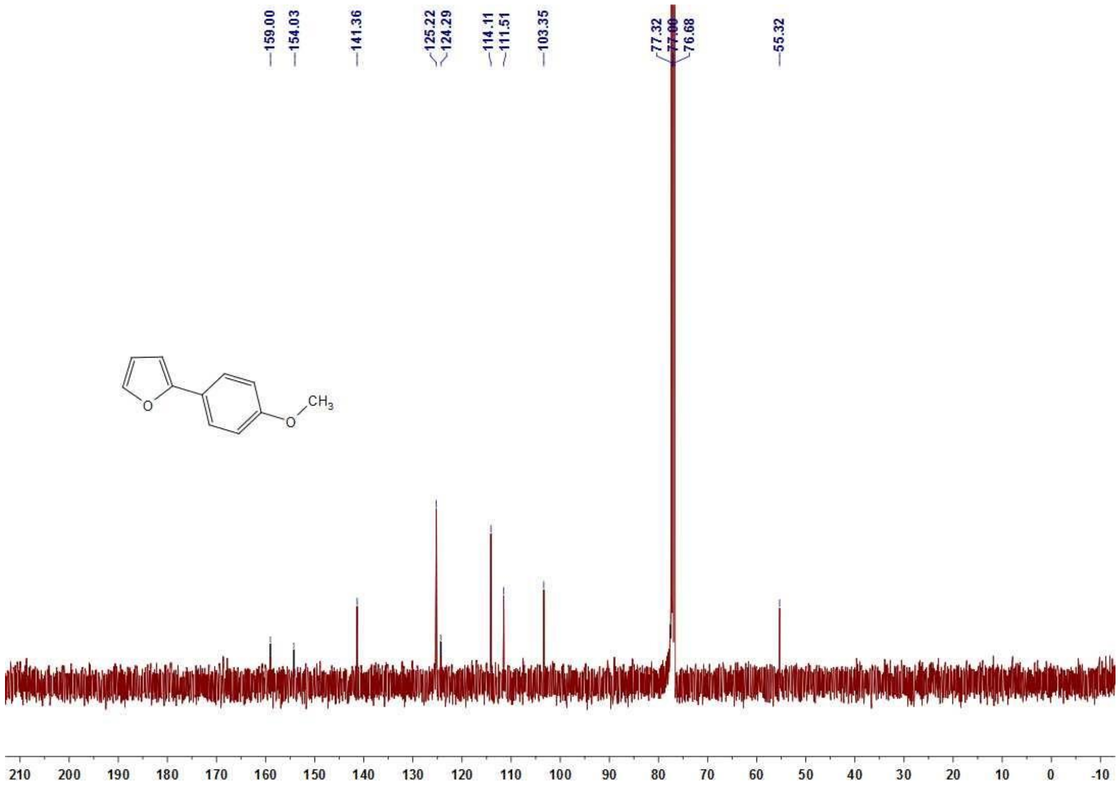

Supplementary Figure 91 ^{13}C NMR of **2-(4-methoxyphenyl)furan**

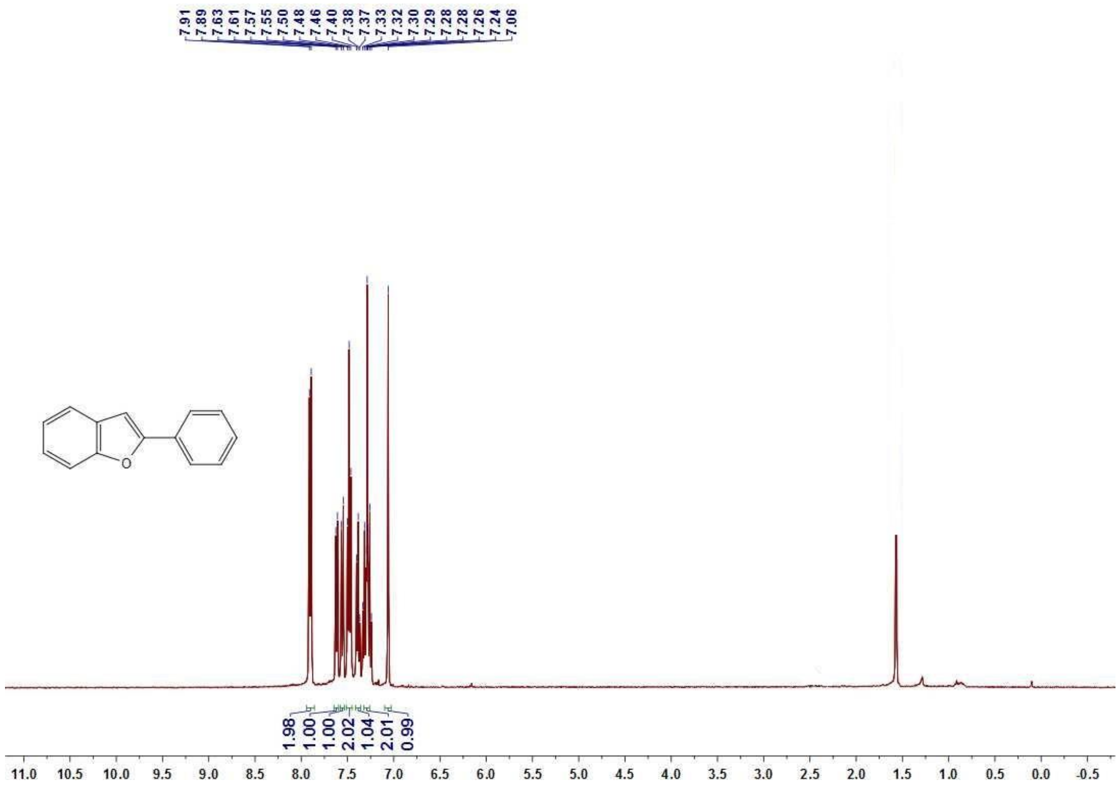

Supplementary Figure 92 ^{1}H NMR of **2-phenylbenzofuran**

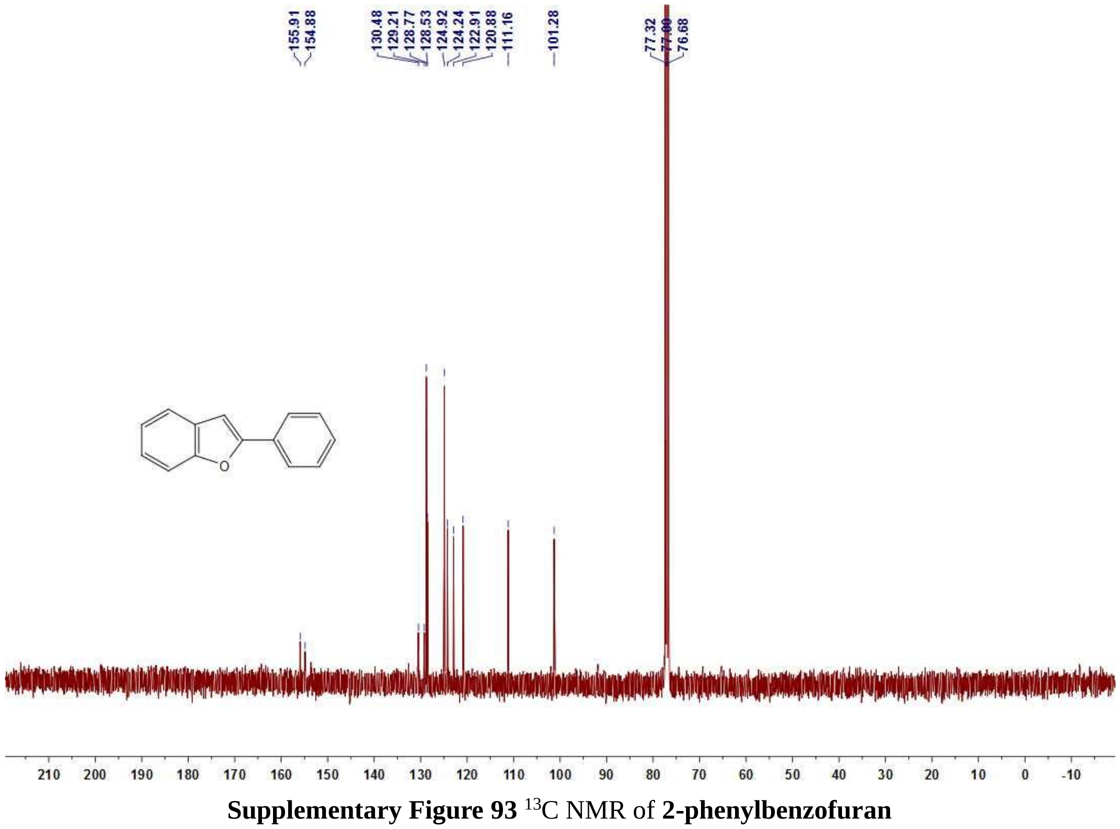

Supplementary Figure 93 ^{13}C NMR of **2-phenylbenzofuran**

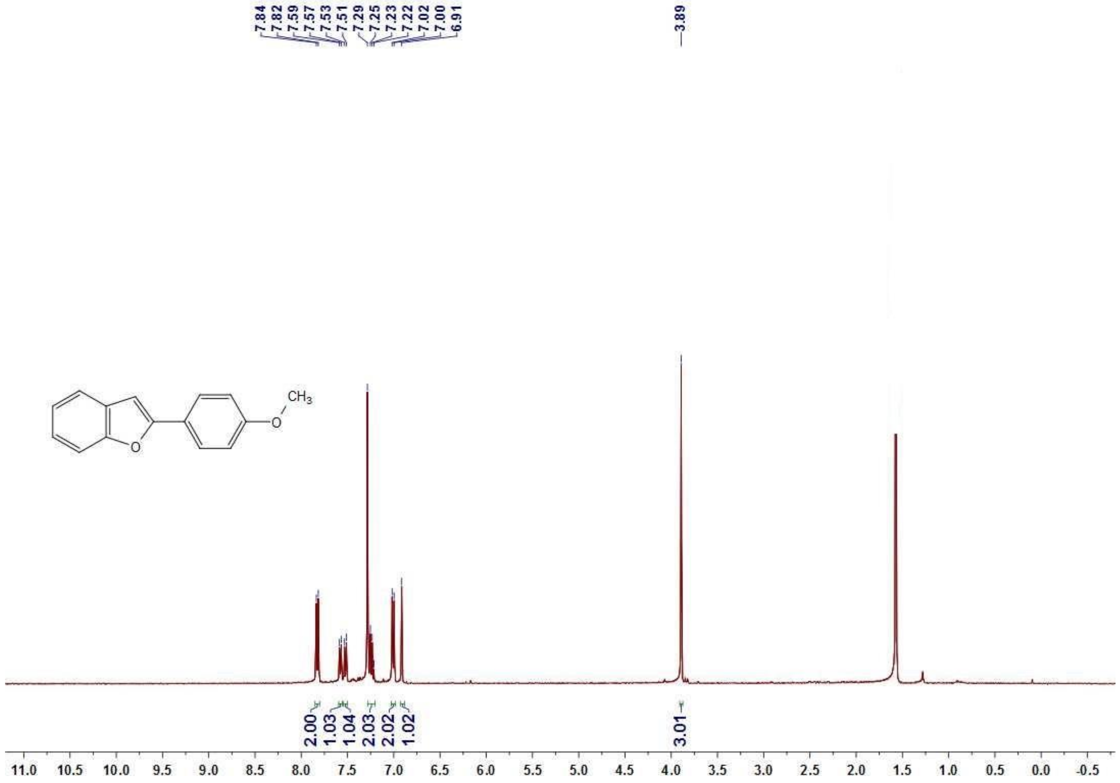

Supplementary Figure 94 ^{1}H NMR of **2-(4-methoxyphenyl)benzofuran**

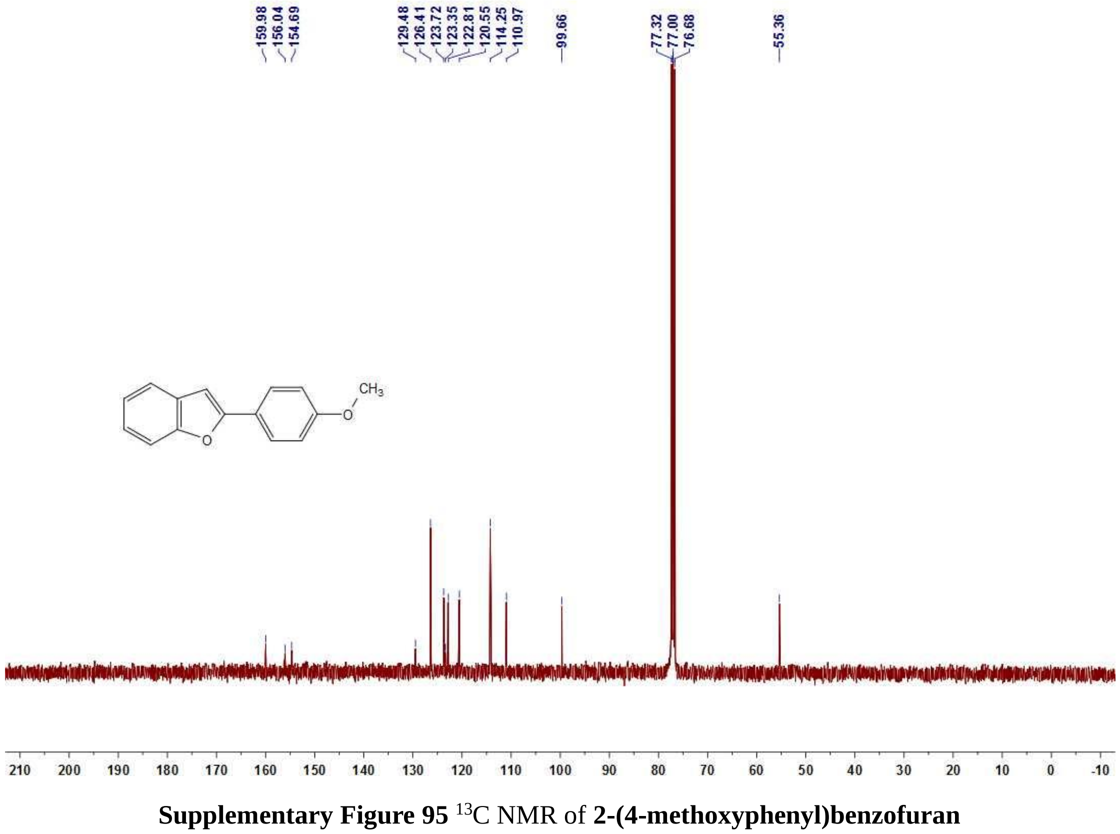

Supplementary Figure 95 ^{13}C NMR of **2-(4-methoxyphenyl)benzofuran**

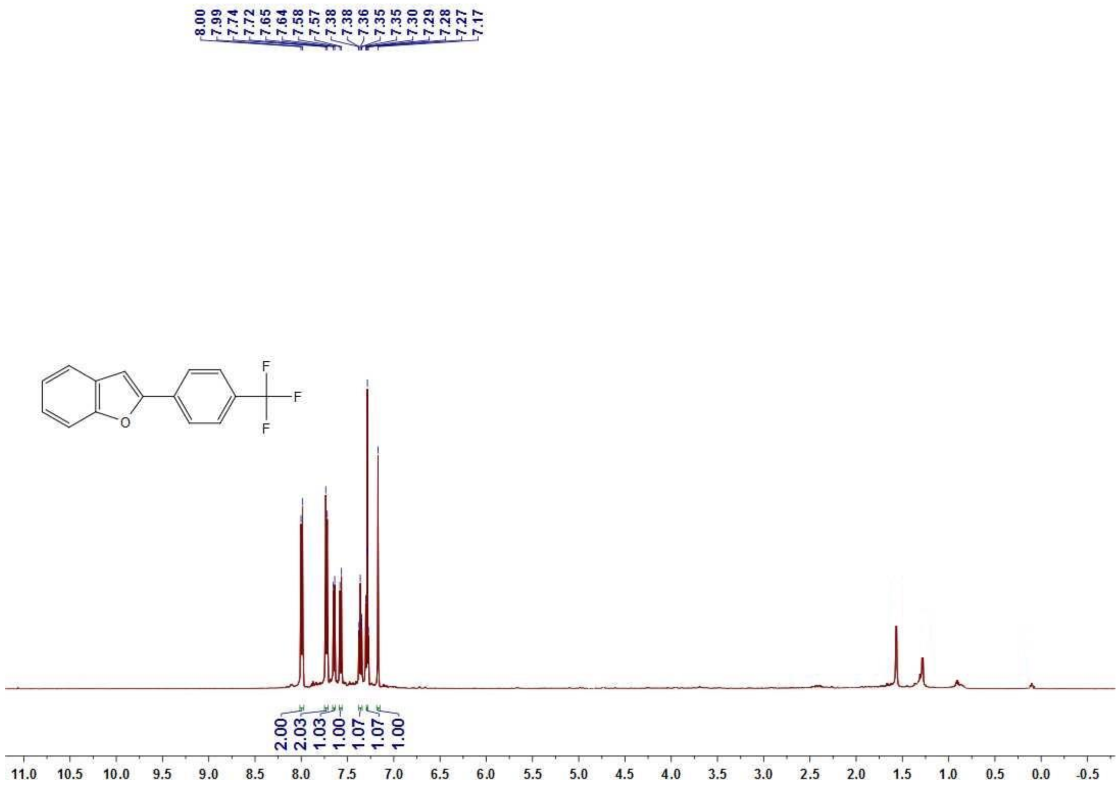

Supplementary Figure 96 ^{1}H NMR of **2-(4-(trifluoromethyl)phenyl)benzofuran**

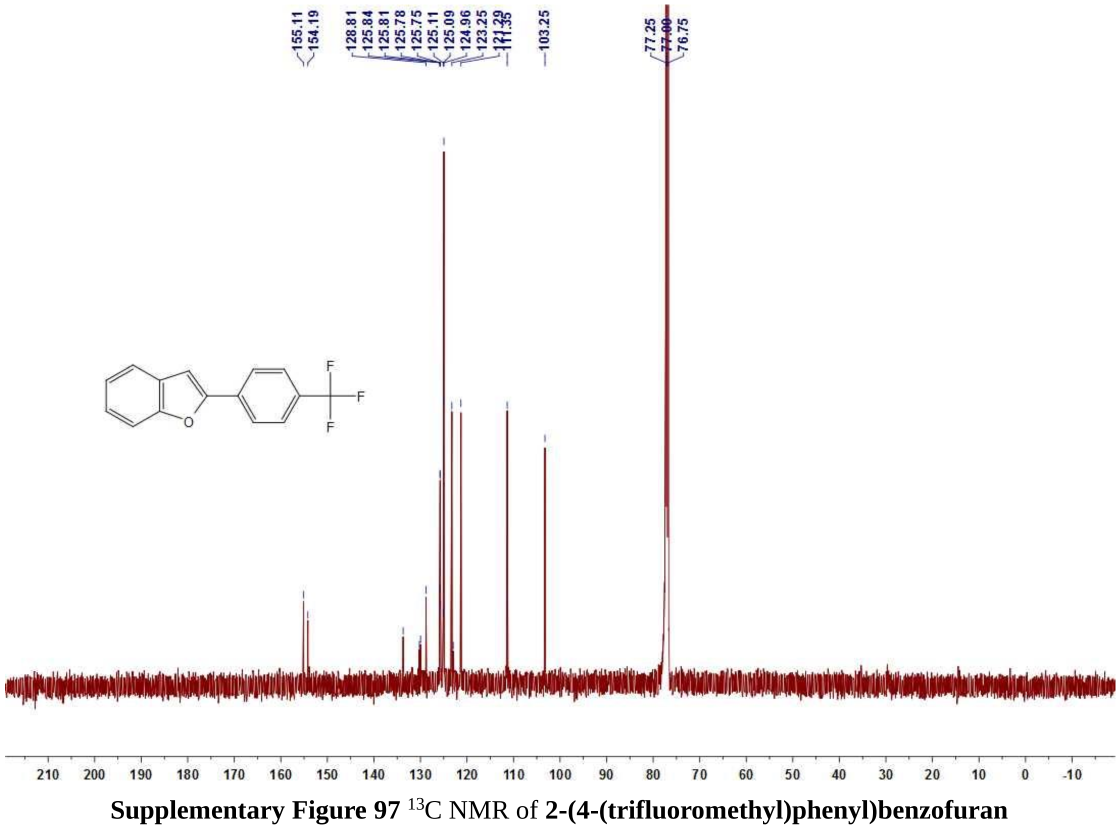

Supplementary Figure 97 ^{13}C NMR of **2-(4-(trifluoromethyl)phenyl)benzofuran**

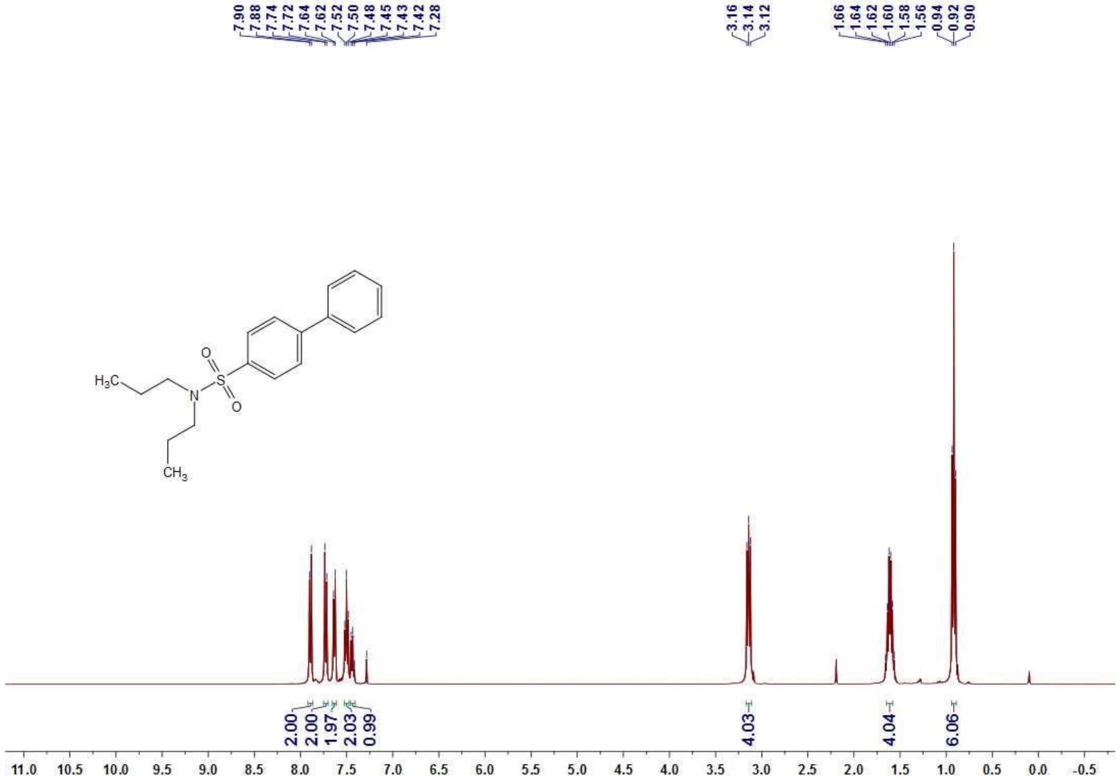

Supplementary Figure 98 ^{1}H NMR of **N,N-dipropyl-[1,1'-biphenyl]-4-sulfonamide**

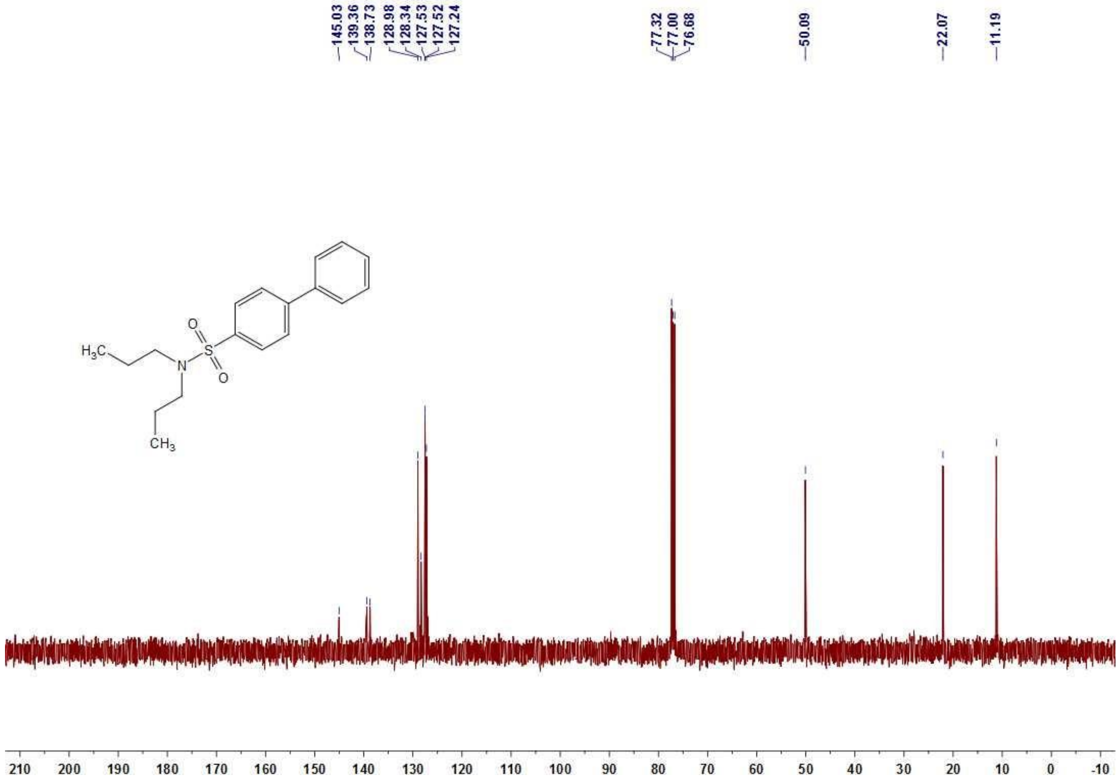

Supplementary Figure 99 ^{13}C NMR of **N,N-dipropyl-[1,1'-biphenyl]-4-sulfonamide**

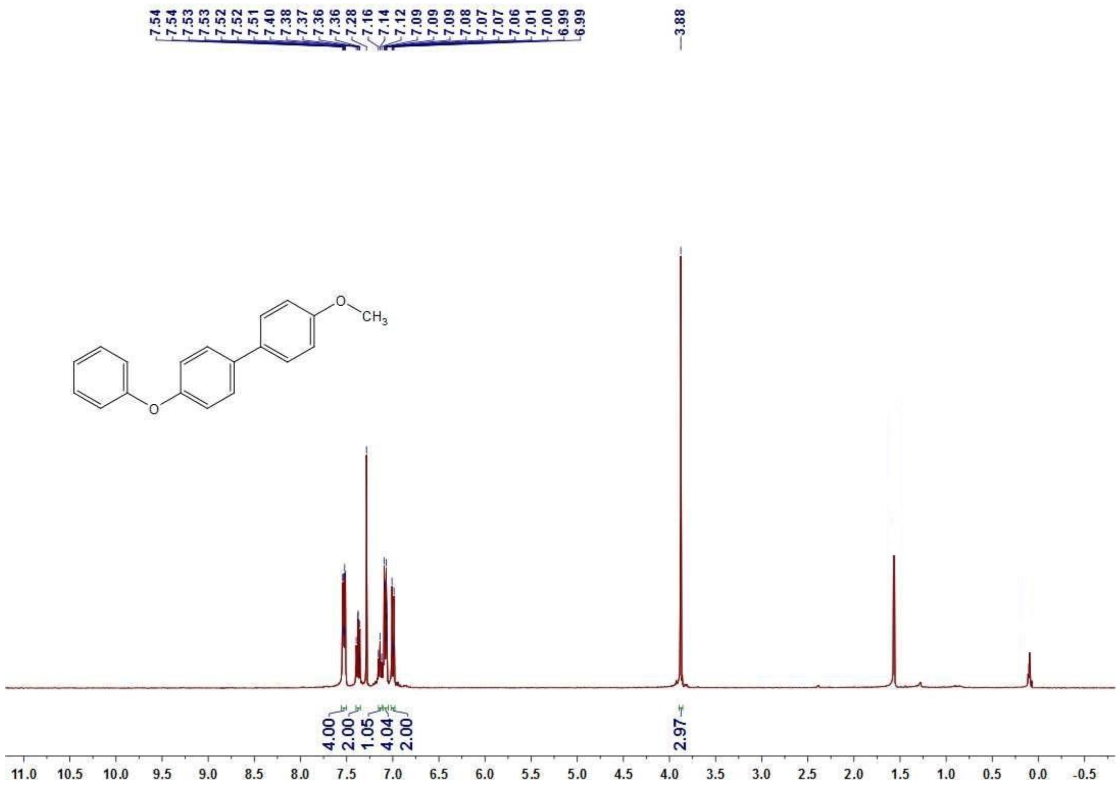

Supplementary Figure 100 ^{1}H NMR of **4-methoxy-4'-phenoxy-1,1'-biphenyl**

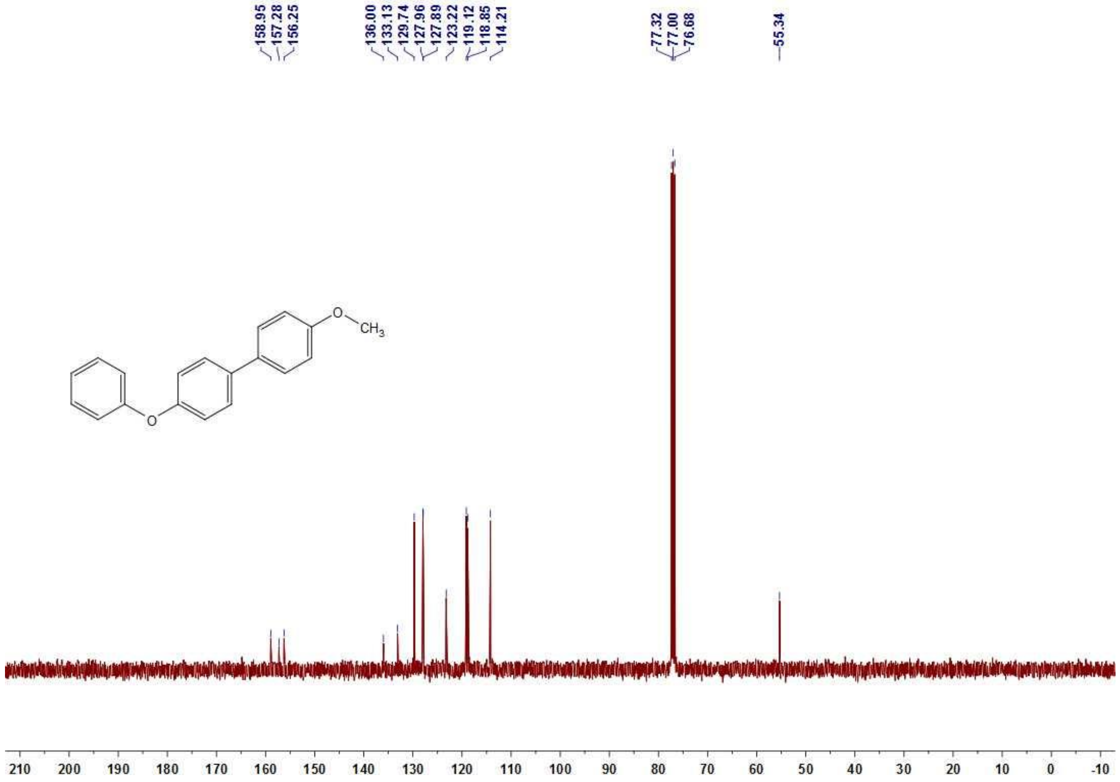

Supplementary Figure 101 ^{13}C NMR of **4-methoxy-4'-phenoxy-1,1'-biphenyl**

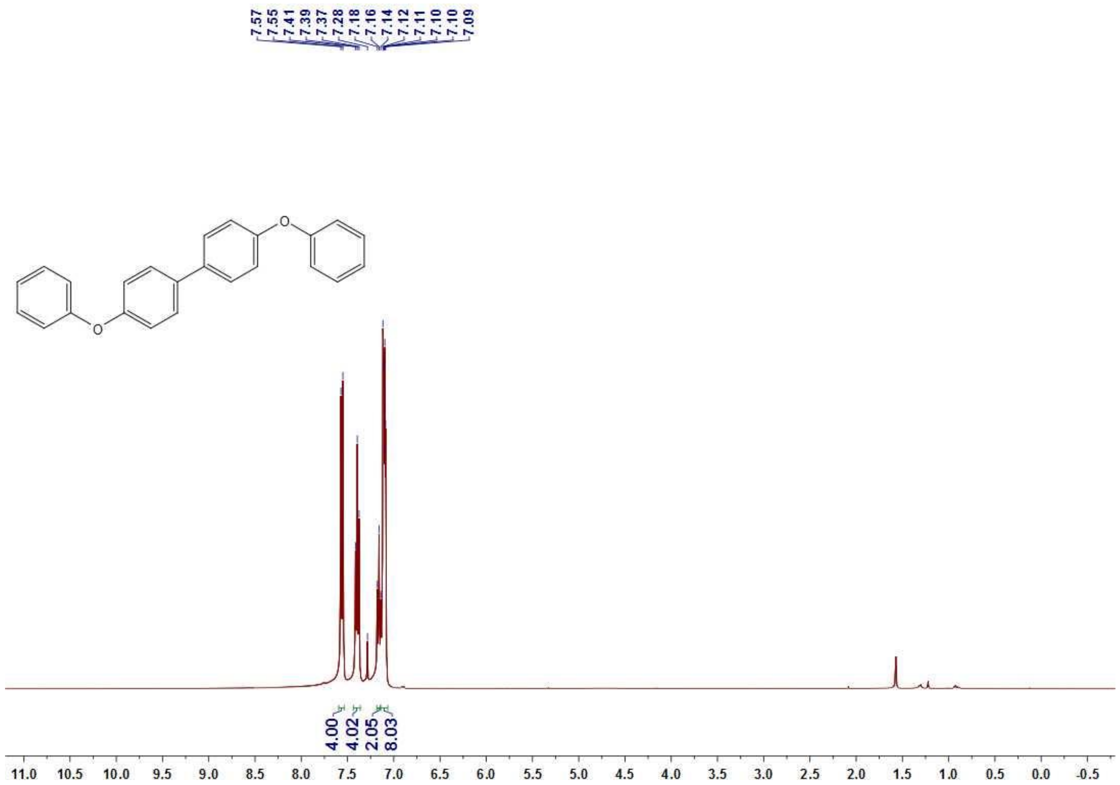

Supplementary Figure 102 ^{1}H NMR of **4,4'-diphenoxy-1,1'-biphenyl**

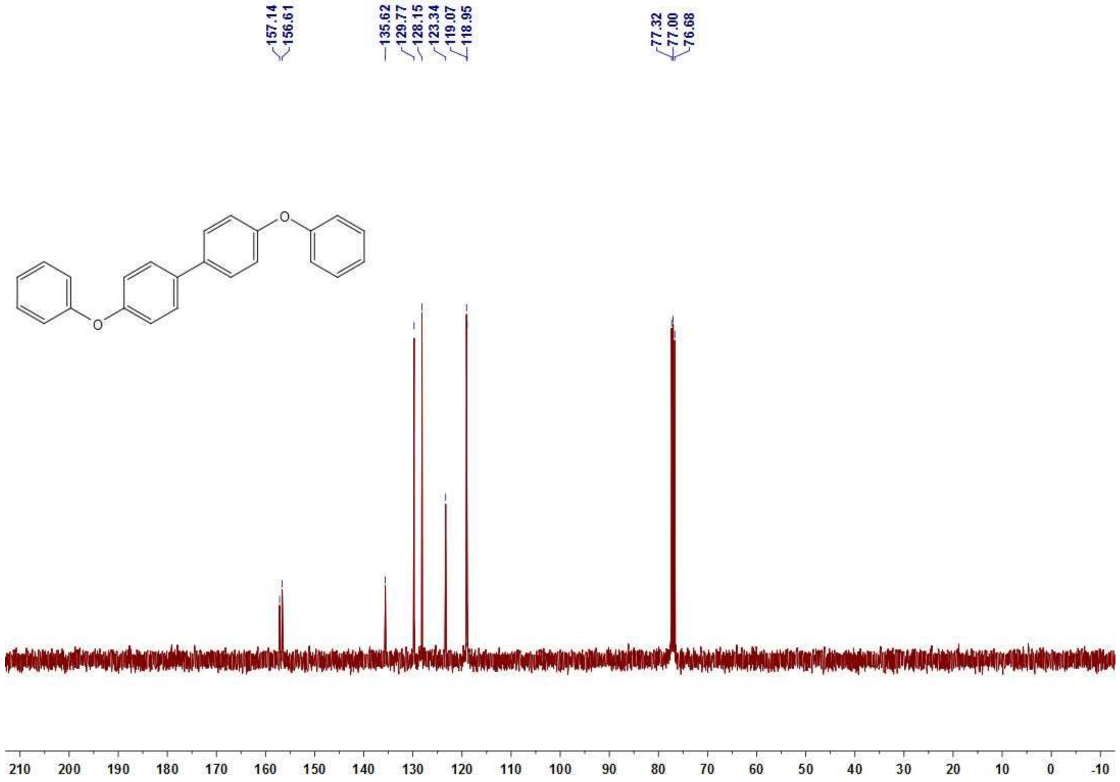

Supplementary Figure 103 ^{13}C NMR of **4,4'-diphenoxy-1,1'-biphenyl**

Ⅴ. Supplementary References

1. Cao, D.; Chen, Z.; Lv, L.; Zeng, H.; Peng, Y.; Li, C.-J. Light-Driven Metal-Free Direct Deoxygenation of Alcohols under Mild Conditions. *iScience* **23**, 101419 (2020).
2. Guha, S.; Sekar, G. Metal-Free Halogen(I) Catalysts for the Oxidation of Aryl(heteroaryl)methanes to Ketones or Esters: Selectivity Control by Halogen Bonding. *Chem. Eur. J.* **24**, 14171-14182 (2018).
3. Romero, N. ; Nicewicz, D. Organic Photoredox Catalysis. *Chem. Rev.* **116**, 10075−10166 (2016).
4. Elgrishi, N.; Chambers, M.; Wang, X.; Fontecave, M. Molecular Polypyridine-based Metal Complexes as Catalysts for the Reduction of CO_2. *Chem. Soc. Rev.* **46**, 761-796 (2017).
5. (a) Becke, A. Density-Functional Thermochemistry. III. The Role of Exact Exchange. *J. Chem. Phys.* **98**, 5648-5652 (1993); (b) Lee, C.; Yang, W.; Parr, R. Development of the Colle-Salvetti Correlation-Energy Formula into a Functional of the Electron Density. *Phys. Rev. B*. **37**, 785-789 (1988).
6. Scalmani, G.; Frisch, M. Continuous Surface Charge Polarizable Continuum Models of Solvation. I. General Formalism. *J. Chem. Phys.* **132**, 114110 (2010).
7. Kerr, C.; Mitchell, C.; Headrick, J.; Eaton, B.; Netzel, T. Synthesis and Photophysics of a 1-Pyrenyl Substituted 2'-Deoxyuridine-5-Carboxamide Nucleoside: Electron Transfer Products as CIS INDO/S Excited States. *J. Phys. Chem. B.* **104**, 1637-1650 (2000).
8. Aloïse, S.; Ruckebusch, C.; Blanchet, L.; Réhault, J.; Buntinx, G.; Huvenne, J.-P. The Benzophenone S1(n, π *) → T1(n, π *) States Intersystem Crossing Reinvestigated by Ultrafast Absorption Spectroscopy and Multivariate Curve Resolution. *J. Phys. Chem. A*. **112**, 224-231(2008).
9. Zeng, H.; Cao, D.; Qiu, Z.; Li, C.-J. Palladium-Catalyzed Formal Cross-Coupling of Diaryl Ethers with Amines: Slicing the 4-O-5 Linkage in Lignin Models. *Angew. Chem. Int. Ed.* **57**, 3752-3757 (2018).
10. Zhou, T.; Ji. C.-L.; Hong, X.; Szostak, M. Palladium-Catalyzed Decarbonylative Suzuki–Miyaura Cross-Coupling of Amides by Carbon–Nitrogen Bond Activation. *Chem. Sci.* **10**, 9865-9871 (2019).
11. Deng, Z.; Zhao, M.; Wang, F.; Tang, P. Selective C-H Trifluoromethoxylation of (Hetero)arenes as Limiting Reagent. *Nat. Commun.* **11**, 2569 (2020).
12. Liu, Z.; Wang, P.; Chen, Y.; Yan, Z.; Chen, S.; Chen, W.; Mu, T. Small Organic Molecules with Tailored Structures: Initiators in the Transition-Metal-Free C–H Arylation of Unactivated Arenes. *RSC Adv.* **10**, 14500-14509 (2020).
13. Guo, L.; Srimontree, W.; Zhu, C.; Maity, B.; Liu, X.; Cavallo, L.; Rueping, M. Nickel-Catalyzed Suzuki–Miyaura Cross-Couplings of Aldehydes. *Nat. Commun.* **10**, 1957 (2019).
14. Luo, Y.-C.; Tong, F.-F.; Zhang, Y.; He, C.-Y.; Zhang, X. Visible-Light-Induced Palladium-Catalyzed Selective Defluoroarylation of Trifluoromethylarenes with Arylboronic Acids. *J. Am. Chem. Soc*. **143**, 13971-13979 (2021).
15. dos Santos, B.; da Silva, B.; de Oliveira A.; Sarragiotto, M.; Domingues N. Anchored Pd(0) Nanoparticles on Synthetic Talc for the Synthesis of Biaryls and a Precursor of Angiotensin II Inhibitors. *Synthesis*, **53**, 933-942 (2021).
16. Dhital, R.; Sen, A.; Sato, T.; Hu, H.; Ishii, R.; Hashizume, D.; Takaya, H.; Uozumi, Y.; Yamada, Y. Activator-Promoted Aryl Halide-Dependent Chemoselective Buchwald–Hartwig and Suzuki–Miyaura Type Cross-Coupling Reactions. *Org. Lett.* **22**, 4797-4801 (2020).
17. Pinxterhuis, E.; Visser, P.; Esser, I.; Gualtierotti, J.-B.; Feringa, B. Fast, Efficient and Low E-Factor

One-Pot Palladium-Catalyzed Cross-Coupling of (Hetero)Arenes. *Angew. Chem. Int. Ed.* **57**, 9452-9455 (2018).

18. Gong, X.; Wu, J.; Meng, Y.; Zhang, Y.; Ye, L.-W.; Zhu, C. Ligand-Free Palladium Catalyzed Ullmann Biaryl Synthesis: 'Household' Reagents and Mild Reaction Conditions. *Green Chem.* **21**, 995-999 (2019).
19. Zhou, T.; Xie, P.-P.; Ji, C.-L.; Hong, X.; Szostak, M. Decarbonylative Suzuki–Miyaura Cross-Coupling of Aroyl Chlorides. *Org. Lett.* **22**, 6434-6440 (2020).
20. Yan, Q.; Zheng, L.; Li, M.; Chen, Y. N,S-Chelating Triazole-Thioether Ligand for Highly Efficient Palladium-Catalyzed Suzuki Reaction. *J. Catal.* **376**, 101-105 (2019).
21. Chen, Q.; Wu, S.; Yan, S.; Li, C.; Abduhulam, H.; Shi, Y.; Dang, Y.; Cao, C. Suzuki–Miyaura Cross-Coupling of Sulfoxides. *ACS Catal*, **10**, 8168-8176 (2020).
22. Yamada, S.; Kaneda, T.; Steib, P.; Murakami, K.; Itami, K. Dehydrogenative Synthesis of 2,2′-Bipyridyls through Regioselective Pyridine Dimerization. *Angew.Chem. Int. Ed.* **58**, 8341 –8345 (2019).
23. Guo, B.; Li, H.-X.; Zha, C.-H.; Young, D.; Li, H.-Y.; Lang, J.-P. Visible-Light-Enhanced Suzuki–Miyaura Reactions of Aryl Chlorides in Water with Pd NPs Supported on a Conjugated Nanoporous Polycarbazole. *ChemSusChem* **12**, 1421-1427 (2019).
24. Gao, P.; Szostak, M. Highly Selective and Divergent Acyl and Aryl Cross-Couplings of Amides via Ir-Catalyzed C–H Borylation/N–C(O) Activation. *Org. Lett.* **22**, 6010-6015 (2020).
25. Zorba, L.; Kidonakis, M.; Saridakis, I.; Stratakis, M. Cycloisomerization of Conjugated Allenones into Furans under Mild Conditions Catalyzed by Ligandless Au Nanoparticles. *Org. Lett.* **21**, 5552-5555 (2019).
26. Gemoets, H.; Kalvet, I.; Nyuchev, A.; Erdmann, N.; Hessel, V.; Schoenebeck F.; Noël, T. Mild and Selective Base-Free C–H Arylation of Heteroarenes: Experiment and Computation. *Chem. Sci.* **8**, 1046-1055 (2017).
27. Liu, Y.; Lu, T.; Tang, W.-F.; Gao J. Transition-Metal-Free Base Catalyzed Intramolecular Cyclization of 2-Ynylphenols for Efficient and Facile Synthesis of 2-Substituted Benzo[b]furans. *RSC Adv.* **8**, 28637-28641(2018).
28. Malapit, C.; Bour, J.; Brigham, C.; Sanford, M. Base-Free Nickel-Catalysed Decarbonylative Suzuki–Miyaura Coupling of Acid Fluorides. *Nature* **563**, 100-104 (2018).
29. Lv, L.; Qiu, Z.; Li, J.; Liu, M.; Li, C.-J. N_2H_4 as Traceless Mediator for Homo- and Cross- Aryl Coupling. *Nat. Commun.* **9**, 4739 (2018).
