# Supplementary material for: Light-driven transition-metal-free direct decarbonylation of unstrained diaryl ketones via a dual C–C bond cleavage
Source: Nat Commun. 2022 Apr 4;13:1805. doi: 10.1038/s41467-022-29327-z (PMC8979990; doi:10.1038/s41467-022-29327-z)
Supplement: Supplementary file 2 — Source Data [file 41467_2022_29327_MOESM2_ESM.zip › Cartesian Coordinates.doc]

**Cartesian Coordinate**

**1**

24

C	2.68450	-1.50520	0.75090

C	1.43730	-0.87860	0.68690

C	1.29860	0.35630	0.03000

C	2.42780	0.94900	-0.56320

C	3.66610	0.31020	-0.51860

C	3.79740	-0.91770	0.14200

H	2.78540	-2.45150	1.27460

H	0.58190	-1.33890	1.17020

H	2.32150	1.90540	-1.06530

H	4.52860	0.76790	-0.99460

H	4.76400	-1.41200	0.18340

C	-0.00000	1.10180	0.00010

O	-0.00000	2.33510	0.00000

C	-1.29860	0.35630	-0.02990

C	-1.43720	-0.87860	-0.68680

C	-2.42790	0.94900	0.56310

C	-2.68440	-1.50530	-0.75080

H	-0.58170	-1.33900	-1.16990

C	-3.66610	0.31020	0.51830

H	-2.32160	1.90540	1.06510

C	-3.79740	-0.91770	-0.14220

H	-2.78520	-2.45160	-1.27450

H	-4.52870	0.76790	0.99420

H	-4.76390	-1.41210	-0.18360

**Shv**

24

C	2.69940	-1.52430	0.69780

C	1.44720	-0.91050	0.64140

C	1.30130	0.34360	0.02630

C	2.43190	0.97190	-0.52300

C	3.67560	0.34830	-0.48430

C	3.81190	-0.90180	0.12850

H	2.80510	-2.48780	1.18760

H	0.59170	-1.39580	1.09860

H	2.31700	1.94760	-0.98370

H	4.54000	0.83510	-0.92640

H	4.78360	-1.38600	0.16510

C	-0.00010	1.08640	0.00020

O	0.00000	2.31640	0.00030

C	-1.30140	0.34360	-0.02610

C	-1.44700	-0.91060	-0.64100

C	-2.43220	0.97200	0.52260

C	-2.69910	-1.52450	-0.69760

H	-0.59140	-1.39600	-1.09790

C	-3.67580	0.34830	0.48370

H	-2.31750	1.94780	0.98320

C	-3.81190	-0.90200	-0.12880

H	-2.80460	-2.48820	-1.18710

H	-4.54040	0.83510	0.92540

H	-4.78350	-1.38620	-0.16540

**T1**

24

C	2.79970	-1.53890	0.60060

C	1.52520	-0.98580	0.57910

C	1.31010	0.29930	0.01410

C	2.43720	1.02000	-0.47300

C	3.71020	0.45800	-0.42700

C	3.90310	-0.82430	0.10150

H	2.94260	-2.52990	1.02270

H	0.69370	-1.53600	1.00610

H	2.29360	2.00130	-0.91300

H	4.55580	1.01890	-0.81570

H	4.89630	-1.26210	0.13180

C	-0.00000	0.91740	0.00010

O	0.00000	2.24660	0.00010

C	-1.31010	0.29930	-0.01410

C	-1.52510	-0.98590	-0.57890

C	-2.43730	1.02000	0.47290

C	-2.79960	-1.53900	-0.60060

H	-0.69360	-1.53620	-1.00580

C	-3.71030	0.45800	0.42680

H	-2.29370	2.00140	0.91280

C	-3.90310	-0.82440	-0.10160

H	-2.94250	-2.53010	-1.02250

H	-4.55590	1.01890	0.81530

H	-4.89630	-1.26220	-0.13200

**TS0**

34

C	3.89490	-0.52060	-0.91580

C	2.58380	-0.75090	-0.48320

C	1.89610	0.22400	0.25530

C	2.55240	1.42990	0.56120

C	3.85470	1.66310	0.12200

C	4.53180	0.68680	-0.62050

H	4.41590	-1.28930	-1.47980

H	2.11160	-1.69940	-0.71310

H	2.02830	2.17820	1.14540

H	4.34500	2.60340	0.35970

H	5.54870	0.86560	-0.95920

C	0.49990	0.00760	0.80710

O	0.20420	0.56100	1.90690

C	-0.25750	-1.23780	0.39530

C	-0.50570	-1.56660	-0.94600

C	-0.71990	-2.09540	1.40300

C	-1.20660	-2.72960	-1.27160

H	-0.16760	-0.90230	-1.73500

C	-1.41440	-3.26540	1.07790

H	-0.53420	-1.84270	2.44240

C	-1.66030	-3.58540	-0.26020

H	-1.40110	-2.96720	-2.31390

H	-1.76330	-3.92280	1.86960

H	-2.20340	-4.49130	-0.51510

O	-0.37190	1.39050	-0.14550

S	-2.76400	1.37840	-0.06760

C	-2.80980	1.72470	-1.85000

H	-3.75750	2.20650	-2.10620

H	-1.97380	2.37090	-2.12920

H	-2.73200	0.76980	-2.37390

C	-2.85910	3.06320	0.60220

H	-2.03910	3.66750	0.20840

H	-3.82180	3.51170	0.34070

H	-2.77340	2.98780	1.68850

**A(T)**

25

C	2.65500	-1.56680	-0.86170

C	1.44400	-0.87960	-0.83590

C	1.31610	0.27840	-0.04710

C	2.42600	0.77640	0.65980

C	3.63330	0.08080	0.63270

C	3.74990	-1.09030	-0.12680

H	2.74950	-2.47200	-1.45410

H	0.59650	-1.24540	-1.40690

H	2.33180	1.68900	1.24040

H	4.48400	0.45270	1.19600

H	4.69370	-1.62730	-0.15290

C	-0.00000	1.07440	-0.00010

O	-0.20780	1.79880	1.14270

C	-1.31600	0.27820	0.04700

C	-2.42630	0.77660	-0.65930

C	-1.44370	-0.88010	0.83540

C	-3.63360	0.08100	-0.63210

H	-2.33230	1.68950	-1.23940

C	-2.65470	-1.56730	0.86120

H	-0.59610	-1.24620	1.40590

C	-3.74990	-1.09050	0.12690

H	-4.48450	0.45320	-1.19480

H	-2.74910	-2.47270	1.45330

H	-4.69370	-1.62740	0.15310

O	0.20760	1.79870	-1.14290

Free-DMSO

10

O	0.00010	1.51920	0.37770

S	0.00000	0.21900	-0.44400

C	-1.36940	-0.80640	0.18890

H	-1.34230	-1.78210	-0.30540

H	-1.27170	-0.91490	1.27290

H	-2.30100	-0.29260	-0.06000

C	1.36930	-0.80650	0.18890

H	1.27150	-0.91510	1.27290

H	1.34210	-1.78220	-0.30540

H	2.30100	-0.29290	-0.05990

Free-DMS

9

S	0.00000	0.66330	-0.00000

C	1.39680	-0.51490	0.00000

H	1.37590	-1.14390	0.89490

H	1.37590	-1.14410	-0.89480

H	2.31960	0.07150	-0.00020

C	-1.39680	-0.51490	0.00000

H	-1.37590	-1.14410	-0.89480

H	-1.37590	-1.14390	0.89490

H	-2.31960	0.07150	-0.00020

Free-DBU

27

C	2.05420	-0.09930	-1.08720

C	2.12230	1.24860	-0.34900

C	0.96550	1.53990	0.65390

C	2.00790	-1.34780	-0.19300

C	-0.32980	0.79240	0.39190

C	0.95670	-1.25270	0.93360

H	2.91910	-0.17440	-1.75810

H	2.12960	2.03990	-1.10800

H	0.72250	2.60460	0.63440

H	2.98620	-1.52170	0.27440

H	0.68060	-2.25360	1.27820

H	1.38390	-0.73840	1.79870

H	1.80060	-2.22150	-0.82360

H	1.17030	-0.09950	-1.73710

H	3.07550	1.32770	0.18870

H	1.29750	1.31560	1.67370

N	-0.28090	-0.57310	0.53600

N	-1.38540	1.48070	0.06040

C	-1.41530	-1.42120	0.13920

C	-2.37670	-0.65180	-0.76050

C	-2.62640	0.73360	-0.16180

H	-1.02150	-2.30080	-0.38220

H	-1.93580	-1.78030	1.03860

H	-1.94260	-0.54110	-1.76210

H	-3.31200	-1.21230	-0.86530

H	-3.16810	0.62660	0.79200

H	-3.27200	1.32640	-0.82010

Free-DBU-Cation

27

C	2.08740	-0.11130	-1.07840

C	2.14290	1.24580	-0.35890

C	0.96180	1.56620	0.60750

C	2.01670	-1.34580	-0.16810

C	-0.29990	0.77570	0.38030

C	0.95130	-1.24670	0.94960

H	2.97530	-0.20260	-1.71390

H	2.17670	2.02710	-1.12490

H	0.68660	2.61950	0.53070

H	2.97490	-1.51140	0.33700

H	0.65580	-2.23590	1.30090

H	1.33830	-0.68070	1.79960

H	1.81040	-2.23080	-0.77890

H	1.22990	-0.12090	-1.76270

H	3.07380	1.32430	0.21300

H	1.27000	1.40140	1.64740

N	-0.26530	-0.56990	0.47530

N	-1.44660	1.42870	0.19840

C	-1.44080	-1.40000	0.16410

C	-2.37330	-0.66370	-0.78030

C	-2.63730	0.73030	-0.22770

H	-1.06390	-2.33110	-0.26650

H	-1.92600	-1.64440	1.11930

H	-1.91490	-0.59070	-1.77260

H	-3.30720	-1.22280	-0.87860

H	-3.29940	0.70670	0.65520

H	-3.14980	1.37830	-0.95020

A

25

C	-2.61250	-1.40220	-1.14580

C	-1.44180	-0.64350	-1.06190

C	-1.26940	0.30940	-0.04400

C	-2.29600	0.47600	0.89340

C	-3.46950	-0.28570	0.81860

C	-3.63350	-1.22700	-0.20230

H	-2.72850	-2.13230	-1.94330

H	-0.65500	-0.79310	-1.79770

H	-2.16460	1.21400	1.67900

H	-4.25480	-0.14340	1.55740

H	-4.54310	-1.81890	-0.26320

C	0.00000	1.19620	-0.00010

O	0.00430	2.09030	1.04800

C	1.26940	0.30950	0.04390

C	1.44190	-0.64330	1.06200

C	2.29590	0.47590	-0.89350

C	2.61250	-1.40200	1.14600

H	0.65500	-0.79280	1.79790

C	3.46950	-0.28590	-0.81860

H	2.16460	1.21380	-1.67920

C	3.63350	-1.22700	0.20240

H	2.72860	-2.13200	1.94360

H	4.25470	-0.14360	-1.55750

H	4.54300	-1.81890	0.26330

O	-0.00430	2.09020	-1.04810

**TS1**

25

C	-2.58270	-1.72870	-0.57850

C	-1.48090	-0.91880	-0.88550

C	-1.31620	0.28140	-0.20010

C	-2.22760	0.70930	0.75740

C	-3.33370	-0.10260	1.05230

C	-3.51160	-1.32070	0.38780

H	-2.71810	-2.67340	-1.10020

H	-0.76920	-1.22790	-1.64430

H	-2.05670	1.65110	1.26920

H	-4.05030	0.21780	1.80530

H	-4.36780	-1.94850	0.61940

C	0.14740	1.41790	-0.46440

O	0.00880	2.41090	0.34510

C	1.32200	0.45770	-0.10630

C	1.75700	0.34270	1.22240

C	1.98660	-0.28100	-1.09520

C	2.82290	-0.49720	1.55820

H	1.25210	0.92460	1.98720

C	3.06290	-1.11430	-0.76540

H	1.65690	-0.19130	-2.12500

C	3.48330	-1.22960	0.56360

H	3.14240	-0.57740	2.59460

H	3.57300	-1.67180	-1.54750

H	4.31630	-1.87830	0.82180

O	-0.10310	1.49890	-1.76490

**B**

25

C	4.46100	0.02520	-1.24580

C	3.05400	-0.01160	-1.27060

C	2.42310	-0.13020	-0.04980

C	3.03930	-0.21630	1.18100

C	4.44660	-0.17820	1.17920

C	5.14610	-0.05830	-0.02760

H	5.01110	0.11800	-2.17900

H	2.50930	0.05090	-2.20890

H	2.48340	-0.30870	2.11010

H	4.98570	-0.24340	2.12110

H	6.23210	-0.02990	-0.01880

C	-2.68330	-1.74560	-0.04260

O	-2.61160	-2.35910	1.06010

C	-2.52040	-0.21670	0.01270

C	-2.28970	0.43430	1.23470

C	-2.59360	0.55930	-1.15460

C	-2.13460	1.82280	1.29150

H	-2.23120	-0.16660	2.13680

C	-2.43970	1.94870	-1.10460

H	-2.77590	0.05750	-2.09960

C	-2.20810	2.58570	0.12010

H	-1.95700	2.31050	2.24700

H	-2.49850	2.53470	-2.01860

H	-2.08690	3.66500	0.16150

O	-2.87290	-2.25380	-1.18420

**TS2**

25

C	2.04000	-1.25240	1.53350

C	1.48030	-0.00580	1.28080

C	1.20030	0.42680	-0.05460

C	1.68130	-0.40400	-1.11150

C	2.24270	-1.64860	-0.85190

C	2.41230	-2.09340	0.47080

H	2.21160	-1.56970	2.55940

H	1.22130	0.66180	2.09570

H	1.57610	-0.04350	-2.12930

H	2.57510	-2.27370	-1.67760

H	2.85960	-3.06360	0.66950

C	0.96160	1.93890	-0.33100

O	0.96090	2.29600	-1.53980

C	-0.97590	-0.01830	-0.08620

C	-1.41240	-1.14230	-0.77010

C	-1.81960	0.77660	0.67590

C	-2.77400	-1.48280	-0.69240

H	-0.72960	-1.74940	-1.35940

C	-3.17820	0.42820	0.74640

H	-1.43830	1.65210	1.19550

C	-3.65270	-0.69930	0.06410

H	-3.14200	-2.35780	-1.22370

H	-3.86180	1.03810	1.33320

H	-4.70460	-0.96590	0.12230

O	0.81390	2.67030	0.68620

**C**

25

C	-2.81340	-1.49440	-0.53910

C	-1.44110	-1.22760	-0.62680

C	-0.88220	-0.09720	-0.01270

C	-1.73970	0.75330	0.70800

C	-3.10710	0.48790	0.80410

C	-3.65360	-0.63890	0.17720

H	-3.22070	-2.37490	-1.02960

H	-0.80220	-1.91110	-1.17860

H	-1.33560	1.63770	1.19390

H	-3.74780	1.16400	1.36460

H	-4.71820	-0.84480	0.24800

C	0.82370	1.77420	-0.40830

O	1.43900	2.46400	0.43830

C	0.63530	0.18510	-0.05400

C	1.34480	-0.56340	-1.14900

C	1.21800	-0.13590	1.29830

C	2.44350	-1.35600	-0.92990

H	0.98180	-0.40460	-2.16000

C	2.29860	-0.96090	1.47810

H	0.74460	0.32820	2.15840

C	2.94820	-1.57960	0.37770

H	2.93460	-1.83010	-1.77750

H	2.66840	-1.14390	2.48520

H	3.81010	-2.22100	0.53430

O	0.34050	2.12600	-1.51060

**TS3**

25

C	3.00340	-1.29730	0.61510

C	1.60750	-1.20690	0.66840

C	0.90170	-0.27680	-0.11850

C	1.65980	0.54800	-0.97670

C	3.05080	0.45370	-1.03800

C	3.73670	-0.46890	-0.23750

H	3.51420	-2.02750	1.23830

H	1.06770	-1.88220	1.32430

H	1.15680	1.28810	-1.59110

H	3.60180	1.10820	-1.70880

H	4.82000	-0.54070	-0.28210

C	-0.66160	1.68940	0.75460

O	-0.94440	2.54550	-0.07760

C	-0.60380	-0.15930	-0.06180

C	-1.32990	-0.94760	0.93050

C	-1.29340	-0.05300	-1.34640

C	-2.64930	-1.31950	0.75040

H	-0.85270	-1.15500	1.88240

C	-2.60960	-0.44170	-1.51000

H	-0.76700	0.38790	-2.18720

C	-3.32910	-1.05300	-0.45940

H	-3.16770	-1.83900	1.55420

H	-3.09020	-0.29410	-2.47550

H	-4.36160	-1.36110	-0.59720

O	-0.40810	1.64360	1.95500

**Free-CO_2_**

3

C	0.00000	0.00000	0.00000

O	0.00000	0.00000	1.16920

O	0.00000	0.00000	-1.16920

**D**

22

C	2.88840	1.20710	-0.00080

C	1.49990	1.21530	-0.00080

C	0.72110	0.00000	-0.00000

C	1.49990	-1.21530	0.00080

C	2.88840	-1.20710	0.00080

C	3.62240	-0.00000	0.00000

H	3.41780	2.15890	-0.00140

H	0.99890	2.17850	-0.00160

H	0.99890	-2.17850	0.00160

H	3.41780	-2.15890	0.00140

H	4.70850	0.00000	0.00000

C	-0.72110	0.00000	0.00000

C	-1.49990	1.21530	0.00080

C	-1.49990	-1.21530	-0.00080

C	-2.88840	1.20710	0.00080

H	-0.99890	2.17850	0.00160

C	-2.88840	-1.20710	-0.00080

H	-0.99890	-2.17850	-0.00160

C	-3.62240	-0.00000	-0.00000

H	-3.41780	2.15890	0.00140

H	-3.41780	-2.15890	-0.00140

H	-4.70850	-0.00000	-0.00000

**2**

22

C	-2.86520	1.14780	0.37500

C	-1.46820	1.14750	0.37580

C	-0.74430	-0.00000	0.00000

C	-1.46830	-1.14750	-0.37580

C	-2.86520	-1.14780	-0.37500

C	-3.57100	0.00000	-0.00000

H	-3.40170	2.04370	0.67580

H	-0.93580	2.04090	0.68910

H	-0.93580	-2.04090	-0.68910

H	-3.40170	-2.04370	-0.67580

H	-4.65750	0.00000	-0.00000

C	0.74430	-0.00000	-0.00000

C	1.46820	1.14750	-0.37580

C	1.46830	-1.14750	0.37580

C	2.86520	1.14780	-0.37500

H	0.93580	2.04090	-0.68910

C	2.86520	-1.14780	0.37500

H	0.93580	-2.04090	0.68910

C	3.57100	0.00000	0.00000

H	3.40170	2.04370	-0.67580

H	3.40170	-2.04370	0.67580

H	4.65750	0.00000	0.00000

**P1.A**

24

C	-2.77280	-1.58950	-0.49090

C	-1.50120	-1.01910	-0.45960

C	-1.29260	0.31600	-0.01040

C	-2.45900	1.04180	0.36900

C	-3.72580	0.46410	0.34440

C	-3.90240	-0.86290	-0.07950

H	-2.88730	-2.60880	-0.85380

H	-0.66590	-1.60510	-0.82660

H	-2.33350	2.07180	0.68690

H	-4.58710	1.05110	0.65720

H	-4.89100	-1.31320	-0.10200

C	0.00000	1.02040	-0.00000

O	0.00000	2.31010	-0.00000

C	1.29260	0.31600	0.01040

C	1.50120	-1.01910	0.45970

C	2.45900	1.04180	-0.36910

C	2.77280	-1.58950	0.49100

H	0.66590	-1.60510	0.82660

C	3.72580	0.46410	-0.34440

H	2.33350	2.07180	-0.68700

C	3.90240	-0.86290	0.07950

H	2.88730	-2.60880	0.85390

H	4.58720	1.05110	-0.65720

H	4.89100	-1.31320	0.10210

**P1.TS1**

24

C	-2.63390	-1.37340	-0.69240

C	-1.37270	-0.84240	-0.99480

C	-0.83470	0.18860	-0.22230

C	-1.55970	0.67830	0.86750

C	-2.81610	0.14760	1.18200

C	-3.35900	-0.88020	0.39890

H	-3.04770	-2.17270	-1.30390

H	-0.80520	-1.24760	-1.83160

H	-1.15020	1.48200	1.47860

H	-3.37290	0.53490	2.03280

H	-4.33450	-1.29440	0.64020

C	0.44020	1.72670	-1.05560

O	0.24820	2.86130	-0.66180

C	1.04210	0.56690	-0.39050

C	1.67450	-0.47000	-1.21030

C	1.66180	0.72880	0.92970

C	2.41460	-1.47970	-0.61830

H	1.48440	-0.47830	-2.28050

C	2.40710	-0.30170	1.48460

H	1.44570	1.62650	1.50200

C	2.74690	-1.46080	0.75550

H	2.79690	-2.28240	-1.24900

H	2.77580	-0.18510	2.50420

H	3.35440	-2.24520	1.19740

**P1.C**

24

C	-2.88560	-1.10740	-0.78130

C	-1.49670	-1.07980	-0.80940

C	-0.72380	-0.39040	0.18830

C	-1.49360	0.24660	1.22270

C	-2.88320	0.21280	1.23820

C	-3.61460	-0.46100	0.23900

H	-3.41600	-1.64100	-1.56830

H	-0.99530	-1.59050	-1.62550

H	-0.99020	0.77490	2.02610

H	-3.41120	0.71560	2.04680

H	-4.70030	-0.48720	0.25760

C	-0.00100	1.94150	-1.59120

O	-0.02550	2.94090	-1.02470

C	0.72880	-0.38870	0.18720

C	1.50090	-1.12590	-0.77660

C	1.49960	0.29810	1.18850

C	2.89010	-1.14780	-0.75140

H	0.99850	-1.68870	-1.55690

C	2.88900	0.26830	1.20200

H	0.99720	0.87430	1.95900

C	3.62000	-0.45030	0.23360

H	3.41970	-1.72220	-1.50970

H	3.41800	0.81450	1.98140

H	4.70580	-0.47250	0.25040

**P2.TS1**

24

C	-2.74370	-1.37420	-0.52950

C	-1.48790	-0.90930	-0.93490

C	-0.90620	0.14270	-0.22680

C	-1.51750	0.71050	0.89050

C	-2.77050	0.23150	1.28620

C	-3.38410	-0.80910	0.57920

H	-3.21710	-2.18070	-1.08300

H	-0.99740	-1.35380	-1.79380

H	-1.05110	1.52590	1.43420

H	-3.26310	0.67330	2.14810

H	-4.35560	-1.17980	0.89280

C	0.24770	1.40730	-1.21540

O	0.06160	2.60040	-1.04460

C	1.05340	0.45500	-0.44240

C	1.66520	0.85490	0.80800

C	1.58390	-0.72160	-1.09350

C	2.63330	0.06510	1.39860

H	1.34530	1.77960	1.27640

C	2.55180	-1.48940	-0.47640

H	1.21340	-0.98650	-2.07790

C	3.07450	-1.12080	0.77940

H	3.07020	0.37320	2.34470

H	2.92960	-2.37560	-0.97830

H	3.83430	-1.73340	1.25460

**P2.C**

24

C	-2.71900	-1.52980	-0.15790

C	-1.35540	-1.25110	-0.28930

C	-0.87550	0.05220	-0.10180

C	-1.78320	1.07110	0.22370

C	-3.14730	0.79330	0.35110

C	-3.61970	-0.50880	0.16070

H	-3.07560	-2.54550	-0.30600

H	-0.66500	-2.05230	-0.53630

H	-1.43190	2.08750	0.38410

H	-3.83750	1.59500	0.59880

H	-4.67960	-0.72560	0.25950

C	0.84820	1.74420	-0.79910

O	1.50840	2.65770	-0.41070

C	0.63620	0.34760	-0.18210

C	1.35210	-0.53380	-1.19650

C	1.26160	0.26950	1.20210

C	2.45830	-1.27250	-0.86510

H	0.94800	-0.54510	-2.20390

C	2.35290	-0.50590	1.46610

H	0.78710	0.85720	1.98250

C	2.98190	-1.28180	0.45030

H	2.93460	-1.87950	-1.63120

H	2.75200	-0.53410	2.47710

H	3.85330	-1.88360	0.68640

**P2.TS2**

24

C	-2.84780	-1.27740	-0.57380

C	-1.47550	-1.10290	-0.72440

C	-0.75900	-0.17350	0.07770

C	-1.50370	0.57530	1.02880

C	-2.87660	0.39710	1.17050

C	-3.56100	-0.52970	0.37290

H	-3.36570	-2.00110	-1.19720

H	-0.95490	-1.69800	-1.46520

H	-1.00810	1.30240	1.66140

H	-3.41760	0.98340	1.90820

H	-4.63260	-0.66600	0.48620

C	0.76250	1.64600	-1.42450

O	0.02920	2.54690	-1.40330

C	0.67700	0.04260	-0.10820

C	1.45900	0.69190	0.98960

C	1.45740	-0.95000	-0.91270

C	2.69790	0.23580	1.34070

H	1.02190	1.52530	1.52740

C	2.69590	-1.37330	-0.51590

H	1.02610	-1.32750	-1.83260

C	3.33100	-0.83260	0.63630

H	3.22110	0.70700	2.16900

H	3.21950	-2.11740	-1.11100

H	4.31340	-1.18030	0.93780

**P2.D**

24

C	-3.38800	-0.43650	-1.06730

C	-2.06910	-0.78580	-0.92410

C	-1.22350	-0.18840	0.11980

C	-1.89070	0.79750	0.98240

C	-3.21210	1.12420	0.81220

C	-3.99620	0.52090	-0.20940

H	-3.98040	-0.90140	-1.85120

H	-1.64930	-1.51960	-1.60080

H	-1.33230	1.28400	1.77210

H	-3.66890	1.85840	1.47090

H	-5.04070	0.78750	-0.33310

C	2.73280	1.72100	-1.31570

O	3.60650	2.40700	-1.06870

C	0.11970	-0.53390	0.28200

C	0.96350	0.05930	1.32920

C	0.78770	-1.51640	-0.58300

C	2.28330	-0.28890	1.47130

H	0.54220	0.78740	2.01100

C	2.11010	-1.84100	-0.41500

H	0.23010	-2.00060	-1.37460

C	2.89350	-1.23980	0.60800

H	2.87420	0.17210	2.25860

H	2.56790	-2.57190	-1.07670

H	3.93850	-1.50530	0.73070
